# Molecular Mechanisms in the Genesis of Seizures and Epilepsy Associated With Viral Infection

**DOI:** 10.3389/fnmol.2022.870868

**Published:** 2022-05-09

**Authors:** Wolfgang Löscher, Charles L. Howe

**Affiliations:** ^1^Department of Pharmacology, Toxicology and Pharmacy, University of Veterinary Medicine, Hannover, Germany; ^2^Center for Systems Neuroscience, Hannover, Germany; ^3^Division of Experimental Neurology, Department of Neurology, Mayo Clinic, Rochester, MN, United States; ^4^Center for Multiple Sclerosis and Autoimmune Neurology, Mayo Clinic, Rochester, MN, United States

**Keywords:** blood-brain barrier, neuroinflammation, hippocampal damage, herpesviruses, SARS-CoV-2, flaviruses, picornaviruses, status epilepticus

## Abstract

Seizures are a common presenting symptom during viral infections of the central nervous system (CNS) and can occur during the initial phase of infection (“early” or acute symptomatic seizures), after recovery (“late” or spontaneous seizures, indicating the development of acquired epilepsy), or both. The development of acute and delayed seizures may have shared as well as unique pathogenic mechanisms and prognostic implications. Based on an extensive review of the literature, we present an overview of viruses that are associated with early and late seizures in humans. We then describe potential pathophysiologic mechanisms underlying ictogenesis and epileptogenesis, including routes of neuroinvasion, viral control and clearance, systemic inflammation, alterations of the blood-brain barrier, neuroinflammation, and inflammation-induced molecular reorganization of synapses and neural circuits. We provide clinical and animal model findings to highlight commonalities and differences in these processes across various neurotropic or neuropathogenic viruses, including herpesviruses, SARS-CoV-2, flaviviruses, and picornaviruses. In addition, we extensively review the literature regarding Theiler’s murine encephalomyelitis virus (TMEV). This picornavirus, although not pathogenic for humans, is possibly the best-characterized model for understanding the molecular mechanisms that drive seizures, epilepsy, and hippocampal damage during viral infection. An enhanced understanding of these mechanisms derived from the TMEV model may lead to novel therapeutic interventions that interfere with ictogenesis and epileptogenesis, even within non-infectious contexts.

## Introduction

Seizures are common presenting symptoms of viral infections of the central nervous system (CNS), and can occur during the acute phase of infection (“early” or acute symptomatic seizures or status epilepticus), after recovery (“late” or spontaneous seizures; indicating the development of acquired epilepsy), or both (Misra et al., 2008; Vezzani et al., 2016). These two types of epileptic seizures have different underlying mechanisms and prognostic implications (Löscher et al., 2015). Over 100 different neurotropic viruses cause encephalitis (i.e., inflammation of the brain parenchyma) in humans, and of these, several play a significant role in the development of seizures and epilepsy (Table 1). Some types of viral encephalitis occur sporadically in worldwide distribution, while others have restricted geographic ranges, often related to specific viral vectors and hosts (Theodore, 2014). The incidence both of acute symptomatic seizures and subsequent epilepsy varies with the specific type of viral encephalitis (mainly dependent on the affected brain regions), the patient’s age, delays in starting treatment, and possibly the degree of cortical inflammation (Misra et al., 2008; Michael and Solomon, 2012; Theodore, 2014). In contrast to encephalitis (or encephalomyelitis), viral infection confined to the meninges rarely causes seizures and does not increase the risk for later epilepsy (Theodore, 2014).

**TABLE 1:** Common viruses associated with seizures and epilepsy in humans.

Genus or family	Species	Neuro-tropic/neuro-pathogenic	Cause of acute viral encephalitis or encepha-lomyelitis	Type of seizures		Comments	References
				Early (insult-associated; provoked)	Late (spontaneous; i.e., epilepsy)		
Flaviviridae +ssRNA	West Nile virus (WNV)	+	+	+	+	Endemic in temperate and tropical regions throughout the world, causing yearly outbreaks of encephalitis, with a mortality rate of 5–10%. Severe neurological illness in less than 1%.	Briese et al., 2000; DellaBadia et al., 2004; Getts et al., 2008; Suen et al., 2014
	Japanese encephalitis virus (JEV)	+	+	+	+	Given its broad geographic distribution, JEV is probably the most common cause of arbovirus encephalitis. Early seizures are reported in 50–80% of cases; epilepsy is less common.	Misra et al., 2008; Singhi, 2011; Theodore, 2014; Chen et al., 2021
	St. Louis encephalitis virus (SLEV)	+	+	+	?	One of the most important arbovirus infections in North America. It accounts for ∼35–60% of aseptic meningitis in all symptomatic cases in children.	Getts et al., 2008; Misra et al., 2008; Singhi, 2011; Abdullahi et al., 2020
	Dengue virus (DENV)	+	+	+	?	Endemic in more than 100 countries.	Getts et al., 2008; Abdullahi et al., 2020; Zhang et al., 2020
	Zika virus (ZIKV)	+	(+)	+	+	As with other flaviviruses, DENV is a mosquito-transmitted virus that has caused outbreaks across the Americas. Mother-to-child transmission occurs through the placenta. First flavivirus associated with congenital defects, including microcephaly. Epileptic seizures are among the main neurological outcomes of congenital Zika syndrome.	Getts et al., 2008; Zhang et al., 2020
	Tick-borne encephalitis virus (TBEV)	+	+	+	+	Causes long-term neurological sequelae in up to 60% of symptomatic patients.	Getts et al., 2008; Theodore, 2014; Zelano and Westman, 2020
Retroviridae +ssRNA	Human immunodeficiency virus (HIV)	+	+	+	+	2–20 percent of HIV-positive patients present with seizures. Epilepsy develops in about 4%.	Kellinghaus et al., 2008; Singhi, 2011; Theodore, 2014; Atluri et al., 2015; Zhang et al., 2020
Picornaviridae +ssRNA	Enterovirus (EV)	+	+	+	(+)	Poliovirus is the prototypical neurotropic enterovirus. Others, such as EV71, EV70, and EV68 show evidence of neurotropism and neuropathogenesis. Seizures are a manifestation of EV infections, especially in children.	Wakamoto et al., 2000; Hosoya et al., 2001; Li et al., 2002; Mistchenko et al., 2006; Chen et al., 2010; Lewthwaite et al., 2010; Ooi et al., 2010; Choi et al., 2011; Kaida et al., 2011; Singhi, 2011; Yang et al., 2011; Demir et al., 2012; Lee et al., 2014; Huang and Shih, 2015; Ong and Wong, 2015; de Graaf et al., 2016; Lee et al., 2016; Teoh et al., 2016; Too et al., 2016; Thong et al., 2017; Yu et al., 2017; Jones et al., 2018; Xing et al., 2018; Han et al., 2019; Pascual-Goni et al., 2019; Sberna et al., 2020; Lim et al., 2021
	Coxsackievirus (CV)	+	+	+	?	Seizures occur in patients infected with CVA6, CVA10, and CVB5. Evidence indicates neurotropism in CVB3, CVB4, and others.	Modlin et al., 1991; Härtel et al., 2002; Jmii et al., 2020; Sberna et al., 2020;
	Rhinovirus (RV)	?	+	+	?	Very common cause of viral infection but extremely rare cause of encephalitis.	Hazama et al., 2019; Soma et al., 2021
	Echovirus (ECHOV)	?	+	+	+	ECHOV are a common cause of aseptic meningitis in children, but ECHOV3 and ECHOV6 are associated with encephalitis and seizures.	Wilfert et al., 1977; Lee et al., 2010; Sberna et al., 2020
	Parechovirus (PeV)	+	+	+	+	PeVs are common in very young children, especially PeV3.	Getts et al., 2008; Verboon-Maciolek et al., 2008; Cabrerizo et al., 2015; Wiley, 2020
Coronaviridae +ssRNA	Severe acute respiratory syndrome coronavirus type 1 (SARS-CoV-1)	+	(+)	+	?	In patients with neurological manifestations, seizures are a common symptom.	Asadi-Pooya, 2020
	Severe acute respiratory syndrome coronavirus type 2 (SARS-CoV-2)	+	(+)	+	(+)	Ongoing global *pandemic.* Too early for long-term studies on potential development of epilepsy, but more than 35% of COVID-19 patients develop neurological symptoms. Seizures are observed in 1–2% of COVID-19 patients.	Asadi-Pooya et al., 2021; Mohan et al., 2021; Pröbstel and Schirmer, 2021; Tavkar et al., 2021; Milano et al., 2022
	Middle East respiratory syndrome coronavirus (MERS-CoV)	+	(+)	?	?	In one study of 70 patients with MERS-CoV infection, six people (8.6%) had seizures	Asadi-Pooya, 2020
Togaviridae +ssRNA	Equine Encephalitis viruses (EEV)	+	+	+	+	Important family members include the alphaviruses Eastern EEV, Western EEV, and Venezuelan EEV.	Carrera et al., 2013; Salimi et al., 2020
Orthomyxoviri-dae −ssRNA	Influenza viruses (IAV)	(+)	+	+	(+)	Each year, about 500 million people are infected worldwide by IAV type A and B, of which about 500,000 die. Most influenza strains are non-neurotropic. Seizures are the most commonly reported neurologic complication, including febrile seizures in children. Influenza accompanied by complications are associated with a slightly increased epilepsy risk.	Fauci, 2006; Atluri et al., 2015; Wilson et al., 2015; Carman et al., 2019; Han et al., 2019; McEntire et al., 2021
Paramyxo-viridae −ssRNA	Mumps virus (MuV)	+	+	+	?	Highly neurotropic. May cause acute encephalopathy in children with high incidence.	Koyuncu et al., 2013; Zhang et al., 2020
	Measles virus (MeV)	+	+	+	+	Unlike MuV, MeV infection spreads to the CNS in only ∼0.1% of cases, but can cause several types of devastating neurological diseases, such as subacute sclerosing panencephalitis, which leads to epilepsy in 45% of patients.	Koyuncu et al., 2013; Zhang et al., 2020
	Nipah virus	?	+	+	+	Seizures are reported in about one-quarter of affected individuals.	Singhi, 2011; Singh et al., 2019
Rhabdoviridae −ssRNA	Rabies virus (RABV)	+	+	+	?	Few long-term studies because of high mortality.	Getts et al., 2008; Theodore, 2014
Peribunya-viridae −ssRNA	La Crosse virus (LACV)	+	+	+	+	LACV is a leading cause of pediatric arboviral encephalitis in the US. Other relevant family members include California Encephalitis virus.	de los Reyes et al., 2008; Teleron et al., 2016; Evans et al., 2019; Ding et al., 2020; Ojha et al., 2021
Arenaviridae ±ssRNA	Lassa Fever virus (LASV)	?	+	+	?	Viral hemorrhagic disease endemic to West Africa.	Cummins et al., 1992; Chika-Igwenyi et al., 2021
	Lymphocytic Choriomeningitis virus (LCMV)	+	+	+	(+)	Carried by common house mouse. Causes aseptic meningitis in immunocompetent subjects.	Wright et al., 1997; Fischer et al., 2006; Kang and McGavern, 2008; Vilibic-Cavlek et al., 2021
Herpesviridae dsDNA	Herpes simplex virus type 1 (HSV-1)	+	+	+	+	Most common cause of sporadic encephalitis. Presents with seizures in 40–70% of individuals. Late unprovoked seizures (epilepsy) occur in 40–65% of adult patients after an episode of herpes simplex encephalitis (HSE).	Libbey and Fujinami, 2011; Singhi, 2011; Sellner and Trinka, 2012; Theodore, 2014
	Herpes simplex virus type 2 (HSV-2)	+	+	+	+	Less common cause of sporadic encephalitis (more common in neonates); accounts for only 2–6% of HSE.	Sellner and Trinka, 2012; Theodore, 2014
	Cytomegalovirus (CMV)	+	+	+	+	Congenital CMV infection is the most common intrauterine infection, affecting 0.2–2.2% of all newborns. In one clinical study, 7 out of 19 infants developed epilepsy.	Suzuki et al., 2008; Atluri et al., 2015; Lei et al., 2015
	Varicella zoster virus [Human herpes virus 3 (HHV-3)]	+	+	+	?	Rarely neuroinvasive for the CNS.	Carey et al., 2017; Zhang et al., 2020
	Human herpes virus 6 (HHV-6)	+	+	+	+	Often involved in febrile seizures (which are a risk for the development of temporal lobe epilepsy).	Millichap and Millichap, 2006; Libbey and Fujinami, 2011; Epstein et al., 2012
	Human herpes virus 7 (HHV-7)	+	+	+	+	Less often involved in febrile seizures than HHV-6.	Millichap and Millichap, 2006; Epstein et al., 2012; Li et al., 2014
	Epstein Barr virus	+	+	+	?	Less often involved in febrile seizures than HHV-6.	Getts et al., 2008; Millichap, 2015; Bartolini et al., 2018; Zhang et al., 2020

*The data shown are based on extensive literature research, using Pubmed and Google Scholar.*

*Evidence of seizures or epilepsy is indicated by “+”; preliminary or anecdotal evidence by “(+)”; and lack of published data by “?”.*

*Positive-sense single-stranded RNA viruses are indicated by +ssRNA, negative-sense single-stranded RNA viruses by −ssRNA, and double-stranded DNA viruses by dsDNA.*

## Mechanisms of Virus Invasion Into the Central Nervous System

As will be discussed later in this review, the mechanisms by which neurotropic viruses enter the brain may by themselves lead to ictogenic and epileptogenic brain alterations, particularly when the mechanism of invasion involves damaging the blood-brain barrier (BBB). Most acute and persistent viral infections begin in the periphery and only rarely spread into the CNS, because the CNS is protected from most virus infections by effective immune responses and specific barriers, such as the BBB or the blood-cerebrospinal fluid (CSF) barrier (Koyuncu et al., 2013; Löscher and Friedman, 2020). However, neurotropic viruses may enter the brain through multiple routes (Figure 2 and Table 2). Most commonly, they spread hematogenously, i.e., across the BBB, but they can also invade the brain *via* the olfactory nerves in the nasal mucosa, through the choroid plexus into the CSF, or *via trans-*synaptic retrograde transport following infection of peripheral nerves (Nath and Johnson, 2021).

**TABLE 2:** Mechanisms of neuroinvasion by neurotropic viruses.

Viruses	Hematogenous transmission route		Axonal transmission route	
	Blood-brain barrier	Blood-CSF barrier	Nasal/olfactory route	Axonal transport from peripheral neurons
West Nile	+ (transcellular, paracellular, Trojan horse, BCEC infection)		+	+
Japanese encephalitis	+ (transcellular)		+	+
St. Louis encephalitis	+			
Dengue	+			
Zika	+ (transcellular)			
Tick-borne	+			
Influenza			+	+
Mumps	+	+		
Measles	+			
Nipah	+		+?	
Rabies		+		+ (neuromuscular junction)
HIV	+ (Trojan horse)			
Enteroviruses	+ (poliovirus [BCEC infection])			+ [e.g., poliovirus (neuromuscular junction)]
SARS-CoV-2	+	+	+	
HSV-1			+	+
HSV-2			+	+
CMV	+ (BCEC infection)			
HHV-3 (VZV)			+	+
HHV-6			+	
HHV-7			+	+
EPV	+ (BCEC infection)			

*Note that, at least in part, these data are from preclinical models.*

*For details, see Koyuncu et al. (2013), Cain et al. (2019), and Nath and Johnson (2021).*

*Abbreviations: BCEC, brain capillary endothelial cell; CSF, cerebrospinal fluid; HIV, human immunodeficiency virus; HSV, herpes simplex virus; CMV, cytomegalovirus; HHH, human herpesvirus; EPV, Epstein-Barr virus.*

The BBB is a dynamic, highly selective barrier primarily formed by brain microvascular endothelial cells (BMECs) connected by tight junctions that separate the circulating blood from the brain parenchyma (Löscher and Friedman, 2020). The tight junctions between the BMECs limit the paracellular flux of hydrophilic and macro-molecules as well as the entry of cells across the BBB, while nutrients such as glucose and amino acids enter the brain *via* specific membrane transporters. As shown in Figure 2, in addition to endothelial cells, the BBB is composed of the capillary basal or basement membrane, pericytes embedded within the basal membrane, and the glia limitans, formed by astrocytic end-feet processes that surround the endothelial cells and add to the barrier properties (Löscher and Friedman, 2020). As summarized in Figure 2 and Table 2, viruses can use diverse routes of neuroinvasion that also dictate which brain regions are affected by the virus.

Although the BBB protects the brain from pathogens, viruses can penetrate the barrier by several means. One way is through direct infection of the brain endothelium resulting in transcellular transport into the CNS (Table 2 and Figure 2). Examples of viruses thought to enter the CNS through this route include West Nile virus (WNV) and poliovirus (Coyne et al., 2007; Verma et al., 2009). Pathogens also may cross the BBB paracellularly *via* disruption of the tight junctions or by damaging BMECs (Cain et al., 2019). Strategies used by neurotropic pathogens in this regard include induced secretion of tight junction-disrupting proteases and toxins, hijacking of host inflammatory and immune responses, and lytic damage of BMECs. Further, it is thought that viruses may enter the brain at regions of heightened permeability (Nath and Johnson, 2021). The BBB is heterogeneous throughout the CNS, and some regions, such as the circumventricular organs, are more permeable than others due to the absence of tight junctions (Löscher and Friedman, 2020). Alternatively, viruses may penetrate the BBB and enter the brain parenchyma through the trafficking of infected leukocytes, often termed “The Trojan Horse” pathway (Outram et al., 1975; Williams and Blakemore, 1990). Phagocytic leukocytes contribute to the clearance of viral, bacterial, and parasitic infections. However, after the internalization of the virus or direct infection of the leukocytes, pathogens may exploit the migratory capabilities of these cells to cross the BBB and lead to CNS infection (Figure 2 and Table 2). Other pathogens, e.g., mumps and rabies viruses, use hematogenous routes to gain access to the CSF compartment (Table 2).

Another mechanism of virus invasion into the CNS is *via* the olfactory system (Figure 2), which provides a unique and directly accessible portal of entry to the CNS from the periphery (Koyuncu et al., 2013). As shown in Table 2, several viruses may infect neurons in the nasal olfactory epithelium. Spread to the CNS occurs *via* anterograde axonal transport along the olfactory nerve into the brain (Figure 2). The olfactory epithelium is well protected from most common infections by mucus and the presence of several pathogen recognition receptor systems (Kalinke et al., 2011). However, there is evidence that pathogens such as herpes simplex virus type-1 (HSV-1), influenza A virus (IAV), parainfluenza viruses, rabies virus, and, more recently, SARS-CoV-2 (severe acute respiratory syndrome coronavirus type *2*) can enter the CNS through the olfactory route (Table 2). Following CNS entry *via* the olfactory system, the virus may spread to other parts of the brain, e.g., using axonal transport *via* the lateral olfactory tract to the hippocampus, which often acts as a focus in the development of epilepsy and cognitive impairment following virus infections (Vezzani et al., 2016).

Viruses such as the herpes viruses and rabies virus infect peripheral neurons (Table 2), leading to anterograde or retrograde transport of virions or viral ribonucleoprotein complexes within axons into the CNS, followed by *trans-*synaptic transport and infection of new neurons (Vezzani et al., 2016).

Another possible mechanism of viral invasion is just the entry of viral proteins and not the entire virus into the CNS. For instance, Rhea et al. (2021) reported that the S1 subunit of the spike protein of SARS-CoV-2 crosses the mouse BBB by adsorptive transcytosis and that murine angiotensin-converting enzyme 2 (ACE2) is involved in brain and lung uptake, but not in kidney, liver or spleen uptake. In a subsequent *in vitro* study, the S1 protein was shown to cross the human brain endothelial cell barrier effectively (Petrovszki et al., 2022).

## Central Nervous System-Specific Consequences of Viral Infection

Central nervous system viral infections are a major cause of death and disability globally (Manglani and McGavern, 2018). The spatial distribution of CNS infection and localization of the consequent immune response results in meningitis (inflammation restricted to the meninges), meningoencephalitis (inflammation of the meninges and brain parenchyma), myelitis (inflammation of the spinal cord), encephalitis (inflammation of the brain parenchyma), or encephalomyelitis (inflammation of the brain and spinal cord). The manifestations of CNS viral infection include fever, altered mental state, neurocognitive impairment, seizures, brain damage, stroke, and death. For many viruses, a robust innate immune response is readily elicited at CNS barriers, including the meninges, the perivascular space, and the ventricular system, which prevents further spread into the subjacent parenchyma (Vincenti and Merkler, 2021). At these CNS barriers, specialized macrophage populations, including dural, leptomeningeal, perivascular, and choroid plexus macrophages, are collectively referred to as CNS-associated macrophages (CAMs) (Kierdorf et al., 2019). Early pathogen detection by CAMs and CNS-resident microglia triggers a disease-associated signature and the release of pro-inflammatory cytokines and chemoattractants (Vincenti and Merkler, 2021). CAMs thereby initiate an inflammatory response that recruits other immune cells, including neutrophils and monocytes. While these innate immune response mechanisms do not directly clear the virus, *per se*, they are vital for the initiation of cytokine-mediated antiviral programs and the subsequent recruitment of adaptive antiviral T cells. Ultimately, the control and clearance of most CNS viral infections depend on the adaptive immune system, including both newly trained antiviral cytotoxic T cells and re-expanded populations of memory lymphocyte subsets (Libbey and Fujinami, 2014). The latter surveil the CNS to rapidly detect invading or re-activating viruses and provide immediate responses toward previously encountered antigens (Vincenti and Merkler, 2021).

If a virus invades the CNS as described above, innate immune responses are mainly coordinated by microglia, i.e., the resident macrophages and primary innate immune cells of the CNS (Chen et al., 2019), and by astrocytes (Klein et al., 2019). Indeed, once thought to be immune-privileged, the CNS is now known to be immune-competent, dynamic, and in direct contact with the peripheral immune system (Manglani and McGavern, 2018). However, the specific role of microglia and other CNS resident cells in this process and their interactions with CNS infiltrating immune cells, such as blood-borne monocytes and T cells, are only incompletely understood. At least in part, this is due to the problems of differentiating invading monocytes from activated microglia in the brain and the lack of selective tools to manipulate these two types of myeloid cells (Greter et al., 2015; Butovsky and Weiner, 2018; Spiteri et al., 2022). Because of the BBB, peripheral monocytes are not found in the CNS parenchyma unless there is overt damage to the barrier or unless pathogen-induced chemokine responses in the brain parenchyma are sufficient to drive monocyte infiltration across the barrier. Iba-1 (ionized calcium-binding adaptor molecule-1) is widely employed as an immunohistochemical marker for both ramified and activated microglia; however, Iba-1 does not discriminate between microglia and peripheral monocytes that have infiltrated the brain (Jeong et al., 2013). Flow cytometry using the expression of cell surface markers such as CD45 and CD11b is widely used to differentiate microglia from CNS invading monocytes (Prinz et al., 2011; Butovsky and Weiner, 2018); however, during neuroinflammation microglia upregulate CD45 expression and may therefore become indistinguishable from monocytes (Yamasaki et al., 2014; Greter et al., 2015; Käufer et al., 2018a). Recent evidence suggests that surface expression of Ly6C/G molecules may adequately distinguish monocytes from microglia (Howe et al., 2022), though as monocytes differentiate into tissue macrophages they likely become, once again, indistinguishable from resident microglia. Adaptive inflammation-associated changes may also affect the specificity of more recent microglia markers such as TMEM119, further blurring the distinction between microglia and infiltrating monocytes (Bennett et al., 2016; Butovsky and Weiner, 2018). Finally, recent single-cell analyses have shown that microglia exhibit a much higher spatial, temporal, and functional diversity than previously thought (Masuda et al., 2020; Sankowski et al., 2021).

In several viral brain infections, activated microglia appear to be involved in both the inhibition of viral replication and in the induction of neurotoxicity, indicating the dual nature of microglia: they contribute to the defense of the CNS but also bear responsibility for CNS damage (Rock et al., 2004; Chhatbar and Prinz, 2021; Figure 3). Microglial phenotypes were, in the past, characterized by the presence of particular cell surface molecules and the expression of specific sets of cytokines and were classified as either M1-like (exhibiting pro-inflammatory signaling and neurotoxicity) or M2-like (participating in the resolution of inflammation) (Butovsky and Weiner, 2018). However, with the help of newly developed technologies, including single-cell RNA-sequencing, quantitative proteomics, and epigenetic studies, it is now clear that this simplistic view of microglial phenotypes does not adequately describe the complex physiology and pathophysiology of microglial cells (Masuda et al., 2020; Sankowski et al., 2021; Waltl and Kalinke, 2022).

Microglia expresses various pattern recognition receptors (PRRs) that recognize viral signatures called pathogen-associated molecular patterns (PAMPs) (Bachiller et al., 2018; Gern et al., 2021). Upon stimulation by PAMPs, microglia release several pro- and anti-inflammatory cytokines such as monocyte chemoattractant protein 1 (MCP1 aka CCL2), interleukin (IL)-1β, type I interferon (IFN), IFNγ, and tumor necrosis factor-α (TNF-α) (O’Shea et al., 2013). This microglial response likely recruits inflammatory monocytes during the acute phase and contributes to CNS recruitment of antiviral CD8^+^ T cells throughout infection. Recruitment of both innate and adaptive immune cells is necessary for effective control of infection, with the innate response limiting viral replication and the adaptive response clearing the virus *via* both cytolytic and non-cytolytic mechanisms (Griffin, 2010; Libbey and Fujinami, 2014). However, as with the dual role of microglia, infiltrating monocytes contribute to neurotoxicity, synaptic dysregulation, and ictogenesis (Howe et al., 2012a,b; Cusick et al., 2013; Varvel et al., 2016; Cusick et al., 2017; Howe et al., 2017; Käufer et al., 2018a; Figure 3).

Recovery from infection requires non-cytolytic clearance of the virus from the CNS to avoid further damage to tissue (Griffin and Metcalf, 2011). B cell production of antiviral antibodies (Bartlett and Griffin, 2020), T cell production of IFN-γ (Milora and Rall, 2019), and other immune responses within the infected nervous system are important for non-cytolytic clearance of infectious virus and viral RNA and also for prevention of viral reactivation and recrudescence (Manglani and McGavern, 2018). Microglia and other neural cells exert direct antiviral effects by producing type I interferons that consequently induce autocrine and paracrine expression of IFN-stimulated genes (ISGs), resulting in viral control and hardening of neural cell susceptibility to further infection (Chen et al., 2019). These signals also induce MHC class I expression and facilitate the presentation of viral peptides that are recognized by antiviral T cells. Infiltrating lymphocytes and natural killer cells, recruited by the same processes that induce type I interferons, produce IFN-γ which drives intracellular processes that block viral replication and enhance the destruction of viral material *via* autophagic and oxidative mechanisms (Lee and Ashkar, 2018). However, despite this symphony of antiviral responses, some pathogens persist in the CNS (Griffin and Metcalf, 2011; Nath and Johnson, 2021), contributing to ongoing tissue damage and neuroinflammatory processes that exacerbate the consequences of infection. Restricted viral replication within the context of persistent infection in the absence of sterilizing immunity results in chronic neuroinflammation (Nath and Johnson, 2021). Viral mechanisms that contribute to persistence include the route of viral entry into the CNS, viral immune evasion strategies, and viral spread to permissible cells (Nath and Johnson, 2021). In parallel, host genetics contribute significantly to viral clearance versus persistence, as exemplified by TMEV infection in SJL versus B6 mice (Howe et al., 2012b; Gerhauser et al., 2019).

Viruses may also enter a latent state within the CNS, marked by the continued presence of viral genomic material but limited gene expression and no replication. A crucial component of such cryptic infection is the reversion to the active expression of the complete viral genome and resurgent production of infectious virions. Herpesviruses such as Epstein-Barr virus (EBV) are canonical latent infectious agents (Speck and Ganem, 2010), and human herpesvirus (HHV)-6, a nearly ubiquitous pathogen in children, establishes latency in the CNS (Dunn et al., 2020). Later reactivation of HHV-6 may drive limbic encephalitis, and, as described below, induce seizures and temporal lobe epilepsy (TLE). Overall, the detection of persistent or latent viruses in the CNS is severely hampered by inaccessibility and the field still has much to learn about the influence of such infections on the development of later-life neurological disorders, ranging from Alzheimer’s disease and multiple sclerosis (MS) to epilepsy.

## Seizures and Epilepsy Associated With Viral Infections of the Central Nervous System

As shown in Figure 4, by definition, “early” or acute symptomatic seizures are seizures that occur during the initial phase (typically the first week) of CNS infection, whereas “late” or unprovoked (spontaneous) recurrent seizures develop in surviving patients after a latent period of weeks, months, or years following the acute phase (Löscher et al., 2015). In more general terms, acute symptomatic seizures occur in a close temporal relationship with the initial infection and typically subside once the acute insult is over, usually without recurrence (Singhi, 2011). Early seizures are not a prerequisite for late seizures but increase the risk of spontaneous, unprovoked seizures (i.e., epilepsy), presumably because early seizures are an indicator of injury that leads to maladaptive changes in neural circuitry (Klein et al., 2018).

In addition to the dysregulation of synapses incurred by the electrophysiological influence of an early seizure associated with CNS viral infection, the infection-associated inflammatory response elicited in resident microglia and generated by infiltrating leukocytes also confers maladaptive synaptic changes that lead to persistent hyperexcitability. Such changes include morphological alteration of synaptic spine structure (Tomasoni et al., 2017), alterations in the balance of inhibitory and excitatory neurons and synaptic channels (Habbas et al., 2015), and transcriptional reprogramming that alters neuronal excitability (Buffolo et al., 2021).

It is estimated that half of all patients with encephalitis experience acute symptomatic seizures, and approximately 4% develop status epilepticus, a medical emergency in which a patient has a seizure lasting longer than 5 min or has multiple discrete seizures between which consciousness is not fully recovered. An episode of status epilepticus, especially one lasting 30 min or more, greatly increases the risk of developing epilepsy (Barnard and Wirrell, 1999). Epilepsy exists when someone has an unprovoked seizure and their brain “demonstrates a pathologic and enduring tendency to have recurrent seizures” (Fisher et al., 2014). More specifically, in survivors of viral infections, epilepsy is diagnosed when an individual has: (1) at least two unprovoked or reflex seizures > 24 h apart, (2) one unprovoked or reflex seizure and a probability of having another seizure similar to the general recurrence risk after two unprovoked seizures (≥60%) over the next 10 years, or (3) an epilepsy syndrome (Fisher et al., 2014).

Importantly, early and late seizures may look very similar, both behaviorally and by EEG (Löscher et al., 2015). Thus, determining whether a patient or group of patients developed epilepsy after viral infection is not trivial, but necessitates a thorough review of symptoms and medical history and detailed diagnostic testing, including high-resolution EEG, to adequately diagnose epilepsy and determine the cause of seizures. This explains why it is often not yet clear, particularly for infections occurring in the developing world, whether a virus infection causes epilepsy or only early seizures. For the current review, we performed an extensive literature search, using Pubmed and Google Scholar, to find studies that unequivocally identified epilepsy as an outcome in patients infected with a variety of neurotropic and neuropathogenic viruses. The outcome of this search is shown in Table 1, demonstrating that many more viruses than thought before can lead to unprovoked seizures and epilepsy.

As shown in Table 1, a variety of different RNA and DNA viruses have been reported to cause acute symptomatic seizures and subsequent epilepsy. Among the viruses shown in Table 1, the high prevalence and spread of arthropod-borne viruses (arboviruses) make them an important cause of viral encephalitis and associated seizures in humans, with between 10 and 35% of patients infected with these viruses displaying some form of seizure (Getts et al., 2008; Singhi, 2011; Zheng et al., 2020). Among the various arboviruses, flaviviridae such as WNV, Japanese encephalitis virus (JEV), Zika virus (ZIKV), and tick-borne encephalitis virus (TBEV) have been reported to induce both early and, in survivors, late (spontaneous) seizures (Table 1).

JEV is the single largest cause of acute epidemic encephalitis worldwide (Singhi, 2011). Acute symptomatic seizures are reported in 50–80% of cases and are much more frequent in children than in adults. The seizures are generalized or focal with secondary generalization, single or multiple, and may present as status epilepticus. Late-onset epilepsy is less common in JEV (Singhi, 2011; Chen et al., 2021). Concerning congenital ZIKV syndrome, recent reports show that epileptic seizures are among the main neurological outcomes of this syndrome (Table 1).

Among the sporadic viral encephalitides, herpes simplex encephalitis (HSE) is perhaps most frequently associated with epilepsy, which may often be severe (Misra et al., 2008). Seizures may be the presenting feature in 40–70% of patients during acute infection and the frequency of epilepsy in survivors may be 40–60% (Theodore, 2014). The propensity to cause seizures is probably related to viral spread along olfactory pathways to limbic structures including the temporal lobe, insula, and cingulate cortex. Other potentially neurotropic viruses, such as measles, varicella, mumps, IAV, and enteroviruses may cause seizures depending on the area of the brain involved (Misra et al., 2008).

COVID-19 (coronavirus disease 2019), the global pandemic caused by SARS-CoV-2, is considered to be primarily a respiratory disease, but SARS-CoV-2 infection affects multiple organ systems including the CNS (Mishra and Banerjea, 2020). Numerous reports have described seizures in people with COVID-19 (Asadi-Pooya, 2020; Asadi-Pooya et al., 2021; Doyle, 2021; Nolen et al., 2022), though it is unclear how many of these seizures arise as a complication of systemic inflammation, peripheral organ damage, and vascular injury versus more direct infection-related effects on the CNS. It is also too early to determine whether COVID-19 is associated with epilepsy, although several anecdotal reports suggest *de novo* epilepsy in these patients (Elgamasy et al., 2020; Nikbakht et al., 2020). In children, seizures may be the main presenting manifestation of acute SARS-CoV-2 infection (Kurd et al., 2021). In the as-yet largest study on neurological manifestations of COVID-19, seizures were observed in 74 of 4491 patients (1.6%), which was the third most common neurological manifestation after encephalopathy and stroke (Frontera et al., 2021). No patient had meningitis/encephalitis or myelopathy/myelitis that was conclusively related to direct SARS-CoV-2 invasion of the CNS. However, these findings do not eliminate the possibility of direct CNS invasion of SARS-CoV-2. Indeed, more recently, olfactory transmucosal SARS-CoV-2 invasion has been described as a port of CNS entry in individuals with COVID-19 (Meinhardt et al., 2021).

Some viruses, including HHV-6, IAV, adenovirus, and rhinovirus, are associated with febrile seizures, i.e., seizures that are triggered by fever, typically above 38.3°C. These seizures are the most common type of convulsions in infants and young children (Millichap and Millichap, 2006; Epstein et al., 2012; Rudolph et al., 2021). Most febrile seizures last only a few minutes and are not associated with an increased risk of later spontaneous seizures. However, multiple or prolonged febrile seizures, including febrile status epilepticus (fSE), are a risk factor for epilepsy (Shinnar, 2003). Of greatest concern is the small group of children with febrile seizures lasting longer than 30 min. In these children, the risk of developing epilepsy is as high as 30–40%, though the condition may not develop until many years later. The prospective FEBSTAT study examines the consequences of fSE and is clarifying the relationship between fSE, hippocampal atrophy, hippocampal sclerosis, and the development of subsequent TLE and cognitive impairment (Hesdorffer et al., 2012). As such, this study will be instrumental in determining the role of structural hippocampal alterations as a potential mechanism of TLE. Recent data from the FEBSTAT study suggest that prolonged febrile seizures injure the hippocampus (Shinnar et al., 2012; Lewis et al., 2014; McClelland et al., 2016).

Febrile infection-related epilepsy syndrome (FIRES), a subtype of new-onset refractory status epilepticus (NORSE), is a catastrophic epileptic syndrome that strikes previously healthy children between the age of 2 and early adulthood and has unknown pathogenesis and few treatments (Fox et al., 2017; Sculier et al., 2021; Lattanzi et al., 2022; Nausch et al., 2022). Affected children experience a non-specific illness with fever starting between 2 weeks and 24 h before the onset of prolonged refractory status epilepticus. In a few cases, specific pathogens, including rhinovirus, respiratory syncytial virus, and EBV, were identified in serum or nasopharyngeal aspirates (Venkatesan and Benavides, 2015). However, despite extensive testing, pathogens have not been identified in the CNS, suggesting that a systemic infection induces the CNS dysfunction, potentially by triggering inflammation that is communicated across the BBB, inducing sterile encephalitis (Ravizza et al., 2018; Vezzani et al., 2019). The outcome of FIRES varies with the length of the acute phase and is usually poor, with up to 30% of cases ending in death and 60–100% of survivors developing permanent intellectual disability and drug-resistant epilepsy (Fox et al., 2017; Tan et al., 2021).

The occurrence of febrile seizures and FIRES, as well as the occurrence of seizures in COVID-19 patients, suggests that systemic inflammatory responses to viral infection in the absence of neuroinvasion and true encephalitis may be an important pathogenic mechanism in driving seizures and epilepsy. Fever and high levels of circulating inflammatory cytokines alter BBB permeability (Danielski et al., 2018; Remsik et al., 2021) and may permit the transmission of inflammation into the CNS. These events may also facilitate viral entry into the CNS that otherwise would not occur, resulting in transient neural infection or PAMP-induced PRR signaling that drives microglial activation in the absence of leukocyte infiltration. These mechanisms of infection-associated ictogenesis may explain how viruses that show weak or no neurotropic potential still elicit early seizures that confer heightened risk for later development of epilepsy. Indeed, systemic virus infection-associated indirect neuroinflammation and ictogenesis may be the parallel of sepsis-associated encephalopathy (Gao and Hernandes, 2021).

In addition to viral infections as a trigger for ictogenesis and epileptogenesis, such infections may affect disease progression in patients with existing epilepsy (Vezzani et al., 2016; Tan et al., 2021). In particular, the inflammation associated with viral infections contributes to the progression of the disease (see below).

## Mechanisms of Seizures and Epilepsy Associated With Viral Infections

**FIGURE 1:**
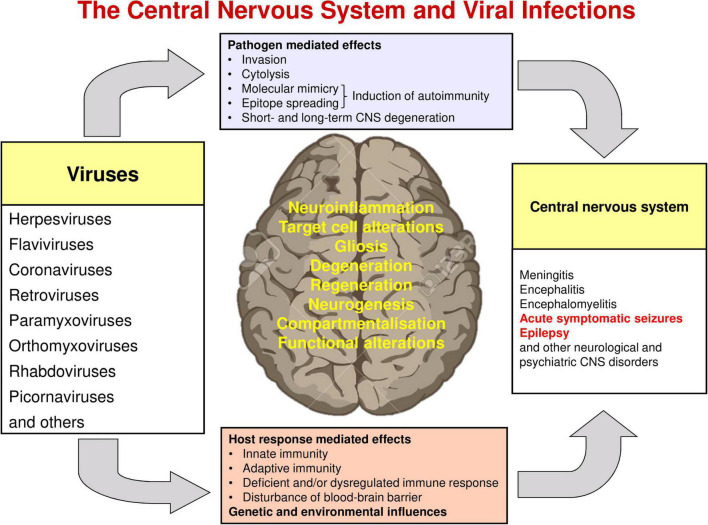
Interactions of viruses and the central nervous system. Modified from Vezzani et al. (2016).

The various molecular, structural, and functional alterations in the CNS that are potentially involved in the generation of seizures and epilepsy associated with viral infections are illustrated in Figures 1, 3–5. The mechanisms underlying the generation of early and late seizures vary with the type and location of infection. In general, early seizures are an acute consequence of virus infection, either directly *via* neuroinvasion and encephalitis or indirectly *via* systemic inflammation and neuroinflammation. In contrast, late seizures arise from the functional and structural alterations that drive epileptogenesis, a multifactorial process that is outlined in Figure 4.

**FIGURE 2:**
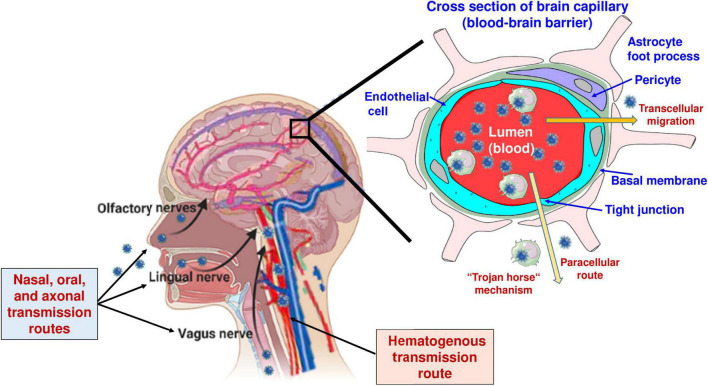
Routes of virus invasion into the brain. In addition to the routes illustrated in the figure, viruses may enter the central nervous system (CNS) *via* the choroid plexus, i.e., the blood-CSF barrier (see Table 2). Modified from Löscher and Potschka (2005), Löscher and Friedman (2020), and Sulzer et al. (2020).

**FIGURE 3:**
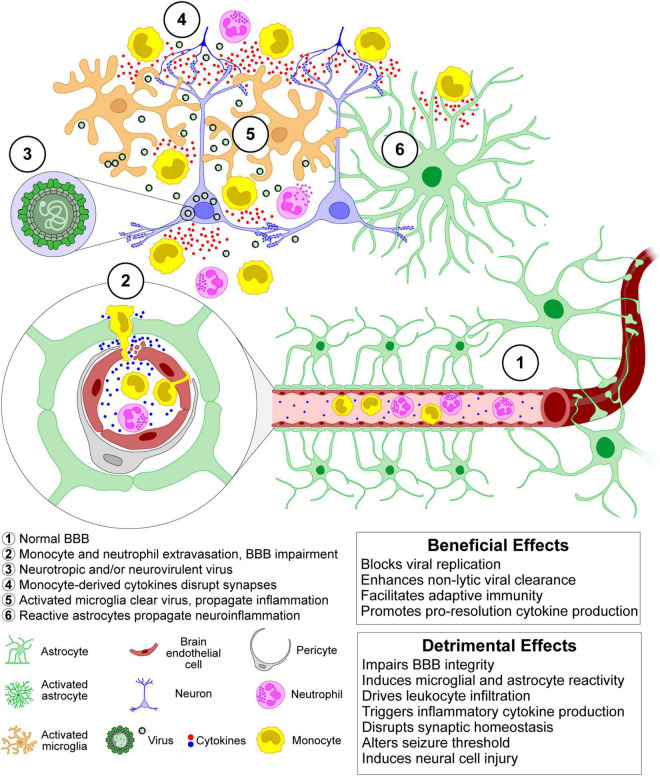
The acute inflammatory and neuroinflammatory responses to CNS viral infection exert both protective and injurious effects. Leukocyte infiltration in response to chemokine production induced by pattern recognition receptor binding to viral components and endogenous alarmins results in potentially pathogenic alterations at the blood-brain barrier and leads to a robust release of cytokines that are critical for enhancing viral control before the development of an adaptive antiviral response. However, these cytokines also disrupt synaptic function and homeostasis, leading to neuronal injury and changes in excitability that confer a pro-ictogenic effect. Ultimately, viral control and clearance from the CNS is a trade-off between exuberant innate immune responses and consequent cellular and circuit damage.

**FIGURE 4:**
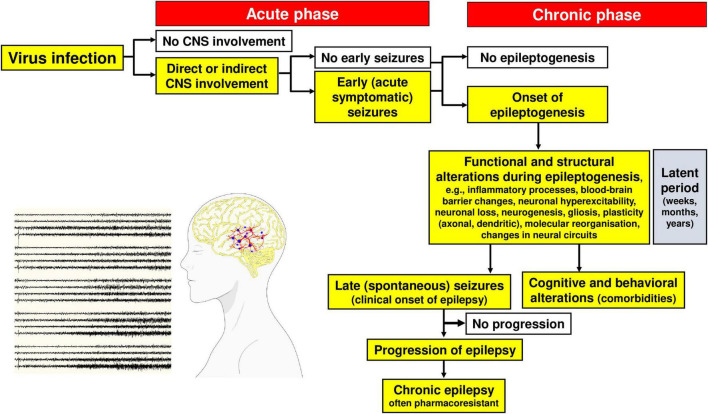
Steps in the development and progression of acquired epilepsy (often temporal lobe epilepsy) as a consequence of viral infections. The term epileptogenesis includes processes that take place before the first spontaneous seizure occurs, which render the brain susceptible to spontaneous recurrent seizures and processes that intensify seizures and make them more refractory to therapy (progression). The concept illustrated in the figure is based on both experimental and clinical data. Adapted from Löscher et al. (2008).

**FIGURE 5:**
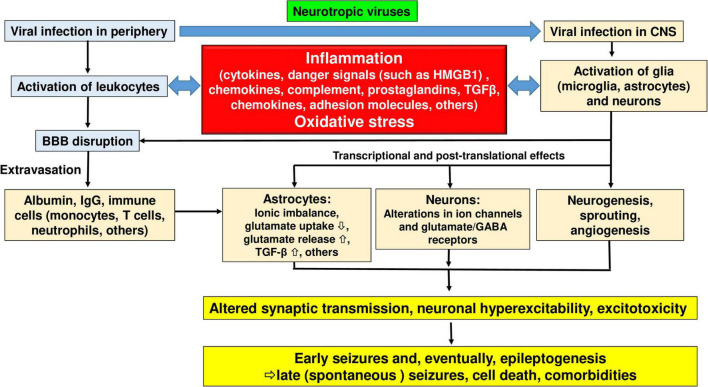
Pathophysiological cascade of events leading from viral infection to inflammation to seizures and epilepsy. See text for details. Modified from Vezzani et al. (2011) and Vezzani et al. (2019).

### Alterations of the Blood-Brain Barrier as a Mechanism of Ictogenesis and Epileptogenesis

Hematogenous transmission of virus to the CNS involving either BMEC infection, damage to the tight junctions, or both, results in changes to BBB integrity that are likely an essential mechanism of subsequent ictogenesis and epileptogenesis (Löscher and Friedman, 2020). One hallmark of a damaged BBB is the extravasation of albumin from the blood to the brain parenchyma (Friedman et al., 2009). In the brain parenchyma, albumin can be taken up or bound to neurons, astrocytes, and microglial cells. In astrocytes, albumin can be taken up *via* transforming growth factor-beta (TGF-β) receptors. This is followed by downregulation of inward rectifying potassium channels (Kir 4.1), water channels (aquaporin 4; AQP4), and glutamate transporters in these astrocytes (Löscher and Friedman, 2020). As a result, the buffering of extracellular potassium and glutamate is reduced, which facilitates N-methyl-D-aspartate (NMDA) receptor-mediated neuronal hyperexcitability and eventually induces epileptiform activity (Löscher and Friedman, 2020). TGF-β receptor signaling is further associated with transcriptional changes involved in inflammation, alterations in extracellular matrix (specifically the perineuronal nets around inhibitory interneurons), excitatory synaptogenesis, and pathological plasticity, all considered important mechanisms that contribute to lowering the seizure threshold during epileptogenesis (Löscher and Friedman, 2020). As a proof-of-concept that albumin extravasation plays a crucial role in the generation of seizures, the angiotensin II type 1 (AT1) receptor antagonist, losartan, which blocks brain TGF-β receptor signaling, was shown to prevent epilepsy in different models of epileptogenesis (Swissa et al., 2019).

### Structural Brain Alterations as a Mechanism of Ictogenesis and Epileptogenesis

For many decades, the limbic system in the temporal lobes, including the hippocampal formation and parahippocampal areas such as the piriform, perirhinal, and entorhinal cortices, have been known to play a crucial role in the development of seizures and epilepsy (Walter, 1969; Meldrum, 1975; Ribak et al., 1992; Engel, 1996; Löscher and Ebert, 1996; Chatzikonstantinou, 2014; Scharfman, 2019). The hippocampus is considered by many to be the generator of TLE, the most common type of epilepsy in adults and a frequent consequence of viral infections (Vezzani et al., 2016). TLE is typically associated with hippocampal sclerosis, a neuropathological condition with severe neuronal cell loss and gliosis in the hippocampus, specifically in the CA1 (Cornu Ammonis area 1) region and subiculum of the hippocampus proper and in the hilus of the dentate gyrus (Blümcke et al., 2002). Hippocampal sclerosis was first described in 1880 by Wilhelm Sommer as an etiological component of epilepsy (Sommer, 1880). In addition to neuron loss, aberrant sprouting of dentate granule cell mossy fibers in mesial TLE is thought to underlie the creation of aberrant circuitry that promotes the generation or spread of spontaneous seizure activity (Sutula and Dudek, 2007; Scharfman, 2019). Surgical removal of the sclerotic hippocampus in drug-resistant patients often improves or even cures TLE (Löscher et al., 2020). Thus, these structural changes in the hippocampal formation provide a mechanism by which viral infections could induce seizures and epilepsy.

As discussed above, some viruses may be more epileptogenic due to their anatomic distribution, as in the case of HSV, with a propensity to affect the temporal lobes, including the hippocampus (Theodore, 2014). HSV causes widespread inflammation, edema, and parenchymal necrosis (Theodore, 2014). Experimental corneal inoculation of HSV-1 in BALB/c mice led to increased CA3 pyramidal cell excitability and aberrant mossy fiber sprouting in the hippocampus as well as clinical seizures (Wu et al., 2003). Remarkably, after initial infection, HSV can establish persistent latent infections in the CNS, acting as a continuous source of HSE recurrence (Zhang et al., 2020).

Concerning the neurotropic virus HHV-6, several studies and a recent meta-analysis suggest a pathogenic role of HHV-6B infection in the development of mesial TLE, especially when associated with hippocampal sclerosis and a history of febrile seizures (Wipfler et al., 2018; Bartolini et al., 2019; Wang and Li, 2021). HHV-6, which is ubiquitous and infects most people when they are children, establishes latent infections in the CNS, especially in the hippocampus and amygdala, and is associated with neurologic diseases, including TLE (Wang and Li, 2021). In a meta-analysis of studies that detected HHV-6 genomic DNA or protein in brain samples from the hippocampus of people with mesial TLE, HHV-6 DNA was detected in 19.6% of all TLE patients compared to 10.3% of all controls (*P* < 0.05) (Wipfler et al., 2018).

Transcriptional analysis of the amygdala in patients with hippocampal sclerosis revealed higher expression of CCL2 and glial fibrillary acidic protein (GFAP) in HHV-6 positive samples and a positive correlation between viral load and protein expression (Kawamura et al., 2015). As described above, CCL2 is a chemokine that participates in the migration and CNS infiltration of monocytes, in which HHV-6 can establish latent infection (Bartolini et al., 2019). Overexpression of GFAP and CCL2 is associated with neuronal loss and gliosis and has been previously described in resected epileptogenic tissue from the hippocampus (Xu et al., 2011). However, the casual relationship and possible pathological role of HHV-6 in TLE are yet to be elucidated. Infections with ZIKV have also been reported to cause alterations in temporal lobe structures such as the hippocampus, leading to memory and behavioral deficits and seizures (Stanelle-Bertram et al., 2018; Büttner et al., 2019; Raper et al., 2020). This will be discussed in more detail below.

### Inflammatory Processes as a Mechanism of Ictogenesis and Epileptogenesis

Upon viral invasion of the CNS, activation of the innate and adaptive immune response is critical to control viral replication and spread (Libbey and Fujinami, 2014; Figure 3). However, an exuberant innate response to the infection may cause considerable acute bystander pathology, while failing to adequately control viral replication which may lead to persistent smoldering inflammation that results in chronic neuropathology (DePaula-Silva et al., 2021; Figure 3). In general, as illustrated in Figure 5, inflammation plays a prominent role in the mechanisms underlying increased neuronal excitability in both early and late seizures associated with virus infection (Vezzani et al., 2016). Furthermore, oxidative stress is thought to contribute to these processes (Figure 5). As shown in Figures 1, 3, 5, initiation of neuroinflammation may either be the result of neuroinvasion, host danger signal response mediated effects or both. As described above, encephalitis is defined as inflammation of parenchymal CNS tissue that occurs in response to viral replication (Vezzani et al., 2016). Once a virus enters the brain parenchyma, inflammation may result from two mechanisms that are not mutually exclusive. First, viruses may directly infect neurons leading to unconstrained neuronal lysis and death and the release of proinflammatory cytokines and cellular products that act as endogenous danger signals (such as ATP or mitochondria-derived DNA *N*-formyl peptides) (Vezzani et al., 2016; Di Virgilio et al., 2020; Das et al., 2021). Second, viral PAMPs may activate PRRs on microglia and astrocytes, leading to cytokine and chemokine production that recruits innate immune effectors that drive immunopathology. These inflammatory responses drive acute injury but are also associated with the formation of a residual pathological state marked by continued BBB dysfunction and injury, neuronal death, and persistent neuronal hyperexcitability, all of which may contribute to ictogenesis and epileptogenesis.

Viruses may also trigger post-infectious encephalitis or encephalomyelitis, even in the absence of neuroinvasion during the initial infection. Such delayed responses are elicited following the development of T cell- and/or antibody-mediated recognition of self epitopes (Vezzani et al., 2016; Popkirov et al., 2017; Joubert and Dalmau, 2019). Molecular mimicry, epitope spreading, and unmasking of autoreactive lymphocytes (see Figure 1) are the primary mechanisms by which infectious agents induce autoimmunity (Powell and Black, 2001; Cusick et al., 2012; Pape et al., 2019; Gupta and Weaver, 2021).

During the acute response to CNS infection, brain resident cells recruit peripheral immune cells to sites of viral infection (Manglani and McGavern, 2018). Among the acute responders, CNS infiltration of monocytes and neutrophils is a hallmark of CNS inflammation, including viral infection (Terry et al., 2012). These cells engage in several potent effector functions including the production and secretion of numerous pro-inflammatory mediators and reactive oxygen species that drive tissue damage (Terry et al., 2012). Monocytes that migrate into the infected brain also differentiate into macrophages, dendritic cells, and, arguably, microglial populations (see below). In addition to invasion of blood-borne immune cells such as monocytes and neutrophils, brain resident innate immune cells, including microglia and astrocytes, also produce proinflammatory cytokines and reactive oxygen species that contribute to inflammation and CNS injury (Figure 5).

It has been proposed that the IL-1 cytokine system may play a pivotal role in the development of fSE and mesial TLE (Dube et al., 2005; Dube et al., 2010). IL-1β is the primary cytokine responsible for mediating febrile responses in humans and it is a powerful proconvulsant implicated in ictogenesis and epileptogenesis (Dube et al., 2010; Vezzani et al., 2016; Vezzani et al., 2019). At least in part, this effect of IL-1 β is related to its suppressive action on inhibitory GABA currents and enhancement of NMDA-mediated neuronal Ca^2+^ influx, resulting in increased glutamatergic excitation (Huang et al., 2011; Mishra et al., 2012; Vezzani and Viviani, 2015). The effects of IL-1β are mediated *via* IL-1 receptor type 1 (IL-1R1), which is enriched in cortical and hippocampal neurons where it co-localizes and physically associates with the NR2B (GluN2B) subunit of the NMDA receptor (Vezzani and Viviani, 2015). IL-1R1 is activated by IL-1β that is released from neurons, glia, brain endothelial cells, and infiltrating monocytes following inflammasome activation (Labzin et al., 2016; Vezzani et al., 2019). Elevation of IL-1β induces robust release of other proinflammatory cytokines, including IL-6 and CXCL8 (Heida and Pittman, 2005; Vezzani et al., 2016). A recent study that examined the association between plasma cytokines and fSE in children, as well as their potential as biomarkers of acute hippocampal injury, found that levels of CXCL8 and epidermal growth factor (EGF) were significantly elevated after fSE in comparison to controls (Gallentine et al., 2017). However, individual cytokine levels were not predictive of MRI changes in the hippocampus.

The nuclear protein high mobility group box 1 (HMGB1), which is released by neurons and macrophages/monocytes in response to exogenous and endogenous inflammatory stimuli and during unconstrained cell death, is thought to play a critical role as a danger signal in virus infection-induced inflammatory responses in the CNS (Wang et al., 2006; Vezzani et al., 2016; Walker et al., 2022). Furthermore, HMGB1 has been implicated in the generation of seizures and epilepsy (Ravizza et al., 2018). As with IL-1β, TNF-α, and IL-6, HMGB1 has pro-ictogenic properties in animal models and affects neuronal function by inducing rapid post-translational changes in glutamate receptor subunit composition and/or phosphorylation (Vezzani et al., 2016). HMGB1 physiologically interacts with nucleosomes, transcription factors, and histones within the nucleus of nearly every cell type but is rapidly translocated to the cytoplasm and released following brain injury and during seizures (Jiang et al., 2020; Murao et al., 2021). Several viruses that cause encephalitis and seizures, including WNV, SARS, TBEV, and IAV, can induce the release of HMGB1 (Wang et al., 2006; Ding et al., 2021). HMGB1 binds to and activates the receptor for advanced glycation end products (RAGE), toll-like receptor 4 (TLR4), and TLR2 (Jiang et al., 2020), inducing signal transduction cascades that drive inflammation. Indeed, activation of IL-1R1 and HMGB1 receptors expressed by microglia and astrocytes orchestrates inflammatory events that result in the release of cytokines and chemokines, induction of the prostaglandin-synthesizing enzyme cyclooxygenase 2 (COX-2), and activation of the complement system, and may thereby subsequently lead to recruitment of leukocytes to the brain (Vezzani et al., 2016).

### Virus-Specific Mechanisms: Human Immunodeficiency Virus

Whereas the processes illustrated in Figure 5 and discussed above would be relevant for all viruses that cause encephalitis and/or sterile inflammation, there are also neuropathophysiological processes and outcomes specific to individual viruses. For instance, the transactivator of transcription (Tat) protein is a major viral protein in HIV that can directly drive neurotoxicity (Atluri et al., 2015). Tat is vital for HIV replication and influences transcription initiation and elongation at the HIV promoter. In addition, however, Tat injures neurons *via* several different mechanisms, including induction of inflammatory cytokines, impairment of mitochondrial function, and activation of ionotropic glutamate receptors (Atluri et al., 2015). Indeed, Haughey et al. (2001) reported that HIV-1 Tat potentiates the excitotoxicity of glutamate by phosphorylating NMDA receptors, a process that is critically involved in neuronal hyperexcitability, seizures, and epileptogenesis (Ghasemi and Schachter, 2011; Hanada, 2020). The effect of prolonged exposure to endogenously produced Tat in the brain was investigated using a transgenic mouse model constitutively expressing the HIV-1 Tat gene (Zucchini et al., 2013). Stimulus-evoked glutamate exocytosis in the hippocampus and cortex of these mice was significantly increased and was associated with increased seizure susceptibility. In addition to the effects associated with the Tat protein, the HIV type 1 envelope glycoprotein gp120 activates macrophages, which release neurotoxins that affect the glutamate system, leading to activation of voltage-dependent calcium channels and modulation of NMDA signals (Potter et al., 2013).

### Virus-Specific Mechanisms: Severe Acute Respiratory Syndrome Coronavirus Type 2

Concerning the SARS-CoV-2 virus, the specific mechanisms by which this virus affects the CNS remain unclear (Pröbstel and Schirmer, 2021). As described above, infection with SARS-CoV-2 may result in psychiatric and neurological symptoms, including seizures; more than 35% of COVID-19 patients develop such symptoms, particularly during severe manifestation of the disease (Tavkar et al., 2021). It is well accepted that the entry of SARS-CoV-2 into a host cell is mediated by ACE2, which functions as an entry receptor (Hoffmann et al., 2020). Membrane-bound ACE2 is a zinc-containing metalloenzyme located on the surface of cells. SARS-CoV-2 downregulates ACE2, with a consequent loss of its catalytic activity (Pacheco-Herrero et al., 2021). Inflammation and thrombosis have been associated with enhanced and unimpeded angiotensin II effects through the ACE2-AT1 receptor axis (Pacheco-Herrero et al., 2021).

In the CNS, ACE2 is expressed in the majority of brain regions (e.g., the amygdala, cortex, frontal cortex, substantia nigra, and hippocampus) but mostly at low levels (Chen et al., 2020). Analysis of human and mouse brains showed that ACE2 is expressed predominantly in neurons but also in non-neuronal cells, including astrocytes, oligodendrocytes, endothelial cells, and pericytes (Tavkar et al., 2021). The expression of ACE2 makes CNS cells susceptible to SARS-CoV-2 infection, provided that the virus enters the brain. As summarized in Table 2 and Figure 2, current evidence points to two plausible mechanisms of brain invasion by SARS-CoV-2: (i) entry into the CNS *via* axonal transport along infected olfactory nerves and then dissemination *via trans-*synaptic transmission to other brain areas (Montalvan et al., 2020; Yachou et al., 2020; Meinhardt et al., 2021); note that as many as 65% of COVID-19 affected individuals reported hyposmia, anosmia, and ageusia, suggesting the possibility of transsynaptic spread not only *via* the olfactory route but also along lingual and glossopharyngeal nerves (Figure 2; Sulzer et al., 2020); (ii) entry into the CNS *via* a hematogenous pathway, either through the infiltration of infected blood cells (usually leukocytes) or through infection of endothelial cells at the BBB. The hematogenous pathway may also involve infection of epithelial cells of the choroid plexus, the building blocks of the blood-CSF barrier (Montalvan et al., 2020; Murta et al., 2020; Vargas et al., 2020; Yachou et al., 2020). Another intriguing mechanism *via* which SARS-CoV-2 may spread is through the vagus nerve from infected lungs (Jarrahi et al., 2020). Using human brain organoids derived from induced pluripotent stem cells as a valuable tool for investigating SARS-CoV-2 neurotropism, it was found that choroid plexus organoids showed a high rate of infection and supported productive viral replication, consistent with the finding that the choroid plexus exhibits high ACE2 expression (Jacob et al., 2020; Pellegrini et al., 2020). Besides epithelial cells of choroid plexus, neurons, astrocytes, and neural progenitor cells in brain organoids are also susceptible to SARS-CoV-2 infection, although the infection rates for these cell types remain under debate (Tavkar et al., 2021). Overall, replication of SARS-CoV-2 in the CNS remains a controversial issue.

Concerning the mechanisms of neurological symptoms such as seizures, many groups argue that the devastating neurological damage caused by SARS-CoV-2 is not a consequence of direct infection of neural cells but rather a result of the severe peripheral hyper-inflammation associated with COVID-19 (Pacheco-Herrero et al., 2021; Tavkar et al., 2021). Among the various consequences of such inflammation, impairment of BBB may be involved in CNS symptoms, as discussed above and illustrated in Figures 3, 5. Furthermore, it has been suggested that endothelial dysfunction in several organs, including the CNS, may be triggered by the interaction between SARS-CoV-2 and ACE2 receptors that are expressed by endothelial cells (Pacheco-Herrero et al., 2021). In patients with COVID-19, magnetic resonance imaging (MRI) detected lesions that are compatible with a cerebral small-vessel disease and with disruption of the BBB (Nassir et al., 2021).

More recently, Wenzel et al. (2021) reported structural changes in cerebral small vessels of patients with COVID-19 and elucidated potential mechanisms underlying the vascular pathology. Both in patients and two animal models of SARS-CoV-2 infection, an increase in string vessels was observed in the brain. These structures represent the endothelial cell-free remnants of lost capillaries. Furthermore, the authors also found evidence that BMECs are infected and that the death of BMECs in COVID-19 is secondary to SARS-CoV-2 infection. The SARS-CoV-2 genome encodes two viral proteases that are responsible for processing viral polyproteins into the individual components of the replication and transcription complexes. Wenzel et al. (2021) found that one of them, SARS-CoV-2 M^pro^, cleaves the host protein nuclear factor (NF)-κB essential modulator (NEMO), which is known to modulate cell survival and prevent apoptosis and necroptosis.

However, other findings suggest that SARS-CoV-2-related neurological complications may be a direct result of the neurovirulent properties of the virus (Shehata et al., 2021). Overall, it has been postulated that there are several different mechanisms involved in COVID-19–associated CNS dysfunction, including activation of inflammatory and thrombotic pathways and, in a few patients, a direct viral effect on the brain endothelium and the brain parenchyma (Bodro et al., 2021). However, further studies are needed to clarify the relative contribution of each of these mechanisms. A recent landmark study used three independent approaches to probe the capacity of SARS-CoV-2 to infect the brain (Song et al., 2021). In the first, transgenic mice overexpressing human ACE2 were found to support SARS-CoV-2 neuroinvasion. After intranasal administration, the virus was widely present in neural cells throughout the forebrain. In the second, using human brain organoids, clear evidence of infection with accompanying metabolic changes in infected and neighboring neurons was found. In this study, neuronal infection could be prevented by blocking ACE2. Finally, in autopsies from patients who died of COVID-19, SARS-CoV-2 was detected in cortical neurons. Remarkably, none of the regions of positive viral staining showed lymphocyte or leukocyte infiltration, indicating that SARS-CoV-2 did not invoke an immune response typical of other neurotropic viruses. These findings provide compelling evidence that the brain is a site for the high replicative potential for SARS-CoV-2 and that neurons can become a target of SARS-CoV-2 infection, with devastating consequences for localized ischemia in the brain and cell death, highlighting SARS-CoV-2 neurotropism.

The lipid-binding protein apolipoprotein E (ApoE) is the most abundant apolipoprotein in the brain (Flowers and Rebeck, 2020). It is produced predominantly by astrocytes and to some extent microglia. In addition, neurons upregulate ApoE expression in response to excitotoxic injury (Liao et al., 2017). As a major component of very low-density lipoproteins in the brain, ApoE facilitates the transfer of cholesterol and phospholipid between cells. ApoE has been linked with immune responses and neuroinflammation, metabolism, synaptic plasticity, transcriptional regulation, and vascular function by modulating cerebral blood flow, neuronal-vascular coupling, and BBB integrity (Liao et al., 2017). There are three major isoforms (ApoE2, ApoE3, and ApoE4) in humans (Liao et al., 2017). The most common isoform (77–78%) in the general population is E3, whereas E2 is evident in 7–8%, and E4 in 14–16% of individuals (Weisgraber and Mahley, 1996). ApoE4, a strong genetic risk factor for Alzheimer’s disease, is known to lead to BBB dysfunction (Montagne et al., 2020) and has been associated with increased risk for severe COVID-19 (Kuo et al., 2020). Recently, Wang et al. (2021a) tested the neurotropism of SARS-CoV-2 in human induced pluripotent stem cell (hiPSC) models and observed low-grade infection of neurons and astrocytes. They then generated isogenic ApoE3/3 and ApoE4/4 hiPSCs and found an increased rate of SARS-CoV-2 infection in ApoE4/4 neurons and astrocytes. ApoE4 astrocytes exhibited enlarged size and elevated nuclear fragmentation upon SARS-CoV-2 infection. These findings suggest that ApoE4 may play a causal role in COVID-19 severity.

Interestingly, ApoE4 has also been associated with seizures. For instance, spontaneous seizures were observed in aged ApoE4 targeted replacement (TR) mice but not in age-matched ApoE2 TR or ApoE3 TR mice (Hunter et al., 2012). In mice with overexpression of ApoE4 (but not ApoE2 or ApoE3), intranasal administration of kainate induced more severe seizures, increased microglial activation, and triggered more hippocampal damage than in wild-type mice (Zhang et al., 2012). In a case-control genetic association study in patients with mesial TLE and hippocampal sclerosis, ApoE4 carriers had an earlier onset of epilepsy than non-carriers (Leal et al., 2017). Thus, in summary, ApoE4 may play a role in seizures observed in viral infections, including COVID-19.

As described above, it is a matter of debate whether SARS-COV-2 can enter the brain, but several studies indicate that the SARS-CoV-2 S1 protein can be released from viral membranes, can cross the BBB, and is present in brain cells including neurons (Meinhardt et al., 2021; Rhea et al., 2021; Petrovszki et al., 2022). Thus, Datta et al. (2021) tested the hypothesis that SARS-CoV-2 S1 protein can directly induce neuronal injury. The latter authors found that the S1 protein accumulates in endolysosomes of human cortical neurons and induces aberrant endolysosome morphology and neuritic varicosities, which could contribute to the high incidence of neurological disorders associated with COVID-19.

Emerging data suggest that ∼10–40% of patients fail to fully recover after acute COVID-19 infection (Doyle, 2021; Nalbandian et al., 2021). Patients who report symptoms persisting for weeks or months after the acute illness have been termed “long haulers” or described as having “long-COVID.” Long-COVID comprises a variety of symptoms, of which the neurological component prevails, often characterized as post-infectious fatigue syndrome (Sandler et al., 2021). Furthermore, new-onset seizures in people with COVID-19 can potentially extend beyond the acute phase of the infection (Doyle, 2021). The most widely accepted theory on the genesis of these symptoms builds upon the development of microvascular dysfunction similar to that seen in numerous vascular diseases such as diabetes. This can occur through the peripheral activation of ACE2 receptors or through the exacerbating effects of pro-inflammatory cytokines that remain in circulation even after the infection diminishes (Nalbandian et al., 2021). However, at least in part, some of the mechanisms of CNS symptoms discussed above for the acute infection may also play a role in post-COVID symptoms.

### Virus-Specific Mechanisms: Human Herpesvirus-6

As discussed above, accumulating evidence suggests a pathogenic role of HHV-6B infection in the development of mesial TLE, and a relationship between viral load and markers that directly (CCL2) or indirectly (GFAP) reflect inflammatory or otherwise injurious processes. How might these observations associating mesial TLE with increased HHV-6 viral detection and increased markers of neuroinflammation and astrocyte activation be mechanistically associated with epilepsy? Inflammation and HHV-6 infection have each been demonstrated to induce dysregulation of glutamate homeostasis in astrocytes, which is hypothesized to play a central role in the pathogenesis of epilepsy (Leibovitch and Jacobson, 2015). *In vitro*, HHV-6 infection of primary astrocytes has been shown to downregulate levels of glutamate transporter expression, which supports the concomitant observation of decreased glutamate uptake in infected versus uninfected astrocytes (Fotheringham et al., 2008). Inflammatory cytokines, such as IL-1β, can also inhibit the astrocyte reuptake of glutamate (Vezzani and Baram, 2007). Because HHV-6–infected astrocytes have been demonstrated in mesial TLE, and because the virus can induce a metabolic dysregulation that is considered to contribute to epileptogenesis, this mechanism is biologically plausible (Leibovitch and Jacobson, 2015). Interestingly, ApoE4 has been suggested to increase viral load and seizure frequency in mesial TLE patients that are positive for HHV-6B DNA and protein in temporal lobe brain samples resected during epilepsy surgery (Huang et al., 2015).

### Virus-Specific Mechanisms: Flaviviruses Such as Tick-Borne Encephalitis Virus, West Nile Virus, Zika Virus, and Japanese Encephalitis Virus

Astrocytes exert many essential complex functions in the healthy CNS that are necessary to maintain synaptic and neural circuit homeostasis (Sofroniew and Vinters, 2010). Astrocytes respond to all forms of CNS insults, including viral encephalitis, through a process referred to as reactive astrogliosis, which has become a pathological hallmark of CNS structural lesions. Astrogliosis is a common step in the sequence of events that converts a normal brain into an epileptic brain after an acquired insult (Klein et al., 2018). Astrogliosis is involved in inflammatory processes as well as dysregulation of astroglial potassium and gap junction channels, which together alter glioneuronal communication and, by impairing uptake and redistribution of extracellular K^+^ accumulated during neuronal activity, can contribute to or cause seizures (Klein et al., 2018). Astrocytic compartmentalization of synapses also plays an essential role in neurotransmitter homeostasis by concentrating high levels of transporters for glutamate, GABA, and glycine that serve to clear these neurotransmitters from the synaptic space (Sofroniew and Vinters, 2010). During neuroinflammation, high levels of cytokines such as IL-6 lead to decreased glutamate uptake from the synaptic space by downregulating the excitatory amino acid transporter 2 (EAAT2; formerly glutamate transporter 1) on astrocytes, leading to glutamate accumulation and consequent neuronal hyperexcitability (Verhoog et al., 2020).

Within this context, it is important to note that astrocytes are thought to play a crucial role in flavivirus infections of the CNS by mediating the mechanisms that underlie neurological sequelae such as seizures and epilepsy (Potokar et al., 2019; Zheng et al., 2020; Ashraf et al., 2021). Indeed, given the anatomic position of astroglia and their homeostatic role in the CNS, one can predict that virus invasion may lead to important functional consequences for the entire CNS upon the interaction of astrocytes with viruses. Furthermore, in comparison to neurons, infected astrocytes produce orders of magnitude more virus, as demonstrated for ZIKV, TBEV, and WNV (Tavkar et al., 2021). This is highly relevant for the spread of infection through the CNS, especially because astrocytes are also more resilient to the lytic effects of flavivirus infection. Interestingly, different flavivirus strains appear to exert different effects on specific astrocyte responses (Potokar et al., 2019; Ashraf et al., 2021).

For example, TEBV, an important human pathogen that may result in dangerous neuroinfections (meningitis, meningoencephalitis, myelitis) and is endemic in Europe and Asia, replicates in astrocytes but does not typically affect astrocyte viability (Palus et al., 2014; Potokar et al., 2019). TBEV infection induces several morphologic and functional changes in infected rat and human astrocytes, including astrocyte activation as indicated by increased production of GFAP (Tavkar et al., 2021). Upon activation by TEBV infection, astrocytes release inflammatory cytokines and chemokines that may enhance neuronal excitability (Figure 5). TBEV infection of astrocytes may also alter the permeability of the BBB, as shown in mice (Ruzek et al., 2011). One of the key molecules that degrade the integrity of the BBB is matrix metalloproteinase 9 (MMP-9), which is overproduced in TBEV-infected astrocytes *in vitro* and increased in the serum and CSF of TBEV-infected patients (Potokar et al., 2019).

Upon WNV infection, astrocytes also release MMPs and pro-inflammatory cytokines, leading to disruption of the BBB and recruitment of leukocytes (Ashraf et al., 2021). Analysis of autopsied neural tissues from humans with WNV encephalomyelitis revealed WNV infection of both neurons and glia (van Marle et al., 2007). In human astrocytes and neurons, WNV replicates efficiently but distinctively with a higher and faster replication rate in astrocytes (Cheeran et al., 2005). Astrocytes have an active role in the spread of WNV in the CNS and in the maintenance of WNV neuroinvasive potential. Among the WNV-induced functional changes in astrocytes is the expression of endoplasmic reticulum stress-related genes linked to WNV neurovirulence (van Marle et al., 2007). WNV-infected astrocytes also upregulate the expression of several chemokines, but only after infection with the replication-competent virus and not with an inactivated virus (Potokar et al., 2019). In an experimental murine model of WNV-induced seizures, intranasal inoculation with WNV caused limbic seizures in B6 mice, but not in IFN-γ-deficient (IFN-γ^–/–^) mice (Getts et al., 2007). Both strains showed similar levels of virus in the brain, as well as similar concentrations of TNF-α and IL-6, both of which alter neuronal excitability. However, TNF-α deficient mice infected intranasally with WNV still developed severe limbic seizures, similar to B6 wild-type mice (Getts et al., 2007). While the absence of seizures in the infected IFN-γ^–/–^ mice was shown to be associated with the influence of this cytokine on excitatory circuit development, rather than a direct effect on synaptic function, *per se*, the observation highlights the complicated relationship between inflammation and CNS function. Finally, in patients with WNV encephalitis, increased infiltration of monocytes into the brain was found (Ashhurst et al., 2013), which, as discussed elsewhere in this review, appears to be a common outcome of CNS infection.

In addition to the profound impact on fetuses and neonates (fetal growth restriction, abnormalities of the CNS, including microcephaly) caused by intrauterine infections with ZIKV during pregnancy, this virus can also cause neurologic symptoms in adults (Guillain-Barré syndrome, myelitis, encephalitis, and neuralgia) (Potokar et al., 2019). Following infection of immunocompetent pregnant mice with ZIKV, we found the virus particularly in glial cells, such as astrocytes, oligodendrocytes, and microglia, most profoundly in the brainstem and cerebellum of the maternal brain (Stanelle-Bertram et al., 2018). Interestingly, the male offspring from ZIKV infected mothers were more likely to suffer from impairment of learning and memory compared to females, likely as a result of more severe neuropathological alterations in the hippocampus compared to their female littermates (Stanelle-Bertram et al., 2018). Furthermore, in a study in which perinatal infection was simulated by using neonatal mice, seizures were observed following subcutaneous inoculation of 1-day-old immunocompetent B6 mice with ZIKV PRVABC59 (Manangeeswaran et al., 2016). The seizures were associated with ZIKV infection in the brain, neurodegeneration in the hippocampus and cerebellum, and infiltration of brain tissue with CD4^+^ and CD8^+^ T cells. In a study with ZIKV infection in 3-days-old Swiss mice, the animals developed frequent seizures during the acute phase, which were reduced by inhibiting TNF-α (Nem de Oliveira Souzaem et al., 2018). During adulthood, ZIKV replication persisted in neonatally infected mice, and the animals showed increased susceptibility to chemically induced seizures and neurodegeneration, predominantly in the hippocampus, thalamus, striatum, and cortex. Both cell death and impaired proliferation of neural precursors were shown to underlie ZIKV-induced neuropathology (Nem de Oliveira Souzaem et al., 2018). In a subsequent study from the same group, the effects of ZIKV infection on neuronal networks (determined from electrophysiological activity) and how different mechanisms can trigger epilepsy in ZIKV Swiss mice were examined (Pinheiro et al., 2020).

Astrocytes, together with microglia, are proposed to be major ZIKV targets in fetal brain development (Potokar et al., 2019). Primary fetal human astrocytes particularly stand out for their susceptibility to ZIKV infection in comparison with neurons and neural progenitor cells. As is the case for TBEV, astrocytes are also proposed to serve as a reservoir for ZIKV, and they apparently induce neuroinflammation through pro-inflammatory cytokines mediating synaptic and cognitive changes (Potokar et al., 2019).

As with other flaviviruses, astrocytes are also an important player in altered BBB permeability in response to JEV. Upon infection with JEV, astrocytes release vascular endothelial growth factor (VEGF), IL-6, and MMPs (Potokar et al., 2019). In addition to affecting the BBB, astrocytes are also involved in neuroinflammatory responses in the JEV-infected CNS that may underlie ictogenesis.

### Virus-Specific Mechanisms: Picornaviruses

The family of small, positive-sense, single-stranded, non-enveloped RNA viruses known as the *Picornaviridae* includes numerous human pathogens with known and potential neurovirulence (Rotbart, 1995; Buenz and Howe, 2006), including members of the *Enterovirus* genus such as poliovirus, the echoviruses, the Coxsackie viruses, and the rhinoviruses. The global ubiquity of these viruses, the high transmissibility, and the widespread exposure experienced by children make picornaviruses an important component of emerging or re-emerging infections associated with neurological disease (Fischer et al., 2022). For example, enterovirus 71 (EV71), the causative pathogen in hand, foot, and mouth disease, was originally isolated in California in 1969 from a 9-month old girl with encephalitis (Schmidt et al., 1974). Further outbreaks of this and related serotypes occurred across the US, South America, Europe, and Asia, with hundreds of thousands of infections in Asia-Pacific countries since the 1990s and thousands of deaths due to encephalitis or encephalomyelitis (Puenpa et al., 2019). Notably, while seizures are reported in some of these patients (Bissel et al., 2015), a predominant outcome for children with neurologic manifestations is death, suggesting that neurovirulent picornaviruses induce severe neuropathology. As we and others have discussed, several picornavirus proteins, including the structural proteins VP1, VP2, and VP3 and the non-structural proteins 2A and 3C directly engage pro-apoptotic mechanisms in infected cells (Buenz and Howe, 2006) and co-opt antiviral mechanisms (Wang et al., 2018). However, seizures are clearly a component of picornaviral infections in less severe cases, including a broad propensity to febrile seizures, acute seizures, and late spontaneous seizures (Table 1).

Picornavirus neurotropism is obviously well established for human poliovirus (Whitton et al., 2005). The human poliovirus receptor CD155 is enriched in anterior horn motor neurons (Gromeier et al., 2000) and mediates cellular entry, as proven by neuronal infection and development of paralytic poliomyelitis in mice transgenically expressing CD155 (Ren et al., 1990). Other picornaviruses exploit different cellular receptors. For example, both EV71 and coxsackievirus A16 (CVA16) utilize scavenger receptor class B, member 2 (SCARB2; aka CD36L2) to enter cells. This protein, widely and highly expressed in the brain, gut, and immune system, localizes to neurons, and transgenic expression of human SCARB2 in mice renders the host susceptible to CNS infection with EV71 (Fujii et al., 2013). While the pathophysiological relevance is not clear, it is notable that mutations in SCARB2 are associated with epilepsy (Rubboli et al., 2011).

Given the broad expression of picornavirus receptors, the development of focal neurological sequelae must depend upon cell-intrinsic responses to infection or cell-specific sensitivity to innate and/or adaptive immune responses elicited by CNS infection. Concerning the former, one potential mechanism of neuronal specificity arises from the rapid and robust shutdown of host cell translation that is a hallmark of picornavirus infection (Etchison et al., 1982) and is mediated by viral protease cleavage of cap-dependent translation factor eIF-4G (Whitton et al., 2005). While cap-dependent translation is important to all cells, neurons may exhibit a unique sensitivity to translation inhibition. For example, evidence from ischemia-reperfusion models indicates that vulnerable neuronal populations in the hippocampus selectively undergo apoptosis in response to downregulated protein synthesis (Ayuso et al., 2013). Likewise, specific neuronal populations may be uniquely sensitive to the activation of stress pathways activated by translation inhibition, such as NFκB activation due to loss of IκBα translation and suppression of AKT signaling as part of the integrated stress response (Kapur et al., 2017). In parallel, suppression of glutamate transporter expression and local neuroinflammatory responses that result in the release of factors such as TNFα may combine to drive both hyperexcitability and accelerated neuronal cell death (Guerrini et al., 1995; Kaltschmidt et al., 1995; McCoy and Tansey, 2008). Finally, concerning neuron-specific sensitivity to infection-induced neuroinflammatory responses, robust evidence obtained using the mouse picornavirus TMEV, outlined below, indicates that innate immune cell-mediated acute antiviral responses lead to both neuronal cell death and dysregulation of electrophysiological homeostasis.

## Animal Models to Study Mechanisms of Seizures and Epilepsy After Viral Infections

As discussed above, animal models are useful to study the mechanisms involved in infection-induced ictogenesis (i.e., the generation of seizures) and epileptogenesis (i.e., the generation of epilepsy). Various animal species, including rabbits, rats, and mice have been infected with neurotropic viruses and develop early (encephalitis-associated) seizures, but most die following the acute viral encephalitis phase so the processes leading to epilepsy cannot be investigated (Vezzani et al., 2016). One important exception is the infection of mice with TMEV, which will be discussed in the next section.

A significant advantage of animal studies is that they allow for the examination of genetic background as a variable for the host response (cf., Figure 1) to virus infection (Kollmus et al., 2018). Furthermore, animal models permit the invasive mechanistic dissection of *in vivo* processes underlying virus-induced CNS alterations that cannot be examined in patients. One recent example is the infection of mice with a low dose of a mouse-adapted non-neurotropic IAV (H1N1), which caused ample peripheral immune response followed by a temporary BBB disturbance (Düsedau et al., 2021). Although histological examination did not reveal obvious pathological processes in the brains of IAV-infected mice, a closer evaluation revealed a subtle dysbalance in glutamatergic synapse transmission in the cortex and hippocampus upon H1N1 infection. Previous experiments using IAV/H1N1 infection models have shown subtle alterations in hippocampal neuronal morphology and impairment of cognitive abilities in the absence of virus in the brain (Jurgens et al., 2012; Hosseini et al., 2018), thus demonstrating the importance of host response mediated effects as illustrated in Figure 1. In line with these findings, neuropsychiatric complications including seizures were not only reported after infection with neurotropic IAV variants but also after non-neurotropic H1N1 virus infection, especially in children (Ekstrand et al., 2010; Surana et al., 2011).

A variety of animal models to study viral infections are available, including models of herpesvirus encephalitis (Reynaud and Horvat, 2013; Sehl et al., 2020), COVID-19 (Munoz-Fontela et al., 2020), ZIKV infections (Morrison and Diamond, 2017), HIV, IAV and Dengue virus infections (Krishnakumar et al., 2019), and multiple other encephalitic viruses, including JEV, WNV, and TBEV (Holbrook and Gowen, 2008). The most commonly used model species include mice, hamsters, rats, rabbits, guinea pigs, ferrets, cats, dogs, minks, pigs, chickens, ducks, fruit bats, and non-human primates. Mice have an important advantage in that the development of humanized mouse models offers a preclinical *in vivo* platform for further characterization of human viral pathogens and human antiviral immune responses (Lai and Chen, 2018). A recent example is the use of transgenic mice that express human ACE2 as a model for SARS-CoV-2 infection (Munoz-Fontela et al., 2020).

However, with few exceptions, animal models of virus infections have not been used in the past to study the mechanisms of seizures. One explanation in this regard is that seizures, either early or late, are easily overseen if not monitored by laborious techniques, including continuous (24/7) EEG and video monitoring (Löscher, 2016). The most important example of an animal model of viral encephalitis that has been extensively used to study the molecular mechanisms of seizures and epilepsy is described in the following section.

## The Theiler’s Murine Encephalomyelitis Virus Mouse Model to Study Mechanisms of Seizures and Epilepsy in the Central Nervous System

**FIGURE 6:**
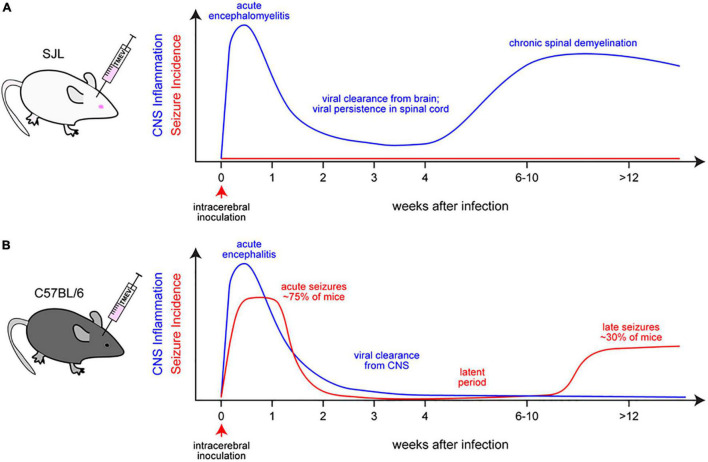
Theiler’s murine encephalomyelitis virus (TMEV) infection as a model for multiple sclerosis **(A)** or epilepsy **(B)**. **(A)** Intracerebral inoculation of SJL mice with low neurovirulent Theiler’s original (TO) subgroup strains of TMEV (such as DA or BeAn) results in a biphasic disease course consisting of an acute encephalomyelitis followed by demyelination of the spinal cord. During the acute phase, neurons within the hippocampus, cerebral cortex, and spinal cord are infected. However, later, as the virus is cleared from the brain, persistent infection in spinal glia results in chronic inflammation, demyelination, axonal degeneration, and astrogliosis, resembling the pathological alterations observed in MS. Notably, seizures are not observed in TMEV-infected SJL mice at any point during the disease course. **(B)** Intracerebral inoculation of C57BL/6 mice with TMEV results in acute viral encephalitis that is marked by rapid monocytic and neutrophilic infiltration followed by entry of antiviral T cells, resulting in viral clearance from the host. The virus does not persist in these animals. In contrast to SJL mice, damage to hippocampal neurons is a prominent feature in B6 mice and is associated with impaired learning and memory and induction of anxiety behaviors. Early symptomatic seizures occur in ∼75% of mice during the acute phase and are associated with monocyte-derived cytokines such as IL-6 and TNF-α. The acute symptomatic seizures are observed over ∼2–10 days following infection, followed by a seizure-free latent phase of several weeks, after which about 30% of the infected mice develop spontaneous seizures.

We and others have used the TMEV mouse model to study the mechanisms underlying seizure generation after virus infection of the CNS. TMEV, a non-enveloped, positive-sense, ssRNA virus of the *Picornaviridae* family and *Cardiovirus* genus, is a naturally occurring enteric pathogen of the mouse (Libbey and Fujinami, 2011). It was discovered by Nobel laureate Max Theiler in the 1930s (Theiler, 1934). TMEV causes enteric infection in mice *via* the fecal-oral route of transmission. While these infections are usually asymptomatic or mild, the virus can spread to the CNS and cause encephalitis and/or encephalomyelitis. Using different substrains of the virus, Theiler observed encephalomyelitis that was associated either with flaccid paralysis or seizures (Theiler, 1934; Theiler, 1937; Theiler and Gard, 1940a). He also described that the mouse virus is very rarely present in the CNS of normal mice but that intracerebral inoculation of mice with filtrates prepared from the intestinal contents of normal mice induced encephalomyelitis and the associated neurological phenotype (Theiler and Gard, 1940b). Due to the development of chronic inflammatory demyelinating disease in susceptible mouse strains such as SJL, intracerebral infection of such mice with the Daniels (DA) or BeAn 8386 (BeAn) strains of TMEV has been used as an animal model for MS for approximately the past 50 years (Libbey and Fujinami, 2021; Figure 6A). The T cell-mediated autoimmune demyelinating disease in SJL mice is characterized by weakness of the hind limbs, which advances to severe spastic paralysis, and inflammatory demyelination in the spinal cord. The B6 mouse has been used as the classic “resistant” mouse strain, which does not develop the demyelinating disease (Libbey and Fujinami, 2011). One important difference between SJL and B6 mice is that SJL mice are unable to adequately control the virus and therefore develop persistent TMEV infection that induces a smoldering neuroinflammatory environment that facilitates demyelination, particularly in the spinal cord. In contrast, the virus is rapidly cleared by B6 mice, which was thought to explain the resistance to neurological consequences of TMEV infection (Gerhauser et al., 2019; Figure 6B). The mechanisms underlying this striking difference between the strains seem to be partially due to the strong antiviral cytotoxic CD8^+^ T lymphocyte response that occurs in B6 mice, which is suppressed by the elevated induction of regulatory CD4^+^ T cells (Tregs) in SJL/J mice (DePaula-Silva et al., 2017).

In addition to the initial description of flaccid paralysis induced by TMEV in some mice, Theiler also noted that some strains of the virus induced disease in which the “outstanding clinical sign was an extreme hyperexcitability” (Theiler and Gard, 1940a). He further noted that these animals “would jump about excitedly at the slightest stimulus,” performed “rubbing movements of the face,” and exhibited “tonic convulsions with the hind limbs extended and the fore limbs flexed.” Theiler indicated that “death might occur during one of these seizures” and that infected mice showed “marked encephalitis” in the absence of frank paralysis (Theiler and Gard, 1940a). However, this aspect of the TMEV model was largely overlooked for 50 years–regrettably, we noted seizures in one of our early studies as an exclusion criterion for behavioral assessment in B6 mice infected with the virus (Buenz et al., 2006)! It wasn’t until the foundational report from Libbey et al. in 2008 that the field came to recognize the value of the TMEV model for understanding seizures induced by viral encephalitis. These investigators reported that approximately 50% of B6 mice (male and female) infected intracerebrally with the DA strain of TMEV developed acute behavioral seizures that occurred between 3 and 10 days after virus inoculation. As with Theiler’s original observation, slight cage shaking, handling, or loud noises facilitated the occurrence of early seizures, which were rated by the Racine score (Racine, 1972). Most early seizures were generalized convulsive (Racine stage 5) seizures (Libbey et al., 2008). A similar percentage of early seizures was also observed when infecting B6 mice with the BeAn strain of TMEV (Libbey et al., 2011). The incidence of such seizures increased to 75% if continuous video-EEG was used to monitor the mice (Stewart et al., 2010a). Furthermore, Stewart et al. (2010a) reported that a significant proportion of mice experiencing acute seizures later developed spontaneous epileptic seizures with hippocampal sclerosis (Figure 6B), which is a hallmark of TLE (Blümcke et al., 2002; Thom, 2014). In B6 mice, TMEV has a specific tropism for the CA1 and CA2 pyramidal cell layers of the hippocampus; periventricular thalamic nuclei; septal nuclei; and piriform, parietal, and entorhinal cortices during acute TMEV infection (Libbey et al., 2008; Buenz et al., 2009; Stewart et al., 2010a,b). Unlike B6 mice, TMEV-infected SJL mice show subclinical, transient polioencephalitis along with mild neuronal degeneration, which is not accompanied by seizure development in the acute disease (Libbey et al., 2008; Figure 6A). SJL mice are typically protected from hippocampal damage by TMEV, which seems to be mediated by IL-10 receptor signaling (Uhde et al., 2018).

Theiler’s murine encephalomyelitis virus infection in B6 mice was the first animal model to associate viral encephalitis with epilepsy, thus allowing the field to study the mechanisms underlying the development of early and late seizures.

### Potential Role of Invading Monocytes and Resident Microglia in the Mechanisms Underlying Seizure Generation in the Theiler’s Murine Encephalomyelitis Virus Model

Based on a large series of subsequent studies of the groups of Robert S. Fujinami, H. Steve White, and Karen S. Wilcox at the University of Utah, which were reviewed by Libbey and Fujinami (2011) and DePaula-Silva et al. (2017, 2021), it was suggested that infiltrating monocytes (CD45^hi^ CD11b^+^) present in the brain of B6 mice at day 3 post-infection are an important source of IL-6, which critically contributes to the development of acute seizures in the TMEV-induced seizure model. Furthermore, the production of high levels of TNF-α by microglia during the acute phase of the infection was found to play a role (Cusick et al., 2013). When mice deficient in TNF receptors, TNF-α or IL-6 were infected with TMEV, the incidence of acute seizures was significantly decreased, whereas IL-1R1 deficient mice did not differ from wild-type controls (Kirkman et al., 2010; Patel et al., 2017). From these data and the known effects of IL-6 and TNF-α on neuronal activity, it was suggested that IL-6 and TNF-α secreted in the brain by infiltrated monocytes and resident microglia during TMEV infection in B6 mice may contribute to enhanced glutamatergic excitation and decreased GABAergic inhibition and lead to a more seizure prone state (DePaula-Silva et al., 2021). In support of this hypothesis, TMEV infection of B6 mice depleted of monocytes resulted in a significant decrease in the number of mice experiencing seizures, substantiating a role for infiltrating monocytes in the development of acute seizures in the TMEV-induced seizure model (Cusick et al., 2013). However, at least in part, the experimental methods used to reduce monocyte invasion and distinguish monocytes/macrophages from microglia were not specific, so the exact role and interplay of these and other immune cells in the TMEV model remained elusive.

### The Role of Structural vs. Inflammatory Brain Alterations in the Mechanisms Underlying Seizure Generation in the Theiler’s Murine Encephalomyelitis Virus Model

The interesting data reported by the University of Utah groups prompted W. Löscher’s group to establish the TMEV model in B6 mice in Hannover, Germany. Unexpectedly, it took several years to reproduce the seizure phenotype in our laboratory (Bröer et al., 2016). Indeed, the BeAn strain of TMEV was used in thousands of SJL/J and B6 mice by Wolfgang Baumgärtner’s group at the Department of Pathology at the University of Veterinary Medicine in Hannover over a period of ∼15 years in numerous studies on mechanisms involved in virus-induced demyelination but seizures were never observed in B6 mice. Thus, we hypothesized that either the substrain of B6 mice or the BeAn substrain used in these experiments may have been responsible for the lack of seizures. This hypothesis was addressed by comparing two B6 and two BeAn substrains, including the mouse and virus substrains used in the original studies of Fujinami and White (Bröer et al., 2016). In addition, we compared the potency of the BeAn and DA TMEV strains to induce seizures and epilepsy in mice. The idea behind this approach was to study what is and what is not necessary for the development of acute and late seizures after brain infection in mice. Receiver operating characteristic (ROC) curve analysis was used to determine which virus-induced brain alterations are associated with seizure development. In B6 mice infected with different TMEV virus (sub)strains, the severity of hippocampal neurodegeneration, amount of MAC3-positive microglia/macrophages, and expression of ISG15 were almost perfect at discriminating seizing from non-seizing B6 mice, whereas T-lymphocyte brain infiltration was not found to be a crucial factor (Bröer et al., 2016).

The potential role of blood-borne monocyte brain invasion for the seizure phenotype induced by TMEV infection of B6 mice suggested by the University of Utah groups (Libbey and Fujinami, 2011; DePaula-Silva et al., 2017, 2018, 2021) prompted us to perform a series of studies using either genetic or pharmacological strategies. The outcome of these studies is summarized in Table 3. First, to better differentiate brain-resident myeloid cells, including microglia, from invading monocytes in the TMEV encephalitis model of TLE, we compared virus-induced effects in B6 WT vs. B6-based *Cx3cr1-cre*^ER±^*tdTomato*^St/Wt^ mice, in which long-lived CX3CR1^+^ cells such as microglia can be distinguished from infiltrating monocytes by the expression of the red fluorescent protein tdTomato (Käufer et al., 2018a). When using flow cytometry to differentiate blood-borne monocytes (CD45^high^CD11b^+^) from resident microglia (CD45^low^CD11^+^) in the brain, the *Cx3cr1-cre*^ER±^*tdTomato*^St/Wt^ reporter mice provided qualitative proof that activated myeloid cells present in the CNS after TMEV infection consist of microglia and infiltrating monocytes (Table 3), although concerning CD45 and CD11b expression, some microglia become indistinguishable from monocytes during CNS infection (Käufer et al., 2018a).

**TABLE 3:** A summary of the experiments of the Löscher lab on TMEV-induced seizures and epilepsy.

Approach (C57BL/6 mice)	Monocyte invasion	Microglia proliferation	Hippocampal damage	Early seizures	Late seizures (epilepsy)	References
Control (wild-type)	+	Ø	+	+	+	Bröer et al., 2016; Bröer et al., 2017; Anjum et al., 2018; Käufer et al., 2018a; Waltl et al., 2018a,b
**Pharmacological manipulation**						
Clodronate liposomes	⇓	Ø	+	⇓	+	Waltl et al., 2018a,c
PLX5622	+	⇓	⇑	⇑	TBD	Waltl et al., 2018b,c
**Genetic manipulation**						
*Cx3cr1* reporter mice (*Cx3cr1*^CreER±^*td-Tomato*^St/wt^)	+	Ø	+	+	TBD	Käufer et al., 2018a
*Ccr2*-KO mice	⇓	Ø	⇓	+	+	Käufer et al., 2018a,b
*Cx3cr1*-KO mice	(⇓)	Ø	⇓	+	+	Käufer et al., 2018a,b

*Data are from TMEV-infected C57BL/6 mice, using the DA strain of TMEV for intracerebral inoculation.*

*In addition to data from flow cytometry shown in the table for monocyte invasion and microglia proliferation, neuroinflammation was also assessed by immunohistochemistry (Iba1, Mac-3, CD3), T cell and neutrophil infiltration, and qPCR (cytokines). A significant increase in infected wild-type controls or Cx3cr1 reporter mice compared to sham-infected controls is indicated by “+” and lack of such alteration by “Ø”. A significant decrease or increase by pharmacological or genetic manipulation compared to infected wild-type controls is indicated by arrows. TBD, to be determined.*

Next, we used two pharmacological approaches to determine the impact of invading monocytes vs. resident microglia for early seizures and hippocampal damage induced by TMEV in B6 mice. When using systemic administration of liposome-encapsulated clodronate liposomes as a selective and widely used approach for monocyte depletion, almost complete depletion of monocytic cells was achieved in the spleen and blood of Theiler’s virus-infected B6 mice, which was associated with a 70% decrease in the number of brain-infiltrating monocytes as assessed by flow cytometry (Waltl et al., 2018a). As shown in Table 3, significantly fewer clodronate liposome-treated mice exhibited seizures than liposome controls. The severity of seizures was not affected by monocyte depletion, but the seizure burden (the number of seizures per mouse observed over 7 days after infection) was markedly reduced (Waltl et al., 2018a). However, the development of hippocampal damage was not prevented or reduced by monocyte depletion (Table 3).

Surprisingly, clodronate liposome treatment did not reduce the increased Iba1 and Mac3 labeling in the hippocampus of infected mice, indicating that activated microglia may contribute to hippocampal damage (Waltl et al., 2018a). Thus, our next pharmacological approach used prolonged administration of PLX5622, a specific inhibitor of colony-stimulating factor 1 receptor that depletes microglia (Waltl et al., 2018b). As shown in Table 3, microglia depletion accelerated the occurrence of seizures, exacerbated hippocampal damage, and led to neurodegeneration in the spinal cord, which is normally not observed in B6 mice. These data suggested that microglia are required early after infection to limit virus distribution and persistence, most likely by modulating T cell activation (Waltl et al., 2018b). An antiviral role of microglia has also been demonstrated for ZIKV, HSV, JEV, WNV, and several other virus infections (Terry et al., 2012; Chen et al., 2019). Interestingly, TNF-α expression in the brain of TMEV-infected mice was not affected by microglia depletion, suggesting that CNS and/or infiltrating cells other than microglia are also secreting this cytokine (Waltl et al., 2018b). More recently, our data have been partially confirmed by the University of Utah groups (DePaula-Silva et al., 2018; Sanchez et al., 2019).

In an additional series of experiments, we used genetic approaches (*Ccr2*-KO and *Cx3cr1*-KO mice) to study the role of invading monocytes vs. activated microglia for early seizures and hippocampal damage (Käufer et al., 2018a). CCR2 and CX3CR1 are two chemokine receptors that regulate the responses of myeloid cells, such as monocytes and microglia, during inflammation (Prinz and Priller, 2010). Based on their differential expression of the chemokine receptors CCR2 and CX3CR1 in mice, so-called “inflammatory” (or “classic”) monocytes (CCR2^+^CX3CR1^low^), which are highly mobile and rapidly recruited to inflamed tissues, can be distinguished from patrolling (non-classic) monocytes (CCR2^–^CX3CR1^high^), which are larger in size and patrol along vascular endothelium such as the BBB (Prinz et al., 2011; Prinz and Priller, 2017). Brain-resident microglia produce the myelo-attractant cytokine CCL2 (also known as MCP1), a CCR2 ligand that promotes the transmigration of CCR2^+^ monocytes (and T cells) across the BBB *via* CCL2/CCR2 crosstalk (Prinz and Priller, 2010; Howe et al., 2017). Mice devoid of the *Ccr2* gene exhibit markedly reduced recruitment of monocytes and reduced pathology in several brain disease models, including autoimmune encephalitis, MS, stroke, and status epilepticus (Prinz and Priller, 2010; Chu et al., 2014; Varvel et al., 2016). Interestingly, in SJL mice, in which infection with TMEV induces severe spinal cord demyelination (Figure 6A), the use of *Ccr2*-KO mice reduced monocyte infiltration, demyelination, and long-term disease severity (Bennett et al., 2007).

As shown in Table 3, in B6 mice, the lack of CCR2 or CX3CR1 receptors was associated with a significant reduction of monocyte invasion and almost complete prevention of hippocampal damage but did not prevent seizure development after viral CNS infection (Käufer et al., 2018a). These data are compatible with the hypothesis that CNS inflammatory mechanism(s) other than the infiltrating myeloid cells trigger the development of seizures during viral encephalitis. It is also important to note that the consequences of pharmacological vs. genetic manipulation of monocyte invasion and microglia activation strikingly differed (Table 3). Furthermore, the interplay between microglia and invading monocytes in this model is more complex than previously proposed by other groups (Libbey and Fujinami, 2011; DePaula-Silva et al., 2017, 2018, 2021).

All studies described thus far examined the role of various manipulations on the occurrence of *early* seizures and hippocampal damage in the TMEV model in B6 mice. As described above, a fraction of the mice also develops spontaneous recurrent seizures, i.e., epilepsy after a latent period of several weeks (Figure 6B). In the experiments of the Löscher group, the incidence of epilepsy was determined by continuous (24/7) video-EEG monitoring, resulting in an epilepsy incidence of 33%, while the incidence of early seizures was 77% (Anjum et al., 2018). When determining the development of epilepsy in mice following treatment with clodronate liposomes or in *Ccr2*-KO and *Cx3cr1*-KO mice, no significant difference from controls was observed (Käufer et al., 2018b; Waltl et al., 2018c). This would suggest that–as outlined above–the mechanisms underlying early and late seizures are different. In this respect, it is interesting to note that although there are significant increases in amplitude and frequency of spontaneous and miniature excitatory currents (mediated by glutamate) in hippocampal CA3 neurons recorded in brain slices prepared during the acute infection period and during chronic epilepsy 2 months after infection, the patterns of changes observed are markedly different during these two periods, suggesting that there are underlying changes in the network over time (Smeal et al., 2012). In addition to the changes in excitatory currents of CA3 neurons both during the acute infection and 2 months later shown by Smeal et al. (2012), additional experiments disclosed a decrease in CA3 inhibitory network activity (mediated by GABA) during the acute infection, but not at the 2-month time point, again suggesting different mechanisms of seizure generation during the acute infection and during chronic epilepsy (Smeal et al., 2015).

In addition to epilepsy as a long-term outcome of TMEV infection in B6 mice, these animals also exert behavioral and cognitive alterations, such as increased anxiety, decreased pentylenetetrazole seizure threshold, and impaired learning and memory (Umpierre et al., 2014; Barker-Haliski et al., 2015). Treatment of mice with minocycline, but not valproic acid, during the acute phase of the TMEV infection improved long-term behavioral outcomes in the TMEV model (Barker-Haliski et al., 2016), but epilepsy was not monitored in this study. Minocycline was used in this study to directly suppress microglial activation and overexpression of inflammatory cytokines.

In summary, our data on the TMEV model suggest that hippocampal damage is not critically involved in ictogenesis and epileptogenesis, because genetic manipulations that completely prevented the damage did not modify the incidence of early or late seizures (Käufer et al., 2018a,b). The mismatch between findings of genetic versus pharmacological manipulations in TMEV-infected B6 mice illustrated in Table 3 deserves further study.

### Focusing on Acute Inflammatory Monocyte Infiltration as the Driver of Seizures and Neuropathology in the Theiler’s Murine Encephalomyelitis Virus Model

In parallel with the elegant work of the Utah and Hannover groups, the Howe group also came to recognize the importance of infiltrating inflammatory monocytes. Our initial studies probed the neuropathological and behavioral sequelae of TMEV encephalitis in B6 mice, revealing that pyramidal neurons in the CA1 region of the hippocampus were selectively lost by 4 days after intracerebral inoculation with Daniel’s strain of TMEV and that mice tested in the Morris water maze starting at 11 days after infection exhibited a profound disruption in the ability to form spatial memories (Buenz et al., 2006). We showed that memory impairment was associated with damage to the CA1 region in two ways. First, the increasing hippocampal injury was associated with a graded loss in the ability to learn the maze; second, mice with any amount of hippocampal damage converted from a spatial memory strategy to a cue-based escape strategy. At the time, we focused on the role of neurotropic viruses in the direct killing of hippocampal neurons (Buenz and Howe, 2006) and we postulated that low-level neurovirulence amongst the human picornaviruses results in widespread erosion of cognitive reserves in humans, potentially explaining the development of memory and cognitive impairments with age in the absence of clear etiology.

Unexpectedly, however, our follow-up studies indicated that apoptosis of hippocampal neurons during acute TMEV encephalitis occurred independently of direct cellular infection (Buenz et al., 2009). Indeed, while many infected mice exhibited nearly complete loss of all CA1 neurons in the dorsal hippocampus, only a small fraction of these neurons expressed TMEV antigen before death. This is consistent with our contention that only 20–2000 cells in the brain are infected with TMEV in the immediate aftermath of inoculation (Howe et al., 2017). Moreover, we showed that CA1 neurons exhibited evidence of oxidative injury and apoptotic processes as early as 2 days after inoculation, with peak neuronal death occurring within 4 days, a timeline that is inconsistent with any effect of antiviral adaptive immune-mediated mechanisms (Dethlefs et al., 1997; Mendez-Fernandez et al., 2003). Given the virus-independent nature of the CA1 pyramidal neuron death, we next sought to protect these neurons by interfering with the apoptotic cascade. We observed that calpain was specifically activated in CA1 neurons as early as 2 days after TMEV inoculation, prompting us to treat infected animals with ritonavir, a drug designed as an HIV protease inhibitor that also suppresses calpain (Howe et al., 2016). We found that ritonavir therapy almost completely prevented the loss of CA1 pyramidal neurons, without impacting viral fitness or eventual viral clearance. Moreover, we found that calpain inhibition preserved cognitive performance in the Morris water maze, protected novel object recognition learning, and completely prevented the development of acute, high Racine score seizures. Critically, this therapeutic effect was achieved even when therapy was started at 36 h after inoculation, a timepoint at which mice already exhibit low Racine score events and encephalitis is well established (Howe et al., 2016).

In parallel with the ritonavir work, we published two studies showing that inflammatory monocytes are the primary driver of hippocampal injury and cognitive impairment in the TMEV model. In the first, we showed that inflammatory monocytes infiltrate the TMEV inoculated brain within hours (Howe et al., 2012a)–indeed, in our most recent work we have observed these cells in the hippocampus within 3 h of inoculation. We defined these cells as CD45^hi^CD11b^+^ cells that are positive for Ly6C and Ly6B but negative for Ly6G. We also established that the LysM:GFP mouse generated by David Sacks (Faust et al., 2000) (*not* based on the LysM-Cre line) permitted the clear delineation of infiltrating inflammatory monocytes (GFP^mid^), infiltrating neutrophils (GFP^hi^), and microglia (GFP^neg^). Furthermore, we showed that immunodepletion of monocytes but not neutrophils preserved cognitive performance in the Morris water maze and protected the hippocampus from injury.

In the second study, we showed that despite equivalent viral load and acute encephalitis, SJL mice do not exhibit any injury to the hippocampus and this effect was genetically dominant, as the F1 offspring of SJL × B6 mice also showed hippocampal preservation (Howe et al., 2012b). Relevant to the discussion above regarding the direct viral killing of neurons, we also observed large numbers of intact CA1 pyramidal neurons loaded with TMEV antigen at 3 days after inoculation; these neurons eventually clear the virus non-lytically and remain intact. Strikingly, we found that SJL mice exhibited a markedly truncated inflammatory monocyte response at 24 h after inoculation, while neutrophil infiltration levels were the same or greater than B6 mice at the same timepoint. The B6 response profile was recapitulated in B10.S mice (Patick et al., 1990) (a C57BL/10 congenic line that expresses H-2^s^ and is therefore histocompatible with SJL mice) and we used bone marrow reconstitution to create chimeric animals with a B10.S nervous system and SJL immune system or an SJL nervous system with a B10.S immune system. We found that reconstitution of SJL mice with a B10.S immune system resulted in robust inflammatory monocyte infiltration and consequent hippocampal injury that was indistinguishable from B10.S mice reconstituted with B10.S bone marrow. Finally, we showed that adoptive transfer of Ly6C^+^Ly6G^–^ B6 peritoneal exudate monocytes (induced by mineral oil) into B6 × SJL F1 hosts at 18 h after TMEV inoculation led to profound hippocampal injury and abrogation of scent-based novel object recognition learning.

Given the acute timing of the inflammatory monocyte response and the rapid initiation of hippocampal injury and behavioral seizures in B6 mice, we sought to identify the molecular and cellular sources driving leukocyte recruitment to the CNS. We found that by 3 h after intracranial inoculation of TMEV the hippocampus exhibited a profound upregulation of inflammatory chemokine transcripts that was quickly followed by upregulation of inflammatory cytokine RNA (Howe et al., 2017). Moreover, we observed that serum CCL2 levels peak at 3 h after infection and this was temporally associated with high levels of CCL2 in the brain and hippocampus. Genetic deletion of CCR2 essentially abrogated inflammatory monocyte infiltration, while systemic immunodepletion of CCL2 but not CCL7 also truncated the monocytic response during acute TMEV encephalitis. Unexpectedly, we found that CCL2 was predominantly expressed by hippocampal neurons at 6 h after TMEV inoculation and we showed that neuron-specific deletion of CCL2 (Syn-Cre × CCL2-RFP^fl/fl^) resulted in complete suppression of serum and hippocampal CCL2 levels at this timepoint and greatly attenuated inflammatory monocyte infiltration at 24 h after inoculation.

Finally, we have recently determined that the size of the inflammatory monocyte response during acute TMEV encephalitis effectively controls the extent of hippocampal injury, the loss of spatial learning, and the induction of high-grade Racine score behavioral seizures in B6 mice (Howe et al., 2022). In this work, we used different amounts of initial TMEV inoculum to drive different levels of encephalitis. We found that introducing 12,500 plaque-forming units of TMEV into the brain elicited encephalitis at 24 h, which was 90% less intense than our standard inoculum of 200,000 plaque-forming units in terms of absolute numbers of infiltrating inflammatory monocytes. While at first glance this seems obvious, it is critical to note that at 24 h the total load of infectious virus in the brain was equivalent between the two inocula. This means that the initial viral exposure, not the amount of replicated virus, set the pace for the downstream encephalitic response. Indeed, animals inoculated with the lower amount of virus exhibited essentially no increase in CCL2 at 24 h and exhibited no increase in TNFα or IL6 in the hippocampus at 24 or 72 h after inoculation. These mice had limited hippocampal injury and showed complete preservation of spatial learning in the Barnes maze. Assessment of behavioral seizures through the first 10 days after inoculation revealed that the low virus group exhibited no high-level Racine seizures at any timepoint. EEG analysis confirmed reduced ictal activity. Notably, however, the low virus group still developed low-grade Racine seizures and did exhibit EEG abnormalities. Looking at microglial activation in these animals, we found that there was equivalent upregulation of Iba-1^+^ microglia in the hippocampus between viral inocula and these microglia showed equivalent upregulation of activation markers such as CD44. Within this context, we also found that mice inoculated with the lower amount of TMEV were more resistant to kainic acid-induced status epilepticus at 24 h relative to mice receiving the standard inoculum, but were still more sensitive than uninfected mice. These findings suggest that microglial activation acts as a binary switch during acute CNS viral infection while the infiltrating monocyte response (and encephalitis, *stricto sensu*) is graded. Moreover, while microglial activation during CNS viral infection primes the brain for ictogenesis, the full induction of acute clinical-grade seizures requires infiltration of inflammatory monocytes. This may have profound implications for considerations of ictogenesis during viral encephalitis. For example, even a small amount of viral invasion into the CNS may trigger microglial activation that confers a decrease in seizure threshold without rising to the level of clinical manifestations. In the context of a patient with other factors predisposing to ictogenesis, this microglial effect may be sufficient to push the system over into a seizure state. Likewise, the critical role of inflammatory monocyte infiltration in driving seizures during viral encephalitis provides an opportunity to consider therapeutic approaches that prevent these cells from invading the CNS. In numerous experiments over many years, we have never observed a detrimental effect on viral clearance associated with suppression of inflammatory monocyte responses, while we have repeatedly observed neuroprotective effects of reducing inflammatory monocyte infiltration. This leads us to strongly favor the development of new therapies to inhibit these cells or the application of unconventional therapies such as monocyte adsorption apheresis in patients with viral encephalitis.

### The Potential Role of Invading Lymphocytes in the Mechanisms Underlying Seizure Generation in the Theiler’s Murine Encephalomyelitis Virus Model

Following the acute innate response to intracerebral inoculation with TMEV, a robust adaptive response is mounted, leading to infiltration of virus-specific CD8^+^ cytotoxic T lymphocytes, which play a significant role in viral clearance from the host (Libbey and Fujinami, 2011). In B6 mice, the earliest wave of anti-viral T cells arrives in the brain around 4 days after inoculation, peaking around day 7 (Deb and Howe, 2008). This response is marked by nearly complete restriction to recognition of a peptide derived from the VP2 capsid protein presented on the D^b^ MHC class I molecule (Johnson et al., 1999; Howe et al., 2007). While antiviral CD8^+^ T cells recognize and kill infected cells, within the CNS it is vital to host survival and function to clear virus non-lytically *via* mechanisms such as IFNγ (Rodriguez et al., 2003). The involvement of CD8^+^ cytotoxic T lymphocytes and viral clearance in the development of acute seizures in the TMEV-induced seizure model was assessed through the use of OT-I transgenic mice (B6 background), in which the majority of the CD8^+^ T cells carry an ovalbumin-specific T-cell receptor (Kirkman et al., 2010). The number of TMEV-infected OT-I mice experiencing acute seizures was comparable to wild-type B6 mice, suggesting that the seizures were not influenced by TMEV-specific CD8^+^ T cells. In TMEV-infected B6 mice, the acute symptomatic seizures resolve by ∼day 10 post-infection (Figure 6B), which was also observed in OT-I mice, indicating that the cessation of seizures was also not due to the clearance of virus by the CD8^+^ T-cell response (Kirkman et al., 2010). Similarly, in our studies, we found no significant correlation (by ROC analysis) between T lymphocyte brain infiltration and acute symptomatic seizures (Bröer et al., 2016). More recently, RAG1^–/–^ mice, which are deficient in mature T and B cells, were compared with B6 mice infected with TMEV (DePaula-Silva et al., 2018). As expected, CD4^+^ and CD8^+^ T cells were absent from the brains of RAG1^–/–^ mice, but the number of RAG1^–/–^ mice experiencing seizures was similar to control mice, further substantiating that lymphocytes are not playing a role in the development of acute seizures following TMEV infection (DePaula-Silva et al., 2018).

When we depleted microglia by prolonged treatment with PLX5662 from 21 days before to 6 or 7 days after TMEV infection of B6 mice, an unfavorable hippocampal and spinal cord ratio between Tregs and effector T cells was observed, thus reducing antiviral immunity in these regions (Waltl et al., 2018b). This possibility was substantiated by a marked increase in brain mRNA expression of the immunosuppressive cytokine IL-10 in the brain of infected PLX5622-treated mice, which is released by Tregs and suppresses the activation of cytotoxic T cells. These data thus added to the concept of microglia–T cell crosstalk (Schetters et al., 2017). Recently, it has been proposed that a dysregulated microglia-T-cell interplay during viral infection may result in altered phagocytosis of neuronal synapses by microglia that causes neurocognitive impairment (Chhatbar and Prinz, 2021).

### The Potential Role of the Excitatory Neurotransmitter Glutamate in the Mechanisms Underlying Seizure Generation in the Theiler’s Murine Encephalomyelitis Virus Model

The role of glutamate receptors and transporters in the TMEV model in B6 mice was studied by the University of Utah groups. Based on previous data from these groups indicating that the inflammatory cytokines IL-6 and TNF-α play a role in seizure development in the TMEV model and that infiltrating monocytes are major producers of these cytokines, the potential role of the metabotropic glutamate receptor 5 (mGluR5) was examined (Hanak et al., 2019). mGluR5 is a G-protein coupled receptor that has been shown to reduce IL-6 and TNF-α production in microglia and macrophages and to provide neuroprotection in other disease models (Loane et al., 2014; Zhang et al., 2015). Hanak et al. (2019) found that pharmacological stimulation of mGluR5 with the selective positive allosteric modulator VU0360172 not only reduced acute seizure outcomes in TMEV-infected B6 mice but also reduced the percent of microglia and macrophages producing TNF-α 3 days post-infection. Immunofluorescence confocal imaging showed a significant decrease in mGluR5 immunoreactivity in the CA1 and CA3 regions of the hippocampus with no significant changes seen in the dentate or cerebral cortex (control brain region) in TMEV-infected B6 mice with seizures compared to controls (Hanak et al., 2019).

Concerning ionotropic glutamate receptors, i.e., NMDA, kainate, and AMPA receptors, Libbey et al. (2016) determined the effects of three antagonists, MK-801, GYKI-52466, and NBQX, on acute seizure development in the TMEV-induced seizure model in B6 mice. Surprisingly, they found that only the AMPA receptor antagonist NBQX affected acute seizure development, resulting in a significantly higher number of mice experiencing seizures, an increase in the number of seizures per mouse, a greater cumulative seizure score per mouse, and a significantly higher mortality rate among the mice. This proconvulsant effect of NBQX observed in the TMEV-induced seizure model was unexpected, because NBQX has previously been shown to be a potent anticonvulsant in a variety of animal seizure models (Catarzi et al., 2007).

In another study, the role of glutamate transporters was examined (Loewen et al., 2019). Glutamate transporters such as GLT-1 expressed by glial cells contribute significantly to the control of extracellular glutamate levels, and the expression profile and function of these glutamate transporters have been implicated in epilepsy (Peterson and Binder, 2020). TMEV-infected seizing B6 mice show evidence of reactive astrogliosis, which has been associated with decreases in glutamate transporter expression and function in sclerotic tissue (Proper et al., 2002; Dossi et al., 2018). However, pharmacological and genetic methods used to modulate the glial glutamate transporters, while effective in other models, were not sufficient to reduce the number or severity of behavioral seizures in TMEV-infected B6 mice (Loewen et al., 2019).

Overall, the TMEV encephalitis model of acute and late seizures in B6 mice is a powerful tool for testing the inflammatory mechanisms that drive ictogenesis. In addition to providing a robust platform for manipulating the host immune response during viral encephalitis to alter seizure biology, the TMEV model also serves as a new, biologically and clinically relevant platform for testing established and novel therapeutics in the context of seizures that evolve without the introduction of ictogenic pharmacological agents or electrical stimulation (Metcalf et al., 2021). While a tremendous amount of progress has been made in understanding the acute phase of TMEV encephalitis, much work remains to discover the cellular and molecular mechanisms that link inflammation to ictogenesis and, critically, to identify the mechanisms that lead from the acute phase of the disease to the development of late, spontaneous seizures (DePaula-Silva et al., 2021).

## Therapeutic Interventions That Interfere With Ictogenesis and Epileptogenesis After Viral Infections

Both the early (acute symptomatic) seizures and the late (spontaneous recurrent) seizures occurring in the acute and chronic phases of the infection, respectively (Figure 4), can be symptomatically suppressed in many patients using antiseizure medications (ASMs) such as levetiracetam, phenytoin, and others (Löscher and Klein, 2021a). More than 30 ASMs are clinically approved; adequate choice of treatment depends on a variety of factors, including the type of seizures and epilepsy (Löscher and Klein, 2021a). However, about 30% of patients with epilepsy do not respond adequately to ASM treatment (Janmohamed et al., 2020), indicating that more research is required to identify the multiple mechanisms driving seizures associated with viral encephalitis and other brain insults.

### Treatment of Acute Symptomatic Seizures

In principle, the use of ASMs does not differ between early and late seizures, but control of acute symptomatic seizures during viral infection requires simultaneous treatment of the underlying etiology (Koppel, 2009; Gunawardane and Fields, 2018; Löscher and Klein, 2021a). Preferred medications for the treatment of acute symptomatic seizures or status epilepticus are those available for intravenous use, such as benzodiazepines, fosphenytoin or phenytoin, valproate, levetiracetam, and phenobarbital (Gunawardane and Fields, 2018). Prophylactic treatment with ASMs should be started as soon as possible after the onset of infection and continued as long as needed (Singhi, 2011). If not adequately treated, early seizures may progress to status epilepticus. However, prophylactic ASMs are not administered in all cases after onset of infection. Instead, ASMs are often withheld until the first-post-infection seizure.

Although the use of ASMs after the onset of infection appears clinically plausible, there is insufficient evidence to support or refute the routine use of ASMs for the prevention of early seizures in viral encephalitis. Concerning other causes of early seizures, only a few placebo-controlled clinical studies on the treatment of early seizures exist. In a study in which treatment with intravenous phenytoin or placebo was started within 24 h of traumatic brain injury, 3.6% of the patients assigned to phenytoin had seizures between the onset of treatment and day 7, as compared with 14.2% of patients assigned to placebo (*P* < 0.0001; Temkin et al., 1990). Although phenytoin is the standard of care to prevent acute symptomatic seizures after brain injury, a recent meta-analysis of clinical studies indicated that levetiracetam has similar efficacy to phenytoin in preventing such seizures (Zhao et al., 2018).

Concerning the treatment of febrile seizures in children, most febrile seizures are self-limited (simple febrile seizures); however, when seizures last longer than 5 min (complex febrile seizures or fSE), a benzodiazepine should be administered to break the seizure (Löscher and Klein, 2021a). A 2018 Cochrane review concluded that intravenous lorazepam and diazepam have similar rates of seizure cessation and respiratory depression (McTague et al., 2018). When intravenous access is unavailable, buccal midazolam or rectal diazepam is acceptable.

In contrast to febrile seizures, FIRES is very difficult to treat. Treatment modalities for these patients include, among others, various ASMs, ketogenic diet, intravenous corticosteroids, intravenous immunoglobulin, and burst-suppression coma (Hon et al., 2018). More recently, based on our initial report of efficacy in a child with FIRES (Kenney-Jung et al., 2016; Clarkson et al., 2019), the IL-1R antagonist anakinra has been increasingly used in the treatment of these patients, with mixed results (Farias-Moeller et al., 2018; Löscher and Klein, 2021a; Yamanaka et al., 2021). Furthermore, the IL-6 receptor antagonist tocilizumab has been used as an alternative (Stredny et al., 2020; Yamanaka et al., 2021).

In the TMEV model in B6 mice, the efficacy of various ASMs and anti-inflammatory compounds to suppress acute symptomatic seizures has been tested. In studies by Barker-Haliski et al. (2015, 2016), valproate, but not carbamazepine or minocycline, reduced the acute seizure burden. In a recent study by Metcalf et al. (2021), several prototype ASMs were effective, including lacosamide, phenytoin, ezogabine, phenobarbital, tiagabine, gabapentin, levetiracetam, topiramate, and valproate. Of these, phenobarbital and valproate had the greatest effect (>95% seizure burden reduction). The prototype anti-inflammatory drugs celecoxib, dexamethasone, and prednisone also moderately reduced seizure burden. Furthermore, cannabidiol reduced seizures in the TMEV model (Patel et al., 2019). The TMEV model in B6 mice is currently utilized by the NIH/NINDS-funded Epilepsy Therapy Screening Program (ETSP) as a tool for evaluating the antiseizure effect of novel compounds (Wilcox et al., 2020).

However, it is important to note that transient treatment of acute symptomatic seizures with ASMs has no proven effect on the subsequent development of epilepsy (Löscher and Klein, 2021a). Similarly, it is not entirely clear whether treatment of FIRES with anakinra improves the long-term prognosis of affected patients (Yamanaka et al., 2021), though several studies report adequate seizure control with ASMs as long as anakinra therapy is maintained (Kenney-Jung et al., 2016; Yamanaka et al., 2021).

### Prevention of Epilepsy and Other Neurological Alterations After Viral Infections

Prevention of epilepsy after neurological insults such as viral encephalitis is an unmet clinical need (Pitkänen and Lukasiuk, 2011; Löscher et al., 2013; Löscher and Klein, 2021a). In principle, there are at least three strategies to prevent epilepsy after brain infections or other epileptogenic brain insults (Vezzani et al., 2016), (1) prevention of the initial insult; (2) initial insult modification (diminishing the long-term consequences of the insult by reducing the severity or duration of the initial brain insult); and (3) “true” antiepileptogenesis or disease modification after the insult by interfering with the mechanisms underlying epileptogenesis. Ad 1, wearing masks in public, maintaining a safe distance from others, and getting vaccinated are examples of prevention of the initial insult, i.e., the infection. Ad 2, intervention with appropriate treatment of the CNS infection that modifies the initial insult and thereby reduces the risk of long-term consequences. A novel emerging strategy is targeting phosphatidylserine receptors such as the TAM tyrosine kinase receptor family (TYRO3, AXL, and MERTK), which confers potent protection against neuroinvasion and CNS lesion development during neuroinvasive virus infection (Wang et al., 2021b). Our findings showing preservation of the hippocampus and inhibition of ictogenesis in mice treated with a calpain inhibitor during the acute phase of TMEV encephalitis is another potential example (Howe et al., 2016). Ad 3, antiepileptogenesis or disease modification after the infection includes treatments that directly target the complex mechanisms underlying epileptogenesis and/or actively repair damaged neural circuits.

As discussed above, the available experimental evidence supports the idea that inflammation in the brain caused by viral infections may contribute to acute seizures and epilepsy development using partially overlapping mechanisms. This highlights the possibility of identifying common targets for therapeutic interventions which may not only suppress the symptoms of the disease but also interfere with key pathogenic mechanisms. Thus, one can envisage the use of specific anti-inflammatory drugs blocking the key pathogenic inflammatory mechanisms (Vezzani, 2014, 2015). The advantage of this approach is that some of these drugs are already available in the clinic for the treatment of auto-inflammatory or autoimmune diseases, such as the IL-1R antagonist anakinra, the IL-1α antagonist canakinumab, the IL-6 receptor antagonist tocilizumab, and the TNF-α antibody infliximab or the TNFα receptor fusion protein etanercept. Preclinical research is important to determine the therapeutic potential of such novel treatments. The challenge, however, is to design an intervention that blocks the detrimental arm of brain inflammation without interfering with the homeostatic mechanisms; in this context, the implementation of resolving anti-inflammatory mechanisms rather than the prevention of the inflammatory cascade may be a better strategy to avoid complications surrounding viral control and clearance (Vezzani et al., 2016). Preventing inflammation may also be difficult due to the rapid onset and amplification of the pathogenic cascade after the first inciting event.

Animal models are important in the search for novel treatments that provide disease-modifying efficacy after viral infections. In a study by Barker-Haliski et al. (2016), in which TMEV-infected B6 mice were treated during the acute phase with minocycline to suppress microglial activation and overexpression of inflammatory cytokines, minocycline improved long-term behavioral outcomes and normalized seizure threshold. However, because late spontaneous seizures are relatively rare in the TMEV model (Figure 6B), necessitating continuous (24/7) video-EEG monitoring of large groups of infected mice, the potential effect of minocycline on the development of epilepsy was not examined by Barker-Haliski et al. (2016). As discussed above, we used continuous video-EEG monitoring in the TMEV model in B6 mice to determine the potential antiepileptogenic effect of pharmacologic and genetic manipulations interfering with monocyte invasion and found no significant effects on the development of epilepsy (Käufer et al., 2018b; Waltl et al., 2018c). The disparity between this finding and evidence that inflammatory monocytes contribute to early ictogenesis further underscores the need for more extensive research in this arena.

Febrile seizures in children often, but not always, occur in the context of an ongoing systemic virus or bacterial infection (Vezzani et al., 2016). This clinical setting has been reproduced in immature rodents by systemic administration of lipopolysaccharide (LPS) to mimic Gram-negative infections or poly(I:C) to mimic viral infections. This acute challenge imposed in a specific developmental window (postnatal day 7–14) increases the susceptibility to subsequent seizures induced by kainate, pilocarpine, or pentylenetetrazole (Galic et al., 2009; Riazi et al., 2010; Galic et al., 2012). The increased susceptibility to provoked seizures was maintained in the animals until adulthood. Systemic inflammation, not the fever *per se*, transiently induced IL-1α and TNF-α in the hippocampus and neocortex, and prevention of this brain response with minocycline, or using an IL-1R antagonist (anakinra) or TNF-α inactivating antibodies, precluded both the acute and long-term reduction in seizure threshold, as well as the comorbidities (anxiety-like behavior and learning and memory deficits) observed in adulthood (Riazi et al., 2010; Galic et al., 2012). The transient inflammatory challenge permanently altered the expression of glutamate receptor subtypes and the Na-K-2Cl co-transporter NKCC1 in the rat forebrain, which may have implications for the observed long-term pathophysiological outcomes (Reid et al., 2013; Riazi et al., 2015).

Interestingly, transient treatment with anakinra after brain injury has been reported to provide disease-modifying or antiepileptogenic effects in non-infectious post-status epilepticus models of acquired epilepsy (Noé et al., 2013; Dyomina et al., 2020). Similarly, the immunomodulator fingolimod, which is established as an MS therapy, was reported to exert antiepileptogenic, neuroprotective effects, and anti-inflammatory effects in post-status epilepticus models of TLE (Gao et al., 2012; Pitsch et al., 2019). The main pharmacologic effect of fingolimod is immunomodulation of lymphocyte homing, thereby reducing the numbers of T and B cells in circulation and, as a consequence, reducing lymphocyte migration into the CNS (Chun et al., 2019). In addition, fingolimod acts on CNS resident cells and inhibits the activation of astrocytes and microglia (Bascunana et al., 2020). Thus, this drug may be an interesting candidate for epilepsy prevention studies in viral encephalitis models, including the TMEV model.

However, as shown in Figure 4, epileptogenesis is a complex multifactorial process, so it seems unlikely that affecting neuroinflammation alone will be sufficient to halt this process. We have proposed previously that multi-targeted cocktails of drugs may provide a more effective strategy for epilepsy prevention after brain insults (Löscher, 2021; Löscher and Klein, 2021b). Proof of concept of this strategy has been achieved for the intrahippocampal kainate mouse model of TLE (Schidlitzki et al., 2020; Welzel et al., 2021). However, it remains to be evaluated whether this strategy is also viable for epilepsy developing during viral infections.

## Conclusion

Robust evidence suggests that ictogenesis during acute viral encephalitis is a complex pathophysiological process that is more than just the sum of parts such as neuroinflammation, invasion of peripheral immune effectors, inflammation- and virus-mediated dysregulation of neural circuitry, and lytic and non-lytic neural cell death. In the absence of complete and global viral eradication, the CNS will always be susceptible to the acutely devastating effects of viral infection. We have provided a broad overview of the manifold viruses that may, directly and indirectly, impact the CNS and we have offered specific mechanistic profiles that offer valuable insights into the potential use of currently available therapeutic strategies to reduce the burden of seizures associated with viral encephalitis. Vitally, we have also offered examples of mechanisms, cell-cell interactions, and host-pathogen interactions that may guide the development of novel therapeutic approaches to preventing, halting, or controlling seizures and the development of epilepsy–not only in patients with viral encephalitis but potentially in many patients with epilepsy and seizure disorders that are refractory to current treatments.

## Author Contributions

Both authors wrote the manuscript and have approved the submission of this work.

## Conflict of Interest

The authors declare that the research was conducted in the absence of any commercial or financial relationships that could be construed as a potential conflict of interest.

## Publisher’s Note

All claims expressed in this article are solely those of the authors and do not necessarily represent those of their affiliated organizations, or those of the publisher, the editors and the reviewers. Any product that may be evaluated in this article, or claim that may be made by its manufacturer, is not guaranteed or endorsed by the publisher.

## References

[B1] Abdullahi A. M. Sarmast S. T. Singh R. (2020). Molecular Biology and Epidemiology of Neurotropic Viruses. *Cureus* 12:e9674. 10.7759/cureus.9674 32923269 PMC7485989

[B2] Anjum S. M. M. Käufer C. Hopfengärtner R. Waltl I. Bröer S. Löscher W. (2018). Automated quantification of EEG spikes and spike clusters as a new read out in Theiler’s virus mouse model of encephalitis-induced epilepsy. *Epilepsy Behav.* 88 189–204. 10.1016/j.yebeh.2018.09.016 30292054

[B3] Annegers J. F. Hauser W. A. Beghi E. Nicolosi A. Kurland L. T. (1988). The risk of unprovoked seizures after encephalitis and meningitis. *Neurology* 38 1407–1410. 10.1212/wnl.38.9.1407 3412588

[B4] Asadi-Pooya A. A. (2020). Seizures associated with coronavirus infections. *Seizure* 79 49–52. 10.1016/j.seizure.2020.05.005 32416567 PMC7212943

[B5] Asadi-Pooya A. A. Simani L. Shahisavandi M. Barzegar Z. (2021). COVID-19, de novo seizures, and epilepsy: a systematic review. *Neurol. Sci.* 42 415–431. 10.1007/s10072-020-04932-2 33237493 PMC7686454

[B6] Ashhurst T. M. Vreden C. Munoz-Erazo L. Niewold P. Watabe K. Terry R. L. (2013). Antiviral macrophage responses in flavivirus encephalitis. *Indian J. Med. Res.* 138 632–647. 24434318 PMC3928696

[B7] Ashraf U. Ding Z. Deng S. Ye J. Cao S. Chen Z. (2021). Pathogenicity and virulence of Japanese encephalitis virus: neuroinflammation and neuronal cell damage. *Virulence* 12 968–980. 10.1080/21505594.2021.1899674 33724154 PMC7971234

[B8] Atluri V. S. Hidalgo M. Samikkannu T. Kurapati K. R. Nair M. (2015). Synaptic Plasticity and Neurological Disorders in Neurotropic Viral Infections. *Neural. Plast.* 2015:138979. 10.1155/2015/138979 26649202 PMC4663354

[B9] Ayuso M. I Martinez-Alonso E. Cid C. Alonso D. L. Alcazar A. (2013). The translational repressor eIF4E-binding protein 2 (4E-BP2) correlates with selective delayed neuronal death after ischemia. *J. Cereb. Blood Flow Metab.* 33 1173–1181. 10.1038/jcbfm.2013.60 23591646 PMC3734765

[B10] Bachiller S. Jiménez-Ferrer I. Paulus A. Yang Y. Swanberg M. Deierborg T. (2018). Microglia in Neurological Diseases: a Road Map to Brain-Disease Dependent-Inflammatory Response. *Front. Cell Neurosci.* 12:488. 10.3389/fncel.2018.00488 30618635 PMC6305407

[B11] Barker-Haliski M. L. Dahle E. J. Heck T. D. Pruess T. H. Vanegas F. Wilcox K. S. (2015). Evaluating an etiologically relevant platform for therapy development for temporal lobe epilepsy: effects of carbamazepine and valproic acid on acute seizures and chronic behavioral comorbidities in the Theiler’s murine encephalomyelitis virus mouse model. *J. Pharmacol. Exp. Ther.* 353 318–329. 10.1124/jpet.114.222513 25755209 PMC4407718

[B12] Barker-Haliski M. L. Heck T. D. Dahle E. J. Vanegas F. Pruess T. H. Wilcox K. S. (2016). Acute treatment with minocycline, but not valproic acid, improves long-term behavioral outcomes in the Theiler’s virus model of temporal lobe epilepsy. *Epilepsia* 57 1958–1967. 10.1111/epi.13577 27739576 PMC5154893

[B13] Barnard C. Wirrell E. (1999). Does status epilepticus in children cause developmental deterioration and exacerbation of epilepsy? *J. Child Neurol.* 14 787–794. 10.1177/088307389901401204 10614565

[B14] Bartlett M. L. Griffin D. E. (2020). Acute RNA Viral Encephalomyelitis and the Role of Antibodies in the Central Nervous System. *Viruses* 2020:12. 10.3390/v12090988 32899509 PMC7551998

[B15] Bartolini L. Piras E. Sullivan K. Gillen S. Bumbut A. Lin C. M. (2018). Detection of HHV-6 and EBV and Cytokine Levels in Saliva From Children With Seizures: results of a Multi-Center Cross-Sectional Study. *Front. Neurol.* 9:834. 10.3389/fneur.2018.00834 30344507 PMC6182262

[B16] Bartolini L. Theodore W. H. Jacobson S. Gaillard W. D. (2019). Infection with HHV-6 and its role in epilepsy. *Epilepsy Res.* 153 34–39. 10.1016/j.eplepsyres.2019.03.016 30953871

[B17] Bascunana P. Möhle L. Brackhan M. Pahnke J. (2020). Fingolimod as a Treatment in Neurologic Disorders Beyond Multiple Sclerosis. *Drugs R. D.* 20 197–207. 10.1007/s40268-020-00316-1 32696271 PMC7419396

[B18] Bennett J. L. Elhofy A. Charo I. Miller S. D. Dal Canto M. C. Karpus W. J. (2007). CCR2 regulates development of Theiler’s murine encephalomyelitis virus-induced demyelinating disease. *Viral. Immunol.* 20 19–33. 10.1089/vim.2006.0068 17425418

[B19] Bennett M. L. Bennett F. C. Liddelow S. A. Ajami B. Zamanian J. L. Fernhoff N. B. (2016). New tools for studying microglia in the mouse and human CNS. *Proc. Natl. Acad. Sci. U. S. A* 113 E1738–E1746. 10.1073/pnas.1525528113 26884166 PMC4812770

[B20] Bissel S. J. Auer R. N. Chiang C. H. Kofler J. Murdoch G. H. Nix W. A. (2015). Human Parechovirus 3 Meningitis and Fatal Leukoencephalopathy. *J. Neuropathol. Exp. Neurol.* 74 767–777. 10.1097/NEN.0000000000000215 26115191

[B21] Blümcke I. Thom M. Wiestler O. D. (2002). Ammon’s horn sclerosis: a maldevelopmental disorder associated with temporal lobe epilepsy. *Brain Pathol.* 12 199–211. 10.1111/j.1750-3639.2002.tb00436.x 11958375 PMC8095862

[B22] Bodro M. Compta Y. Sanchez-Valle R. (2021). Presentations and mechanisms of CNS disorders related to COVID-19. *Neurol. Neuroimmunol. Neuroinflamm.* 2021:8. 10.1212/NXI.0000000000000923 33310765 PMC7808129

[B23] Briese T. Glass W. G. Lipkin W. I. (2000). Detection of West Nile virus sequences in cerebrospinal fluid. *Lancet* 355 1614–1615. 10.1016/s0140-6736(00)02220-0 10821368

[B24] Bröer S. Hage E. Käufer C. Gerhauser I. Anjum M. Li L. (2017). Viral mouse models of multiple sclerosis and epilepsy: Marked differences in neuropathogenesis following infection with two naturally occurring variants of Theiler’s virus BeAn strain. *Neurobiol. Dis.* 99 121–132. 10.1016/j.nbd.2016.12.020 28017800

[B25] Bröer S. Kaufer C. Haist V. Li L. Gerhauser I. Anjum M. (2016). Brain inflammation, neurodegeneration and seizure development following picornavirus infection markedly differ among virus and mouse strains and substrains. *Exp. Neurol.* 279 57–74. 10.1016/j.expneurol.2016.02.011 26892877

[B26] Buenz E. J. Howe C. L. (2006). Picornaviruses and cell death. *Trends Microbiol.* 14 28–36. 10.1016/j.tim.2005.11.003 16337385

[B27] Buenz E. J. Rodriguez M. Howe C. L. (2006). Disrupted spatial memory is a consequence of picornavirus infection. *Neurobiol. Dis.* 24 266–273. 10.1016/j.nbd.2006.07.003 16919964

[B28] Buenz E. J. Sauer B. M. Lafrance-Corey R. G. Deb C. Denic A. German C. L. (2009). Apoptosis of hippocampal pyramidal neurons is virus independent in a mouse model of acute neurovirulent picornavirus infection. *Am. J Pathol.* 175 668–684. 10.2353/ajpath.2009.081126 19608874 PMC2716965

[B29] Buffolo F. Petrosino V. Albini M. Moschetta M. Carlini F. Floss T. (2021). Neuroinflammation induces synaptic scaling through IL-1Î^2^-mediated activation of the transcriptional repressor REST/NRSF. *Cell Death. Dis.* 12:180. 10.1038/s41419-021-03465-6 PMC7884694 33589593

[B30] Butovsky O. Weiner H. L. (2018). Microglial signatures and their role in health and disease. *Nat. Rev. Neurosci.* 19 622–635. 10.1038/s41583-018-0057-5 30206328 PMC7255106

[B31] Büttner C. Heer M. Traichel J. Schwemmle M. Heimrich B. (2019). Zika Virus-Mediated Death of Hippocampal Neurons Is Independent From Maturation State. *Front. Cell Neurosci.* 13:389. 10.3389/fncel.2019.00389 31551711 PMC6736629

[B32] Cabrerizo M. Trallero G. Pena M. J. Cilla A. Megias G. Munoz-Almagro C. (2015). Comparison of epidemiology and clinical characteristics of infections by human parechovirus vs. those by enterovirus during the first month of life. *Eur. J Pediatr.* 174 1511–1516. 10.1007/s00431-015-2566-9 25982340 PMC4623089

[B33] Cain M. D. Salimi H. Diamond M. S. Klein R. S. (2019). Mechanisms of Pathogen Invasion into the Central Nervous System. *Neuron* 103 771–783. 10.1016/j.neuron.2019.07.015 31487528

[B34] Carey R. A. B. Chandiraseharan V. K. Jasper A. Sebastian T. Gujjarlamudi C. Sathyendra S. (2017). Varicella Zoster Virus Infection of the Central Nervous System - 10 Year Experience from a Tertiary Hospital in South India. *Ann. Indian Acad. Neurol.* 20 149–152. 10.4103/aian.AIAN_484_16 28615901 PMC5470156

[B35] Carman K. B. Calik M. Karal Y. Isikay S. Kocak O. Ozcelik A. (2019). Viral etiological causes of febrile seizures for respiratory pathogens (EFES Study). *Hum. Vaccin. Immunother.* 15 496–502. 10.1080/21645515.2018.1526588 30235060 PMC6422444

[B36] Carrera J. P. Forrester N. Wang E. Vittor A. Y. Haddow A. D. Lopez-Verges S. (2013). Eastern equine encephalitis in Latin America. *N. Engl. J Med.* 369 732–744. 10.1056/NEJMoa1212628 23964935 PMC3839813

[B37] Catarzi D. Colotta V. Varano F. (2007). Competitive AMPA receptor antagonists. *Med. Res. Rev.* 27 239–278. 10.1002/med.20084 16892196

[B38] Chatzikonstantinou A. (2014). Epilepsy and the hippocampus. *Front. Neurol. Neurosci.* 34 121–142. 10.1159/000356435 24777136

[B39] Chen H. Y. Yang C. Y. Hsieh C. Y. Yeh C. Y. Chen C. C. Chen Y. C. (2021). Long-term neurological and healthcare burden of adults with Japanese encephalitis: AÂ nationwideÂ study 2000-2015. *PLoS Negl. Trop. Dis.* 15:e0009703. 10.1371/journal.pntd.0009703 34520457 PMC8486099

[B40] Chen R. Wang K. Yu J. Howard D. French L. Chen Z. (2020). The Spatial and Cell-Type Distribution of SARS-CoV-2 Receptor ACE2 in the Human and Mouse Brains. *Front. Neurol.* 11:573095. 10.3389/fneur.2020.573095 33551947 PMC7855591

[B41] Chen S. P. Huang Y. C. Li W. C. Chiu C. H. Huang C. G. Tsao K. C. (2010). Comparison of clinical features between coxsackievirus A2 and enterovirus 71 during the enterovirus outbreak in Taiwan, 2008: a children’s hospital experience. *J. Microbiol. Immunol. Infect.* 43 99–104. 10.1016/S1684-1182(10)60016-3 20457425

[B42] Chen Z. Zhong D. Li G. (2019). The role of microglia in viral encephalitis: a review. *J. Neuroinflammation.* 16:76. 10.1186/s12974-019-1443-2 30967139 PMC6454758

[B43] Cheeran M. C. Hu S. Sheng W. S. Rashid A. Peterson P. K. Lokensgard J. R. (2005). Differential responses of human brain cells to West Nile virus infection. *J. Neurovirol.* 11, 512–524. 10.1080/13550280500384982 16338745

[B44] Chhatbar C. Prinz M. (2021). The roles of microglia in viral encephalitis: from sensome to therapeutic targeting. *Cell Mol. Immunol.* 18 250–258. 10.1038/s41423-020-00620-5 33437050 PMC7802409

[B45] Chika-Igwenyi N. M. Harrison R. E. Psarra C. Gil-Cuesta J. Gulamhusein M. Onwe E. O. (2021). Early onset of neurological features differentiates two outbreaks of Lassa fever in Ebonyi state, Nigeria during 2017-2018. *PLoS Negl. Trop. Dis.* 15:e0009169. 10.1371/journal.pntd.0009169 33684118 PMC7984835

[B46] Choi C. S. Choi Y. J. Choi U. Y. Han J. W. Jeong D. C. Kim H. H. (2011). Clinical manifestations of CNS infections caused by enterovirus type 71. *Korean J. Pediatr.* 54 11–16. 10.3345/kjp.2011.54.1.11 21359055 PMC3040360

[B47] Chu H. X. Arumugam T. V. Gelderblom M. Magnus T. Drummond G. R. Sobey C. G. (2014). Role of CCR2 in inflammatory conditions of the central nervous system. *J. Cereb. Blood Flow Metab* 34 1425–1429. 10.1038/jcbfm.2014.120 24984897 PMC4158674

[B48] Chun J. Kihara Y. Jonnalagadda D. Blaho V. A. (2019). Fingolimod: lessons learned and new opportunities for treating multiple sclerosis and other disorders. *Annu. Rev. Pharmacol. Toxicol.* 59, 149–170. 10.1146/annurev-pharmtox-010818-021358 30625282 PMC6392001

[B49] Clarkson B. D. S. Lafrance-Corey R. G. Kahoud R. J. Farias-Moeller R. Payne E. T. Howe C. L. (2019). Functional deficiency in endogenous interleukin-1 receptor antagonist in patients with febrile infection-related epilepsy syndrome. *Ann. Neurol.* 85 526–537. 10.1002/ana.25439 30779222 PMC6450741

[B50] Coyne C. B. Kim K. S. Bergelson J. M. (2007). Poliovirus entry into human brain microvascular cells requires receptor-induced activation of SHP-2. *EMBO J.* 26 4016–4028. 10.1038/sj.emboj.7601831 17717529 PMC1994131

[B51] Cummins D. Bennett D. Fisher-Hoch S. P. Farrar B. Machin S. J. McCormick J. B. (1992). Lassa fever encephalopathy: clinical and laboratory findings. *J Trop. Med. Hyg.* 95 197–201. 1597876

[B52] Cusick M. F. Libbey J. E. Doty D. J. DePaula-Silva A. B. Fujinami R. S. (2017). The role of peripheral interleukin-6 in the development of acute seizures following virus encephalitis. *J Neurovirol.* 23 696–703. 10.1007/s13365-017-0554-8 28741149 PMC5656516

[B53] Cusick M. F. Libbey J. E. Fujinami R. S. (2012). Molecular mimicry as a mechanism of autoimmune disease. *Clin. Rev. Allergy Immunol.* 42 102–111. 22095454 10.1007/s12016-011-8294-7 PMC3266166

[B54] Cusick M. F. Libbey J. E. Patel D. C. Doty D. J. Fujinami R. S. (2013). Infiltrating macrophages are key to the development of seizures following virus infection. *J. Virol.* 87 1849–1860. 10.1128/JVI.02747-12 23236075 PMC3554195

[B55] Danielski L. G. Giustina A. D. Badawy M. Barichello T. Quevedo J. Dal Pizzol F. (2018). Brain Barrier Breakdown as a Cause and Consequence of Neuroinflammation in Sepsis. *Mol. Neurobiol.* 55 1045–1053. 10.1007/s12035-016-0356-7 28092082

[B56] Das S. Mishra K. P. Pramanik A. Mishra P. Chanda S. Ganju L. (2021). Re-exposure to alarmin HMGB1 and cytokine IL-1 beta induces differential innate immune response generation in mouse brain. *J. Neuroimmunol.* 357 577625. 10.1016/j.jneuroim.2021.577625 34153804

[B57] Datta G. Miller N. M. Halcrow P. W. Khan N. Colwell T. Geiger J. D. (2021). SARS-CoV-2 S1 Protein Induces Endolysosome Dysfunction and Neuritic Dystrophy. *Front. Cell Neurosci.* 15:777738. 10.3389/fncel.2021.777738 34776872 PMC8579006

[B58] de Graaf H. Pelosi E. Cooper A. Pappachan J. Sykes K. MacIntosh I. (2016). Severe Enterovirus Infections in Hospitalized Children in the South of England: Clinical Phenotypes and Causative Genotypes. *Pediatr. Infect. Dis. J.* 35 723–727. 10.1097/INF.0000000000001093 26882165 PMC4985250

[B59] Deb C. Howe C. L. (2008). NKG2D contributes to efficient clearance of picornavirus from the acutely infected murine brain. *J. Neurovirol.* 14 261–266. 10.1080/13550280802105002 18569460 PMC3181148

[B60] DellaBadia J. Jr. Jaffe S. L. Singh J. Minagar A. (2004). An occipital lobe epileptogenic focus in a patient with West Nile encephalitis. *Eur. J. Neurol.* 11 111–113. 10.1046/j.1351-5101.2003.00726.x 14748771

[B61] Demir C. F. Berilgen M. S. Mungen B. Bulut S. (2012). Do polio survivors have a higher risk of epilepsy? *Epilepsy Res.* 98 72–75. 10.1016/j.eplepsyres.2011.08.019 21996150

[B62] DePaula-Silva A. B. Bell L. A. Wallis G. J. Wilcox K. S. (2021). Inflammation Unleashed in Viral-Induced Epileptogenesis. *Epilepsy Curr.* 21 433–440. 10.1177/15357597211040939 34924851 PMC8652320

[B63] DePaula-Silva A. B. Hanak T. J. Libbey J. E. Fujinami R. S. (2017). Theiler’s murine encephalomyelitis virus infection of SJL/J and C57BL/6J mice: models for multiple sclerosis and epilepsy. *J. Neuroimmunol.* 308 30–42. 10.1016/j.jneuroim.2017.02.012 28237622 PMC5474355

[B64] DePaula-Silva A. B. Sonderegger F. L. Libbey J. E. Doty D. J. Fujinami R. S. (2018). The immune response to picornavirus infection and the effect of immune manipulation on acute seizures. *J. Neurovirol.* 24 464–477. 10.1007/s13365-018-0636-2 29687406 PMC6105529

[B65] Dethlefs S. Brahic M. Larsson-Sciard E. L. (1997). An early, abundant cytotoxic T-lymphocyte response against Theiler’s virus is critical for preventing viral persistence. *J Virol.* 71 8875–8878. 10.1128/JVI.71.11.8875-8878.1997 9343251 PMC192357

[B66] Di Virgilio F. Sarti A. C. Coutinho-Silva R. (2020). Purinergic signaling, DAMPs, and inflammation. *Am. J. Physiol. Cell Physiol.* 318 C832–C835. 10.1152/ajpcell.00053.2020 32159362

[B67] Ding A. Shen B. Elliott S. Joshi K. Coghlin D. McLaughlin S. (2020). Double Crossed: A Case of La Crosse Encephalitis. *R. I. Med. J.* 103 59–62. 32236166

[B68] Ding X. Li S. Zhu L. (2021). Potential effects of HMGB1 on viral replication and virus infection-induced inflammatory responses: a promising therapeutic target for virus infection-induced inflammatory diseases. *Cytokine Growth Factor Rev.* 62 54–61. 10.1016/j.cytogfr.2021.08.003 34503914

[B69] Dossi E. Vasile F. Rouach N. (2018). Human astrocytes in the diseased brain. *Brain Res. Bull.* 136 139–156. 10.1016/j.brainresbull.2017.02.001 28212850 PMC5766741

[B70] Doyle M. F. (2021). Central nervous system outcomes of COVID-19. *Transl. Res.* 241 41–51. 10.1016/j.trsl.2021.09.002 34601116 PMC8482653

[B71] Dube C. Vezzani A. Behrens M. Bartfai T. Baram T. Z. (2005). Interleukin-1beta contributes to the generation of experimental febrile seizures. *Ann. Neurol.* 57 152–155. 10.1002/ana.20358 15622539 PMC2909879

[B72] Dube C. M. Ravizza T. Hamamura M. Zha Q. Keebaugh A. Fok K. (2010). Epileptogenesis provoked by prolonged experimental febrile seizures: mechanisms and biomarkers. *J. Neurosci.* 30 7484–7494. 10.1523/JNEUROSCI.0551-10.2010 20519523 PMC2906240

[B73] Dunn N. Kharlamova N. Fogdell-Hahn A. (2020). The role of herpesvirus 6A and 6B in multiple sclerosis and epilepsy. *Scand. J. Immunol.* 92:e12984. 10.1111/sji.12984 33037649 PMC7757173

[B74] Düsedau H. P. Steffen J. Figueiredo C. A. Boehme J. D. Schultz K. Erck C. (2021). Influenza A Virus (H1N1) Infection Induces Microglial Activation and Temporal Dysbalance in Glutamatergic Synaptic Transmission. *mBio* 12:e0177621. 10.1128/mBio.01776-21 34700379 PMC8546584

[B75] Dyomina A. V. Zubareva O. E. Smolensky I. V. Vasilev D. S. Zakharova M. V. Kovalenko A. A. (2020). Anakinra Reduces Epileptogenesis, Provides Neuroprotection, and Attenuates Behavioral Impairments in Rats in the Lithium-Pilocarpine Model of Epilepsy. *Pharmaceuticals* 2020:13. 10.3390/ph13110340 33113868 PMC7692198

[B76] Ekstrand J. J. Herbener A. Rawlings J. Turney B. Ampofo K. Korgenski E. K. (2010). Heightened neurologic complications in children with pandemic H1N1 influenza. *Ann. Neurol.* 68 762–766. 10.1002/ana.22184 20865762

[B77] Elgamasy S. Kamel M. G. Ghozy S. Khalil A. Morra M. E. Islam S. M. S. (2020). First case of focal epilepsy associated with SARS-coronavirus-2. *J. Med. Virol.* 92 2238–2242. 10.1002/jmv.26113 32484990 PMC7300744

[B78] Engel J. (1996). Introduction to temporal lobe epilepsy. *Epilepsy Res.* 26 141–150. 10.1016/s0920-1211(96)00043-5 8985696

[B79] Epstein L. G. Shinnar S. Hesdorffer D. C. Nordli D. R. Hamidullah A. Benn E. K. (2012). Human herpesvirus 6 and 7 in febrile status epilepticus: the FEBSTAT study. *Epilepsia* 53 1481–1488. 10.1111/j.1528-1167.2012.03542.x 22954016 PMC3442944

[B80] Etchison D. Milburn S. C. Edery I. Sonenberg N. Hershey J. W. (1982). Inhibition of HeLa cell protein synthesis following poliovirus infection correlates with the proteolysis of a 220, 000-dalton polypeptide associated with eucaryotic initiation factor 3 and a cap binding protein complex. *J. Biol. Chem.* 257 14806–14810. 10.1016/s0021-9258(18)33352-0 6294080

[B81] Evans A. B. Winkler C. W. Peterson K. E. (2019). Differences in Neuropathogenesis of Encephalitic California Serogroup Viruses. *Emerg. Infect. Dis.* 25 728–738. 10.3201/eid2504.181016 30882310 PMC6433036

[B82] Farias-Moeller R. LaFrance-Corey R. Bartolini L. Wells E. M. Baker M. Doslea A. (2018). Fueling the FIRES: Hemophagocytic lymphohistiocytosis in febrile infection-related epilepsy syndrome. *Epilepsia* 59 1753–1763. 10.1111/epi.14524 30132834

[B83] Fauci A. S. (2006). Seasonal and pandemic influenza preparedness: science and countermeasures. *J. Infect. Dis.* 194 (Suppl. 2), S73–S76. 10.1086/507550 17163392

[B84] Faust N. Varas F. Kelly L. M. Heck S. Graf T. (2000). Insertion of enhanced green fluorescent protein into the lysozyme gene creates mice with green fluorescent granulocytes and macrophages. *Blood* 96 719–726. 10.1182/blood.v96.2.719 10887140

[B85] Fischer S. A. Graham M. B. Kuehnert M. J. Kotton C. N. Srinivasan A. Marty F. M. (2006). Transmission of lymphocytic choriomeningitis virus by organ transplantation. *N. Engl. J. Med.* 354 2235–2249. 10.1056/NEJMoa053240 16723615

[B86] Fischer T. K. Simmonds P. Harvala H. (2022). The importance of enterovirus surveillance in a post-polio world. *Lancet Infect. Dis.* 22 e35–e40. 34265258 10.1016/S1473-3099(20)30852-5

[B87] Fisher R. S. Acevedo C. Arzimanoglou A. Bogacz A. Cross J. H. Elger C. E. (2014). ILAE official report: a practical clinical definition of epilepsy. *Epilepsia* 55 475–482. 10.1111/epi.12550 24730690

[B88] Flowers S. A. Rebeck G. W. (2020). APOE in the normal brain. *Neurobiol. Dis.* 136 104724. 10.1016/j.nbd.2019.104724 31911114 PMC7002287

[B89] Fotheringham J. Williams E. L. Akhyani N. Jacobson S. (2008). Human herpesvirus 6 (HHV-6) induces dysregulation of glutamate uptake and transporter expression in astrocytes. *J. Neuroimmune. Pharmacol.* 3 105–116. 10.1007/s11481-007-9084-0 18247129

[B90] Fox K. Wells M. E. Tennison M. Vaughn B. (2017). Febrile Infection-Related Epilepsy Syndrome (FIRES): a Literature Review and Case Study. *Neurodiagn. J.* 57 224–233. 10.1080/21646821.2017.1355181 28898171

[B91] Friedman A. Kaufer D. Heinemann U. (2009). Blood-brain barrier breakdown-inducing astrocytic transformation: novel targets for the prevention of epilepsy. *Epilepsy Res.* 85 142–149. 10.1016/j.eplepsyres.2009.03.005 19362806 PMC3615244

[B92] Frontera J. A. Sabadia S. Lalchan R. Fang T. Flusty B. Millar-Vernetti P. (2021). A Prospective Study of Neurologic Disorders in Hospitalized Patients With COVID-19 in New York City. *Neurology* 96 e575–e586. 33020166 10.1212/WNL.0000000000010979 PMC7905791

[B93] Fujii K. Nagata N. Sato Y. Ong K. C. Wong K. T. Yamayoshi S. (2013). Transgenic mouse model for the study of enterovirus 71 neuropathogenesis. *Proc. Natl. Acad. Sci. U. S. A* 110 14753–14758. 10.1073/pnas.1217563110 23959904 PMC3767555

[B94] Galic M. A. Riazi K. Henderson A. K. Tsutsui S. Pittman Q. J. (2009). Viral-like brain inflammation during development causes increased seizure susceptibility in adult rats. *Neurobiol. Dis.* 36 343–351. 10.1016/j.nbd.2009.07.025 19660546 PMC3526656

[B95] Galic M. A. Riazi K. Pittman Q. J. (2012). Cytokines and brain excitability. *Front. Neuroendocrinol.* 33 116–125. 10.1016/j.yfrne.2011.12.002 22214786 PMC3547977

[B96] Gallentine W. B. Shinnar S. Hesdorffer D. C. Epstein L. Nordli D. R. Jr. Lewis D. V. (2017). Plasma cytokines associated with febrile status epilepticus in children: a potential biomarker for acute hippocampal injury. *Epilepsia* 58 1102–1111. 10.1111/epi.13750 28448686 PMC5482499

[B97] Gao F. Liu Y. Li X. Wang Y. Wei D. Jiang W. (2012). Fingolimod (FTY720) inhibits neuroinflammation and attenuates spontaneous convulsions in lithium-pilocarpine induced status epilepticus in rat model. *Pharmacol. Biochem. Behav.* 103 187–196. 10.1016/j.pbb.2012.08.025 22960129

[B98] Gao Q. Hernandes M. S. (2021). Sepsis-Associated Encephalopathy and Blood-Brain Barrier Dysfunction. *Inflammation* 44 2143–2150. 10.1007/s10753-021-01501-3 34291398 PMC11044530

[B99] Gerhauser I. Hansmann F. Ciurkiewicz M. Löscher W. Beineke A. (2019). Facets of Theiler’s Murine Encephalomyelitis Virus-Induced Diseases: an Update. *Int. J. Mol. Sci.* 2019 20. 10.3390/ijms20020448 30669615 PMC6358740

[B100] Gern O. L. Mulenge F. Pavlou A. Ghita L. Steffen I. Stangel M. (2021). Toll-like Receptors in Viral Encephalitis. *Viruses* 2021:13. 10.3390/v13102065 PMC8540543 34696494

[B101] Getts D. R. Balcar V. J. Matsumoto I. Muller M. King N. J. (2008). Viruses and the immune system: their roles in seizure cascade development. *J. Neurochem.* 104 1167–1176. 10.1111/j.1471-4159.2007.05171.x 18205751

[B102] Getts D. R. Matsumoto I. MÃller M. Getts M. T. Radford J. Shrestha B. (2007). Role of IFN-gamma in an experimental murine model of West Nile virus-induced seizures. *J. Neurochem.* 103 1019–1030. 10.1111/j.1471-4159.2007.04798.x 17854352

[B103] Ghasemi M. Schachter S. C. (2011). The NMDA receptor complex as a therapeutic target in epilepsy: a review. *Epilepsy Behav.* 22 617–640. 10.1016/j.yebeh.2011.07.024 22056342

[B104] Greter M. Lelios I. Croxford A. L. (2015). Microglia Versus Myeloid Cell Nomenclature during Brain Inflammation. *Front. Immunol.* 6:249. 10.3389/fimmu.2015.00249 26074918 PMC4443742

[B105] Griffin D. E. (2010). Recovery from viral encephalomyelitis: immune-mediated noncytolytic virus clearance from neurons. *Immunol. Res.* 47 123–133. 10.1007/s12026-009-8143-4 20087684 PMC2891389

[B106] Griffin D. E. Metcalf T. (2011). Clearance of virus infection from the CNS. *Curr. Opin. Virol.* 1 216–221. 10.1016/j.coviro.2011.05.021 21927638 PMC3171972

[B107] Gromeier M. Solecki D. Patel D. D. Wimmer E. (2000). Expression of the human poliovirus receptor/CD155 gene during development of the central nervous system: implications for the pathogenesis of poliomyelitis. *Virology* 273 248–257. 10.1006/viro.2000.0418 10915595

[B108] Guerrini L. Blasi F. Denis-Donini S. (1995). Synaptic activation of NF-kappa B by glutamate in cerebellar granule neurons in vitro. *Proc. Natl. Acad. Sci. U. S. A* 92 9077–9081. 10.1073/pnas.92.20.9077 7568076 PMC40927

[B109] Gunawardane N. Fields M. (2018). Acute Symptomatic Seizures and Provoked Seizures: to Treat or Not to Treat? *Curr. Treat. Options. Neurol.* 20:41. 10.1007/s11940-018-0525-2 30136002

[B110] Gupta M. Weaver D. F. (2021). COVID-19 as a Trigger of Brain Autoimmunity. *ACS Chem. Neurosci.* 12 2558–2561. 10.1021/acschemneuro.1c00403 34213312

[B111] Habbas S. Santello M. Becker D. Stubbe H. Zappia G. Liaudet N. (2015). Neuroinflammatory TNFα Impairs Memory via Astrocyte Signaling. *Cell* 163 1730–1741. 10.1016/j.cell.2015.11.023 26686654

[B112] Han D. H. Kim S. Y. Lee N. M. Yi D. Y. Yun S. W. Lim I. S. (2019). Seasonal distribution of febrile seizure and the relationship with respiratory and enteric viruses in Korean children based on nationwide registry data. *Seizure* 73 9–13. 10.1016/j.seizure.2019.10.008 31675516 PMC7111037

[B113] Hanada T. (2020). Ionotropic Glutamate Receptors in Epilepsy: a Review Focusing on AMPA and NMDA Receptors. *Biomolecules* 2020:10. 10.3390/biom10030464 32197322 PMC7175173

[B114] Hanak T. J. Libbey J. E. Doty D. J. Sim J. T. DePaula-Silva A. B. Fujinami R. S. (2019). Positive modulation of mGluR5 attenuates seizures and reduces TNF-Î±(+) macrophages and microglia in the brain in a murine model of virus-induced temporal lobe epilepsy. *Exp. Neurol.* 311 194–204. 10.1016/j.expneurol.2018.10.006 30316834 PMC6263825

[B115] Härtel C. Schilling S. Gottschalk S. Sperner J. (2002). Isolated unilateral cortical oedema and complex partial seizures in association with coxsackievirus B infection. *Epilepsy Behav.* 3 480–482. 10.1016/s1525-5050(02)00513-9 12609272

[B116] Haughey N. J. Nath A. Mattson M. P. Slevin J. T. Geiger J. D. (2001). HIV-1 Tat through phosphorylation of NMDA receptors potentiates glutamate excitotoxicity. *J. Neurochem.* 78 457–467. 10.1046/j.1471-4159.2001.00396.x 11483648

[B117] Hazama K. Shiihara T. Tsukagoshi H. Matsushige T. Dowa Y. Watanabe M. (2019). Rhinovirus-associated acute encephalitis/encephalopathy and cerebellitis. *Brain Dev.* 41 551–554. 10.1016/j.braindev.2019.02.014 30850156

[B118] Heida J. G. Pittman Q. J. (2005). Causal links between brain cytokines and experimental febrile convulsions in the rat. *Epilepsia* 46, 1906–1913. 10.1111/j.1528-1167.2005.00294.x 16393156

[B119] Hesdorffer D. C. Shinnar S. Lewis D. V. Moshe S. L. Nordli D. R. Jr. Pellock J. M. (2012). Design and phenomenology of the FEBSTAT study. *Epilepsia* 53 1471–1480. 10.1111/j.1528-1167.2012.03567.x 22742587 PMC3436982

[B120] Hoffmann M. Kleine-Weber H. Schroeder S. Krüger N. Herrler T. Erichsen S. (2020). SARS-CoV-2 cell entry depends on ACE2 and TMPRSS2 and is blocked by a clinically proven protease inhibitor. *Cell* 181, 271–280. 10.1016/j.cell.2020.02.052 32142651 PMC7102627

[B121] Holbrook M. R. Gowen B. B. (2008). Animal models of highly pathogenic RNA viral infections: encephalitis viruses. *Antiviral Res.* 78 69–78. 10.1016/j.antiviral.2007.10.004 18031836

[B122] Hon K. L. Leung A. K. C. Torres A. R. (2018). Febrile Infection-Related Epilepsy Syndrome (FIRES): An Overview of Treatment and Recent Patents. *Recent Pat Inflamm. Allergy Drug Discov.* 12 128–135. 10.2174/1872213X12666180508122450 29745347

[B123] Hosoya M. Sato M. Honzumi K. Katayose M. Kawasaki Y. Sakuma H. (2001). Association of nonpolio enteroviral infection in the central nervous system of children with febrile seizures. *Pediatrics* 107:E12. 10.1542/peds.107.1.e12 11134476

[B124] Hosseini S. Wilk E. Michaelsen-Preusse K. Gerhauser I. Baumgärtner W. Geffers R. (2018). Long-Term Neuroinflammation Induced by Influenza A Virus Infection and the Impact on Hippocampal Neuron Morphology and Function. *J. Neurosci.* 38 3060–3080. 10.1523/JNEUROSCI.1740-17.2018 29487124 PMC6596076

[B125] Howe C. L. Lafrance-Corey R. G. Goddery E. N. Johnson R. K. Mirchia K. (2017). Neuronal CCL2 expression drives inflammatory monocyte infiltration into the brain during acute virus infection. *J. Neuroinflammation.* 14:238. 10.1186/s12974-017-1015-2 29202854 PMC5715496

[B126] Howe C. L. Lafrance-Corey R. G. Mirchia K. Sauer B. M. McGovern R. M. Reid J. M. (2016). Neuroprotection mediated by inhibition of calpain during acute viral encephalitis. *Sci. Rep.* 6:28699. 10.1038/srep28699 27345730 PMC4921808

[B127] Howe C. L. Lafrance-Corey R. G. Overlee B. L. Johnson R. K. Clarkson B. D. S. Goddery E. N. (2022). Inflammatory monocytes and microglia play independent roles in inflammatory ictogenesis. *J. Neuroinflam.* 19:22. 10.1186/s12974-022-02394-1 35093106 PMC8800194

[B128] Howe C. L. Lafrance-Corey R. G. Sundsbak R. S. Lafrance S. J. (2012a). Inflammatory monocytes damage the hippocampus during acute picornavirus infection of the brain. *J. Neuroinflam.* 9:50. 10.1186/1742-2094-9-50 22405261 PMC3368782

[B129] Howe C. L. Lafrance-Corey R. G. Sundsbak R. S. Sauer B. M. Lafrance S. J. Buenz E. J. (2012b). Hippocampal protection in mice with an attenuated inflammatory monocyte response to acute CNS picornavirus infection. *Sci. Rep.* 2:545. 10.1038/srep00545 22848791 PMC3408132

[B130] Howe C. L. Ure D. Adelson J. D. LaFrance-Corey R. Johnson A. Rodriguez M. (2007). CD8+ T cells directed against a viral peptide contribute to loss of motor function by disrupting axonal transport in a viral model of fulminant demyelination. *J. Neuroimmunol.* 188 13–21. 10.1016/j.jneuroim.2007.04.005 17493690 PMC1986839

[B131] Huang C. Yan B. Lei D. Si Y. Li H. Chen M. W. (2015). Apolipoprotein 4 may increase viral load and seizure frequency in mesial temporal lobe epilepsy patients with positive human herpes virus 6B. *Neurosci. Lett.* 593 29–34. 10.1016/j.neulet.2014.12.063 25576704

[B132] Huang H. I. Shih S. R. (2015). Neurotropic Enterovirus Infections in the Central Nervous System. *Viruses* 7 6051–6066. 10.3390/v7112920 26610549 PMC4664993

[B133] Huang Y. Smith D. E. Ibanez-Sandoval O. Sims J. E. Friedman W. J. (2011). Neuron-specific effects of interleukin-1ß are mediated by a novel isoform of the IL-1 receptor accessory protein. *J. Neurosci.* 31 18048–18059. 10.1523/JNEUROSCI.4067-11.2011 22159118 PMC3261076

[B134] Hunter J. M. Cirrito J. R. Restivo J. L. Kinley R. D. Sullivan P. M. Holtzman D. M. (2012). Emergence of a seizure phenotype in aged apolipoprotein epsilon 4 targeted replacement mice. *Brain Res.* 1467 120–132. 10.1016/j.brainres.2012.05.048 22682924

[B135] Jacob F. Pather S. R. Huang W. K. Zhang F. Wong S. Z. H. Zhou H. (2020). Human Pluripotent Stem Cell-Derived Neural Cells and Brain Organoids Reveal SARS-CoV-2 Neurotropism Predominates in Choroid Plexus Epithelium. *Cell Stem Cell* 27 937–950. 10.1016/j.stem.2020.09.016 33010822 PMC7505550

[B136] Janmohamed M. Brodie M. J. Kwan P. (2020). Pharmacoresistance - Epidemiology, mechanisms, and impact on epilepsy treatment. *Neuropharmacology* 168:107790. 10.1016/j.neuropharm.2019.107790 31560910

[B137] Jarrahi A. Ahluwalia M. Khodadadi H. Silva Lopes S. E. Kolhe R. Hess D. C. (2020). Neurological consequences of COVID-19: what have we learned and where do we go from here? *J. Neuroinflam.* 17:286. 10.1186/s12974-020-01957-4 32998763 PMC7525232

[B138] Jeong H. K. Ji K. Min K. Joe E. H. (2013). Brain inflammation and microglia: facts and misconceptions. *Exp. Neurobiol.* 22 59–67. 10.5607/en.2013.22.2.59 23833554 PMC3699675

[B139] Jiang L. Shao Y. Tian Y. Ouyang C. Wang X. (2020). Nuclear Alarmin Cytokines in Inflammation. *J. Immunol. Res.* 2020:7206451. 10.1155/2020/7206451 33344656 PMC7732391

[B140] Jmii H. Halouani A. Maatouk M. Chekir-Ghedira L. Aouni M. Fisson S. (2020). Coxsackievirus B4 infection and interneuronal spread in primary cultured neurons. *Microb. Pathog.* 145:104235. 10.1016/j.micpath.2020.104235 32360191

[B141] Johnson A. J. Njenga M. K. Hansen M. J. Kuhns S. T. Chen L. Rodriguez M. (1999). Prevalent class I-restricted T-cell response to the Theiler’s virus epitope Db:VP2121-130 in the absence of endogenous CD4 help, tumor necrosis factor alpha, gamma interferon, perforin, or costimulation through CD28. *J. Virol.* 73 3702–3708. 10.1128/jvi.73.5.3702-3708.1999 10196262 PMC104145

[B142] Jones E. Pillay T. D. Liu F. Luo L. Bazo-Alvarez J. C. Yuan C. (2018). Outcomes following severe hand foot and mouth disease: a systematic review and meta-analysis. *Eur. J. Paediatr. Neurol.* 22 763–773. 10.1016/j.ejpn.2018.04.007 29778429 PMC6148319

[B143] Joubert B. Dalmau J. (2019). The role of infections in autoimmune encephalitides. *Rev. Neurol.* 175 420–426. 10.1016/j.neurol.2019.07.004 31371185

[B144] Jurgens H. A. Amancherla K. Johnson R. W. (2012). Influenza infection induces neuroinflammation, alters hippocampal neuron morphology, and impairs cognition in adult mice. *J. Neurosci.* 32 3958–3968. 10.1523/JNEUROSCI.6389-11.2012 22442063 PMC3353809

[B145] Kaida A. Kubo H. Sekiguchi J. Kohdera U. Togawa M. Shiomi M. (2011). Enterovirus 68 in children with acute respiratory tract infections, Osaka, Japan. *Emerg. Infect. Dis.* 17 1494–1497. 10.3201/eid1708.110028 21801632 PMC3381549

[B146] Kalinke U. Bechmann I. Detje C. N. (2011). Host strategies against virus entry via the olfactory system. *Virulence* 2 367–370. 10.4161/viru.2.4.16138 21758005

[B147] Kaltschmidt C. Kaltschmidt B. Baeuerle P. A. (1995). Stimulation of ionotropic glutamate receptors activates transcription factor NF-kappa B in primary neurons. *Proc. Natl. Acad. Sci. U. S. A* 92 9618–9622. 10.1073/pnas.92.21.9618 7568184 PMC40853

[B148] Kang S. S. McGavern D. B. (2008). Lymphocytic choriomeningitis infection of the central nervous system. *Front. Biosci.* 13 4529–4543. 10.2741/3021 18508527 PMC5279998

[B149] Kapur M. Monaghan C. E. Ackerman S. L. (2017). Regulation of mRNA Translation in Neurons-A Matter of Life and Death. *Neuron* 96 616–637. 10.1016/j.neuron.2017.09.057 29096076 PMC5693308

[B150] Käufer C. Chatbar C. Bröer S. Waltl I. Luca G. Gerhauser I. (2018a). Chemokine receptors CCR2 and CX3CR1 regulate viral encephalitis-induced hippocampal damage but not seizures. *Proc. Natl. Acad. Sci.* 115 E8929–E8938. 10.1073/pnas.1806754115 30181265 PMC6156634

[B151] Käufer C. Chatbar C. Waltl I. Bröer S. Gerhauser I. Kalinke U. (2018b). “*Ccr2*-KO leads to protection from hippocampal neurodegeneration in Theiler’s virus model of epilepsy,” in *Key Stone Symposium “New Frontiers in Neuroinflammation: What Happens When CNS and Periphery Meet?”, June 17-21 2018* (Keystone, CO).

[B152] Kawamura Y. Nakayama A. Kato T. Miura H. Ishihara N. Ihira M. (2015). Pathogenic Role of Human Herpesvirus 6B Infection in Mesial Temporal Lobe Epilepsy. *J. Infect. Dis.* 212 1014–1021. 10.1093/infdis/jiv160 25840441

[B153] Kellinghaus C. Engbring C. Kovac S. Möddel G. Boesebeck F. Fischera M. (2008). Frequency of seizures and epilepsy in neurological HIV-infected patients. *Seizure* 17 27–33. 10.1016/j.seizure.2007.05.017 17618132

[B154] Kenney-Jung D. L. Vezzani A. Kahoud R. J. Lafrance-Corey R. G. Ho M. L. Muskardin T. W. (2016). Febrile infection-related epilepsy syndrome treated with anakinra. *Ann. Neurol.* 80 939–945. 10.1002/ana.24806 27770579 PMC5225882

[B155] Kierdorf K. Masuda T. Jordao M. J. C. Prinz M. (2019). Macrophages at CNS interfaces: ontogeny and function in health anddisease. *Nat. Rev. Neurosci.* 20 547–562. 10.1038/s41583-019-0201-x 31358892

[B156] Kirkman N. J. Libbey J. E. Wilcox K. S. White H. S. Fujinami R. S. (2010). Innate but not adaptive immune responses contribute to behavioral seizures following viral infection. *Epilepsia* 51 454–464. 10.1111/j.1528-1167.2009.02390.x 19845729 PMC3046460

[B157] Klein P. Dingledine R. Aronica E. Bernard C. Blümcke I. Boison D. (2018). Commonalities in epileptogenic processes from different acute brain insults: do they translate? *Epilepsia* 59 37–66. 10.1111/epi.13965 29247482 PMC5993212

[B158] Klein R. S. Garber C. Funk K. E. Salimi H. Soung A. Kanmogne M. (2019). Neuroinflammation During RNA Viral Infections. *Annu. Rev. Immunol.* 37 73–95. 10.1146/annurev-immunol-042718-041417 31026414 PMC6731125

[B159] Kollmus H. Pilzner C. Leist S. R. Heise M. Geffers R. Schughart K. (2018). Of mice and men: the host response to influenza virus infection. *Mamm. Genome* 29 446–470. 10.1007/s00335-018-9750-y 29947965 PMC6132725

[B160] Koppel B. S. (2009). Treatment of acute and remote symptomatic seizures. *Curr. Treat. Options. Neurol.* 11 231–241. 10.1007/s11940-009-0027-3 19523349

[B161] Koyuncu O. O. Hogue I. B. Enquist L. W. (2013). Virus infections in the nervous system. *Cell Host. Microbe* 13 379–393. 23601101 10.1016/j.chom.2013.03.010 PMC3647473

[B162] Krishnakumar V. Durairajan S. S. K. Alagarasu K. Li M. Dash A. P. (2019). Recent Updates on Mouse Models for Human Immunodeficiency, Influenza, and Dengue Viral Infections. *Viruses* 2019:11. 10.3390/v11030252 30871179 PMC6466164

[B163] Kuo C. L. Pilling L. C. Atkins J. L. Masoli J. A. H. Delgado J. Kuchel G. A. (2020). APOE e4 Genotype Predicts Severe COVID-19 in the UK Biobank Community Cohort. *J. Gerontol. A Biol. Sci. Med. Sci.* 75 2231–2232. 10.1093/gerona/glaa131 32451547 PMC7314139

[B164] Kurd M. Hashavya S. Benenson S. Gilboa T. (2021). Seizures as the main presenting manifestation of acute SARS-CoV-2 infection in children. *Seizure* 92 89–93. 10.1016/j.seizure.2021.08.017 34481322 PMC8397499

[B165] Labzin L. I. Lauterbach M. A. Latz E. (2016). Interferons and inflammasomes: Cooperation and counterregulation in disease. *J Allergy Clin. Immunol.* 138 37–46. 10.1016/j.jaci.2016.05.010 27373324

[B166] Lai F. Chen Q. (2018). Humanized Mouse Models for the Study of Infection and Pathogenesis of Human Viruses. *Viruses* 2018:10. 10.3390/v10110643 30453598 PMC6266013

[B167] Lattanzi S. Leitinger M. Rocchi C. Salvemini S. Matricardi S. Brigo F. (2022). Unraveling the enigma of new-onset refractory status epilepticus: a systematic review of aetiologies. *Eur. J. Neurol.* 29 626–647. 10.1111/ene.15149 34661330 PMC9298123

[B168] Leal B. Chaves J. Carvalho C. Bettencourt A. Freitas J. Lopes J. (2017). Age of onset of mesial temporal lobe epilepsy with hippocampal sclerosis: the effect of apolipoprotein E and febrile seizures. *Int. J. Neurosci.* 127 800–804. 10.1080/00207454.2016.1264396 27875923

[B169] Lee A. J. Ashkar A. A. (2018). The Dual Nature of Type I and Type II Interferons. *Front. Immunol.* 9:2061. 10.3389/fimmu.2018.02061 30254639 PMC6141705

[B170] Lee H. Y. Chen C. J. Huang Y. C. Li W. C. Chiu C. H. Huang C. G. (2010). Clinical features of echovirus 6 and 9 infections in children. *J. Clin. Virol.* 49, 175–179. 10.1016/j.jcv.2010.07.010 20729140

[B171] Lee K. Y. Lee M. S. Kim D. B. (2016). Neurologic Manifestations of Enterovirus 71 Infection in Korea. *J. Korean Med. Sci.* 31 561–567. 10.3346/jkms.2016.31.4.561 27051240 PMC4810339

[B172] Lee K. Y. Lee Y. J. Kim T. H. Cheon D. S. Nam S. O. (2014). Clinico-radiological spectrum in enterovirus 71 infection involving the central nervous system in children. *J. Clin. Neurosci.* 21 416–420. 10.1016/j.jocn.2013.04.032 24169271

[B173] Lei H. Y. Yang D. Q. Li Y. X. Wang L. Q. Zheng M. (2015). Association between human cytomegalovirus and onset of epilepsy. *Int. J. Clin. Exp. Med.* 8 20556–20564. 26884973 PMC4723818

[B174] Leibovitch E. C. Jacobson S. (2015). Human Herpesvirus 6 as a Viral Trigger in Mesial Temporal Lobe Epilepsy. *J. Infect. Dis.* 212 1011–1013. 10.1093/infdis/jiv162 25840442

[B175] Lewis D. V. Shinnar S. Hesdorffer D. C. Bagiella E. Bello J. A. Chan S. (2014). Hippocampal sclerosis after febrile status epilepticus: the FEBSTAT study. *Ann. Neurol.* 75 178–185. 10.1002/ana.24081 24318290 PMC3980500

[B176] Lewthwaite P. Perera D. Ooi M. H. Last A. Kumar R. Desai A. (2010). Enterovirus 75 encephalitis in children, southern India. *Emerg. Infect. Dis.* 16 1780–1782. 10.3201/eid1611.100672 21029544 PMC3294525

[B177] Li C. C. Yang M. Y. Chen R. F. Lin T. Y. Tsao K. C. Ning H. C. (2002). Clinical manifestations and laboratory assessment in an enterovirus 71 outbreak in southern Taiwan. *Scand. J Infect. Dis.* 34 104–109. 10.1080/00365540110077119 11928838

[B178] Li J. M. Huang C. Yan B. Wang W. Zhou Q. Sander J. W. (2014). HHV-7 in adults with drug-resistant epilepsy: a pathological role in hippocampal sclerosis? *J. Clin. Virol.* 61 387–392. 10.1016/j.jcv.2014.08.017 25282530

[B179] Liao F. Yoon H. Kim J. (2017). Apolipoprotein E metabolism and functions in brain and its role in Alzheimer’s disease. *Curr. Opin. Lipidol.* 28 60–67. 10.1097/MOL.0000000000000383 27922847 PMC5213812

[B180] Libbey J. E. Fujinami R. S. (2011). Neurotropic viral infections leading to epilepsy: focus on Theiler’s murine encephalomyelitis virus. *Future Virol.* 6 1339–1350. 10.2217/fvl.11.107 22267964 PMC3259611

[B181] Libbey J. E. Fujinami R. S. (2014). Adaptive immune response to viral infections in the central nervous system. *Handb. Clin. Neurol.* 123 225–247. 10.1016/B978-0-444-53488-0.00010-9 25015488 PMC4370180

[B182] Libbey J. E. Fujinami R. S. (2021). Viral mouse models used to study multiple sclerosis: past and present. *Arch. Virol.* 166 1015–1033. 10.1007/s00705-021-04968-5 33582855 PMC7882042

[B183] Libbey J. E. Hanak T. J. Doty D. J. Wilcox K. S. Fujinami R. S. (2016). NBQX, a highly selective competitive antagonist of AMPA and KA ionotropic glutamate receptors, increases seizures and mortality following picornavirus infection. *Exp. Neurol.* 280 89–96. 10.1016/j.expneurol.2016.04.010 27072529 PMC4860063

[B184] Libbey J. E. Kennett N. J. Wilcox K. S. White H. S. Fujinami R. S. (2011). Lack of correlation of central nervous system inflammation and neuropathology with the development of seizures following acute virus infection. *J. Virol.* 85 8149–8157. 10.1128/JVI.00730-11 21680509 PMC3147986

[B185] Libbey J. E. Kirkman N. J. Smith M. C. Tanaka T. Wilcox K. S. White H. S. (2008). Seizures following picornavirus infection. *Epilepsia* 49 1066–1074. 10.1111/j.1528-1167.2008.01535.x 18325012

[B186] Lim Z. Q. Ng Q. Y. Oo Y. Chu J. J. H. Ng S. Y. Sze S. K. (2021). Enterovirus-A71 exploits peripherin and Rac1 to invade the central nervous system. *EMBO Rep.* 22:e51777. 10.15252/embr.202051777 33871166 PMC8183415

[B187] Loane D. J. Stoica B. A. Tchantchou F. Kumar A. Barrett J. P. Akintola T. (2014). Novel mGluR5 positive allosteric modulator improves functional recovery, attenuates neurodegeneration, and alters microglial polarization after experimental traumatic brain injury. *Neurotherapeutics* 11 857–869. 10.1007/s13311-014-0298-6 25096154 PMC4391388

[B188] Loewen J. L. Albertini G. Dahle E. J. Sato H. Smolders I. J. Massie A. (2019). Genetic and pharmacological manipulation of glial glutamate transporters does not alter infection-induced seizure activity. *Exp. Neurol.* 318 50–60. 10.1016/j.expneurol.2019.04.010 31022385

[B189] los Reyes E. C. McJunkin J. E. Glauser T. A. Tomsho M. O’Neal J. (2008). Periodic lateralized epileptiform discharges in La Crosse encephalitis, a worrisome subgroup: clinical presentation, electroencephalogram (EEG) patterns, and long-term neurologic outcome. *J. Child Neurol.* 23 167–172. 10.1177/0883073807307984 18160548

[B190] Löscher W. (2016). Fit for purpose application of currently existing animal models in the discovery of novel epilepsy therapies. *Epilepsy Res.* 126 157–184. 10.1016/j.eplepsyres.2016.05.016 27505294

[B191] Löscher W. (2021). Single-Target Versus Multi-Target Drugs Versus Combinations of Drugs With Multiple Targets: preclinical and Clinical Evidence for the Treatment or Prevention of Epilepsy. *Front. Pharmacol.* 12:730257. 10.3389/fphar.2021.730257 34776956 PMC8580162

[B192] Löscher W. Ebert U. (1996). Basic mechanisms of seizure propagation: Targets for rational drug design and rational polypharmacy. *Epilepsy Res. Suppl.* 11 17–44. 9294726

[B193] Löscher W. Friedman A. (2020). Structural, molecular and functional alterations of the blood-brain barrier during epileptogenesis and epilepsy: a cause, consequence or both? *Int. J Mol. Sci.* 21:591. 10.3390/ijms21020591 31963328 PMC7014122

[B194] Löscher W. Gernert M. Heinemann U. (2008). Cell and gene therapies in epilepsy–promising avenues or blind alleys? *Trends Neurosci.* 31 62–73. 10.1016/j.tins.2007.11.012 18201772

[B195] Löscher W. Hirsch L. J. Schmidt D. (2015). The enigma of the latent period in the development of symptomatic acquired epilepsy – Traditional view versus new concepts. *Epilepsy Behav.* 52 78–92. 10.1016/j.yebeh.2015.08.037 26409135

[B196] Löscher W. Klein P. (2021b). The pharmacology and clinical efficacy of antiseizure medications: From bromide salts to cenobamate and beyond. *CNS Drugs* 35 935–963. 10.1007/s40263-021-00827-8 34145528 PMC8408078

[B197] Löscher W. Klein P. (2021a). New approaches for developing multi-targeted drug combinations for disease modification of complex brain disorders. Does epilepsy prevention become a realistic goal? *Pharmacol. Ther.* 229:107934. 10.1016/j.pharmthera.2021.107934 34216705

[B198] Löscher W. Klitgaard H. Twyman R. E. Schmidt D. (2013). New avenues for antiepileptic drug discovery and development. *Nat. Rev. Drug Discov.* 12 757–776. 10.1038/nrd4126 24052047

[B199] Löscher W. Potschka H. (2005). Drug resistance in brain diseases and the role of drug efflux transporters. *Nat. Rev. Neurosci.* 6 591–602. 10.1038/nrn1728 16025095

[B200] Löscher W. Potschka H. Sisodiya S. M. Vezzani A. (2020). Drug Resistance in Epilepsy: Clinical Impact, Potential Mechanisms, and New Innovative Treatment Options. *Pharmacol. Rev.* 72 606–638. 10.1124/pr.120.019539 32540959 PMC7300324

[B201] Manangeeswaran M. Ireland D. D. Verthelyi D. (2016). Zika (PRVABC59) Infection Is Associated with T cell Infiltration and Neurodegeneration in CNS of Immunocompetent Neonatal C57Bl/6 Mice. *PLoS Pathog.* 12:e1006004. 10.1371/journal.ppat.1006004 27855206 PMC5113993

[B202] Manglani M. McGavern D. B. (2018). New advances in CNS immunity against viral infection. *Curr. Opin. Virol.* 28 116–126. 10.1016/j.coviro.2017.12.003 29289900 PMC5990251

[B203] Masuda T. Sankowski R. Staszewski O. Prinz M. (2020). Microglia Heterogeneity in the Single-Cell Era. *Cell Rep.* 30 1271–1281. 10.1016/j.celrep.2020.01.010 32023447

[B204] McClelland A. C. Gomes W. A. Shinnar S. Hesdorffer D. C. Bagiella E. Lewis D. V. (2016). Quantitative Evaluation of Medial Temporal Lobe Morphology in Children with Febrile Status Epilepticus: results of the FEBSTAT Study. *AJNR Am. J. Neuroradiol.* 37 2356–2362. 10.3174/ajnr.A4919 27633809 PMC5161621

[B205] McCoy M. K. Tansey M. G. (2008). TNF signaling inhibition in the CNS: implications for normal brain function and neurodegenerative disease. *J. Neuroinflam.* 5:45. 10.1186/1742-2094-5-45 18925972 PMC2577641

[B206] McEntire C. R. S. Song K. W. McInnis R. P. Rhee J. Y. Young M. Williams E. (2021). Neurologic Manifestations of the World Health Organization’s List of Pandemic and Epidemic Diseases. *Front. Neurol.* 12:634827. 10.3389/fneur.2021.634827 33692745 PMC7937722

[B207] McTague A. Martland T. Appleton R. (2018). Drug management for acute tonic-clonic convulsions including convulsive status epilepticus in children. *Cochrane Database Syst. Rev.* 1:CD001905. 10.1002/14651858.CD001905.pub3 PMC6491279 29320603

[B208] Meinhardt J. Radke J. Dittmayer C. Franz J. Thomas C. Mothes R. (2021). Olfactory transmucosal SARS-CoV-2 invasion as a port of central nervous system entry in individuals with COVID-19. *Nat. Neurosci.* 24 168–175. 10.1038/s41593-020-00758-5 33257876

[B209] Meldrum B. S. (1975). Present views on hippocampal sclerosis and epilepsy. *Mod. Trends Neurol.* 6 223–239. 1105141

[B210] Mendez-Fernandez Y. V. Johnson A. J. Rodriguez M. Pease L. R. (2003). Clearance of Theiler’s virus infection depends on the ability to generate a CD8+ T cell response against a single immunodominant viral peptide. *Eur. J. Immunol.* 33 2501–2510. 10.1002/eji.200324007 12938226

[B211] Metcalf C. S. Vanegas F. Underwood T. Johnson K. West P. J. Smith M. D. (2021). Screening of prototype antiseizure and anti-inflammatory compounds in the Theiler’s murine encephalomyelitis virus model of epilepsy. *Epilepsia Open* 7 46–58. 10.1002/epi4.12550 34668659 PMC8886069

[B212] Michael B. D. Solomon T. (2012). Seizures and encephalitis: clinical features, management, and potential pathophysiologic mechanisms. *Epilepsia* 53 (Suppl. 4), 63–71. 10.1111/j.1528-1167.2012.03615.x 22946723

[B213] Milano C. Turco F. Pizzanelli C. Pascazio A. Tagliaferri E. Nesti L. (2022). Ictogenesis of viral pneumonia: a comparison between SARS-CoV-2 and H1N1/H3N2. *Epilepsy Behav.* 126:108470. 10.1016/j.yebeh.2021.108470 34902662 PMC8661132

[B214] Millichap J. G. Millichap J. J. (2006). Role of viral infections in the etiology of febrile seizures. *Pediatr. Neurol.* 35 165–172. 10.1016/j.pediatrneurol.2006.06.004 16939854

[B215] Millichap J. G. (2015). Epstein-Barr virus neurologic complications. *Pediatr. Neurol. Briefs* 29:88. 10.15844/pedneurbriefs-29-11-7 26933545 PMC4747270

[B216] Milora K. A. Rall G. F. (2019). Interferon Control of Neurotropic Viral Infections. *Trends Immunol.* 40 842–856. 10.1016/j.it.2019.07.005 31439415

[B217] Mishra A. Kim H. J. Shin A. H. Thayer S. A. (2012). Synapse loss induced by interleukin-1Î^2^ requires pre- and post-synaptic mechanisms. *J. Neuroimmune. Pharmacol.* 7 571–578. 22311599 10.1007/s11481-012-9342-7 PMC3415563

[B218] Mishra R. Banerjea A. C. (2020). Neurological Damage by Coronaviruses: a Catastrophe in the Queue. *Front. Immunol.* 11:565521. 10.3389/fimmu.2020.565521 33013930 PMC7511585

[B219] Misra U. K. Tan C. T. Kalita J. (2008). Viral encephalitis and epilepsy. *Epilepsia* 49 (Suppl. 6), 13–18. 10.1111/j.1528-1167.2008.01751.x 18754956

[B220] Mistchenko A. S. Viegas M. Latta M. P. Barrero P. R. (2006). Molecular and epidemiologic analysis of enterovirus B neurological infection in Argentine children. *J. Clin. Virol.* 37 293–299. 10.1016/j.jcv.2006.08.009 16982209

[B221] Modlin J. F. Dagan R. Berlin L. E. Virshup D. M. Yolken R. H. Menegus M. (1991). Focal encephalitis with enterovirus infections. *Pediatrics* 88 841–845. 10.1542/peds.88.4.841 1896296

[B222] Mohan N. Fayyaz M. A. Del Rio C. Khurana N. K. R. S. Vaidya S. S. Salazar E. (2021). Neurological manifestations and neuroimaging findings in patients with SARS-CoV2-a systematic review. *Egypt. J. Neurol. Psychiatr. Neurosurg.* 57 68. 10.1186/s41983-021-00322-3 34093004 PMC8170868

[B223] Montagne A. Nation D. A. Sagare A. P. Barisano G. Sweeney M. D. Chakhoyan A. (2020). APOE4 leads to blood-brain barrier dysfunction predicting cognitive decline. *Nature* 581 71–76. 10.1038/s41586-020-2247-3 32376954 PMC7250000

[B224] Montalvan V. Lee J. Bueso T. De Toledo J. Rivas K. (2020). Neurological manifestations of COVID-19 and other coronavirus infections: a systematic review. *Clin. Neurol. Neurosurg.* 194:105921. 10.1016/j.clineuro.2020.105921 32422545 PMC7227498

[B225] Morrison T. E. Diamond M. S. (2017). Animal Models of Zika Virus Infection, Pathogenesis, and Immunity. *J. Virol.* 2017:91. 10.1128/JVI.00009-17 28148798 PMC5375682

[B226] Munoz-Fontela C. Dowling W. E. Funnell S. G. P. Gsell P. S. Riveros-Balta A. X. Albrecht R. A. (2020). Animal models for COVID-19. *Nature* 586 509–515. 32967005 10.1038/s41586-020-2787-6 PMC8136862

[B227] Murao A. Aziz M. Wang H. Brenner M. Wang P. (2021). Release mechanisms of major DAMPs. *Apoptosis* 26 152–162. 10.1007/s10495-021-01663-3 33713214 PMC8016797

[B228] Murta V. Villarreal A. Ramos A. J. (2020). Severe Acute Respiratory Syndrome Coronavirus 2 Impact on the Central Nervous System: are Astrocytes and Microglia Main Players or Merely Bystanders? *ASN. Neuro.* 12:1759091420954960. 10.1177/1759091420954960 32878468 PMC7476346

[B229] Nalbandian A. Sehgal K. Gupta A. Madhavan M. V. McGroder C. Stevens J. S. (2021). Post-acute COVID-19 syndrome. *Nat. Med.* 27 601–615. 33753937 10.1038/s41591-021-01283-z PMC8893149

[B230] Nassir C. M. N. Hashim S. Wong K. K. Abdul H. S. Idris N. S. Jayabalan N. (2021). COVID-19 Infection and Circulating Microparticles-Reviewing Evidence as Microthrombogenic Risk Factor for Cerebral Small Vessel Disease. *Mol. Neurobiol.* 58 4188–4215. 10.1007/s12035-021-02457-z 34176095 PMC8235918

[B231] Nath A. Johnson T. P. (2021). Mechanisms of viral persistence in the brain and therapeutic approaches. *FEBS J.* 2021:15871. 10.1111/febs.15871 33844441

[B232] Nausch E. Schaffeldt L. Tautorat I. Margraf N. G. HÃusler M. Kluger G. (2022). New-onset refractory status epilepticus (NORSE) and febrile infection-related epilepsy syndrome (FIRES) of unknown aetiology: a comparison of the incomparable? *Seizure* 96 18–21. 10.1016/j.seizure.2022.01.006 35042004

[B233] Nem de Oliveira Souza I. Frost P. S. Franca J. V. Nascimento-Viana J. B. Neris R. L. S. Freitas L. (2018). Acute and chronic neurological consequences of early-life Zika virus infection in mice. *Sci. Transl. Med.* 2018:10. 10.1126/scitranslmed.aar2749 29875203

[B234] Nikbakht F. Mohammadkhanizadeh A. Mohammadi E. (2020). How does the COVID-19 cause seizure and epilepsy in patients? The potential mechanisms. *Mult. Scler. Relat. Disord.* 46:102535. 10.1016/j.msard.2020.102535 33010584 PMC7521932

[B235] Noé F. M. Polascheck N. Frigerio F. Bankstahl M. Ravizza T. Marchini S. (2013). Pharmacological blockade of IL-1beta/IL-1 receptor type 1 axis during epileptogenesis provides neuroprotection in two rat models of temporal lobe epilepsy. *Neurobiol. Dis.* 59 183–193. 10.1016/j.nbd.2013.07.015 23938763

[B236] Nolen L. T. Mukerji S. S. Mejia N. I. (2022). Post-acute neurological consequences of COVID-19: an unequal burden. *Nat. Med.* 28 20–23. 10.1038/s41591-021-01647-5 35039657

[B237] Ojha D. Winkler C. W. Leung J. M. Woods T. A. Chen C. Z. Nair V. (2021). Rottlerin inhibits La Crosse virus-induced encephalitis in mice and blocks release of replicating virus from the Golgi body in neurons. *Nat. Microbiol.* 6 1398–1409. 10.1038/s41564-021-00968-y 34675384

[B238] Ong K. C. Wong K. T. (2015). Understanding Enterovirus 71 Neuropathogenesis and Its Impact on Other Neurotropic Enteroviruses. *Brain Pathol.* 25 614–624. 10.1111/bpa.12279 26276025 PMC8029433

[B239] Ooi M. H. Wong S. C. Lewthwaite P. Cardosa M. J. Solomon T. (2010). Clinical features, diagnosis, and management of enterovirus 71. *Lancet Neurol.* 9 1097–1105. 10.1016/S1474-4422(10)70209-X 20965438

[B240] O’Shea J. J. Gadina M. Siegel R. (2013). “Cytokines and cytokine receptors,” in *Clinical Immunology E-Book: Principles and Practice. Fourth edition*, eds Rich R. R. Fleisher T. A. Shearer W. T. Jr H.W. Schroeder Frew A. J. Weyand C. M. (Amsterdam: Elsevier Saunders), 108–135.

[B241] Outram G. W. Dickinson A. G. Fraser H. (1975). Slow encephalopathies, inflammatory responses and arachis oil. *Lancet* 1 198–200. 10.1016/s0140-6736(75)91363-x 47424

[B242] Pacheco-Herrero M. Soto-Rojas L. O. Harrington C. R. Flores-Martinez Y. M. Villegas-Rojas M. M. Leon-Aguilar A. M. (2021). Elucidating the Neuropathologic Mechanisms of SARS-CoV-2 Infection. *Front. Neurol.* 12:660087. 10.3389/fneur.2021.660087 33912129 PMC8072392

[B243] Palus M. Bily T. Elsterova J. Langhansova H. Salat J. Vancova M. (2014). Infection and injury of human astrocytes by tick-borne encephalitis virus. *J. Gen. Virol.* 95 2411–2426. 10.1099/vir.0.068411-0 25000960

[B244] Pape K. Tamouza R. Leboyer M. Zipp F. (2019). Immunoneuropsychiatry - novel perspectives on brain disorders. *Nat. Rev. Neurol.* 15 317–328. 10.1038/s41582-019-0174-4 30988501

[B245] Pascual-Goni E. Josa M. Launes C. Querol L. Del Cuerpo M. Bosch M. A. (2019). Excellent Response to Plasma Exchange in Three Patients With Enterovirus-71 Neurological Disease. *Front. Neurol.* 10:548. 10.3389/fneur.2019.00548 31178823 PMC6542981

[B246] Patel D. C. Wallis G. Dahle E. J. McElroy P. B. Thomson K. E. Tesi R. J. (2017). Hippocampal TNFÎ± Signaling Contributes to Seizure Generation in an Infection-Induced Mouse Model of Limbic Epilepsy. *eNeuro* 2017:4. 10.1523/ENEURO.0105-17.2017 28497109 PMC5422919

[B247] Patel D. C. Wallis G. Fujinami R. S. Wilcox K. S. Smith M. D. (2019). Cannabidiol reduces seizures following CNS infection with Theiler’s murine encephalomyelitis virus. *Epilepsia Open* 4 431–442. 10.1002/epi4.12351 31440724 PMC6698680

[B248] Patick A. K. Pease L. R. David C. S. Rodriguez M. (1990). Major histocompatibility complex-conferred resistance to Theiler’s virus-induced demyelinating disease is inherited as a dominant trait in B10 congenic mice. *J. Virol.* 64, 5570–5576. 10.1128/jvi.64.11.5570-5576.1990 2214025 PMC248609

[B249] Pellegrini L. Albecka A. Mallery D. L. Kellner M. J. Paul D. Carter A. P. (2020). SARS-CoV-2 Infects the Brain Choroid Plexus and Disrupts the Blood-CSF Barrier in Human Brain Organoids. *Cell Stem Cell* 27 951–961. 10.1016/j.stem.2020.10.001 33113348 PMC7553118

[B250] Peterson A. R. Binder D. K. (2020). Astrocyte Glutamate Uptake and Signaling as Novel Targets for Antiepileptogenic Therapy. *Front. Neurol.* 11:1006. 10.3389/fneur.2020.01006 33013665 PMC7505989

[B251] Petrovszki D. Walter F. R. Vigh J. P. Kocsis A. Valkai S. Deli M. A. (2022). Penetration of the SARS-CoV-2 Spike Protein across the Blood-Brain Barrier, as Revealed by a Combination of a Human Cell Culture Model System and Optical Biosensing. *Biomedicines* 2022:10. 10.3390/biomedicines10010188 35052867 PMC8773803

[B252] Pillai S. C. Mohammad S. S. Hacohen Y. Tantsis E. Prelog K. Barnes E. H. (2016). Postencephalitic epilepsy and drug-resistant epilepsy after infectious and antibody-associated encephalitis in childhood: Clinical and etiologic risk factors. *Epilepsia* 57 e7–e11. 10.1111/epi.13253 26592968

[B253] Pinheiro D. J. L. L. Oliveira L. F. Souza I. N. O. Brogin J. A. F. Bueno D. D. Miranda I. A. (2020). Modulation in phase and frequency of neural oscillations during epileptiform activity induced by neonatal Zika virus infection in mice. *Sci. Rep.* 10:6763. 10.1038/s41598-020-63685-2 32317689 PMC7174408

[B254] Pitkänen A. Lukasiuk K. (2011). Mechanisms of epileptogenesis and potential treatment targets. *Lancet Neurol.* 10 173–186. 10.1016/S1474-4422(10)70310-0 21256455

[B255] Pitsch J. Kuehn J. C. Gnatkovsky V. Muller J. A. van Loo K. M. J. De Curtis M. (2019). Anti-epileptogenic and Anti-convulsive Effects of Fingolimod in Experimental Temporal Lobe Epilepsy. *Mol. Neurobiol.* 56 1825–1840. 10.1007/s12035-018-1181-y 29934763

[B256] Popkirov S. Ismail F. S. Granheit W. Kapauer M. Wellmer J. Bien C. G. (2017). Progressive hippocampal sclerosis after viral encephalitis: potential role of NMDA receptor antibodies. *Seizure* 51 6–8. 10.1016/j.seizure.2017.07.006 28750305

[B257] Potokar M. JorgaAevski J. Zorec R. (2019). Astrocytes in Flavivirus Infections. *Int. J. Mol. Sci.* 2019:20. 10.3390/ijms20030691 30736273 PMC6386967

[B258] Potter M. C. Figuera-Losada M. Rojas C. Slusher B. S. (2013). Targeting the glutamatergic system for the treatment of HIV-associated neurocognitive disorders. *J. Neuroimmune. Pharmacol.* 8 594–607. 10.1007/s11481-013-9442-z 23553365 PMC3661915

[B259] Powell A. M. Black M. M. (2001). Epitope spreading: protection from pathogens, but propagation of autoimmunity? *Clin. Exp. Dermatol.* 26 427–433. 10.1046/j.1365-2230.2001.00852.x 11488833

[B260] Prinz M. Priller J. (2010). Tickets to the brain: role of CCR2 and CX3CR1 in myeloid cell entry in the CNS. *J. Neuroimmunol.* 224 80–84. 10.1016/j.jneuroim.2010.05.015 20554025

[B261] Prinz M. Priller J. (2017). The role of peripheral immune cells in the CNS in steady state and disease. *Nat. Neurosci.* 20 136–144. 10.1038/nn.4475 28092660

[B262] Prinz M. Priller J. Sisodia S. S. Ransohoff R. M. (2011). Heterogeneity of CNS myeloid cells and their roles in neurodegeneration. *Nat. Neurosci.* 14 1227–1235. 10.1038/nn.2923 21952260

[B263] Pröbstel A. K. Schirmer L. (2021). SARS-CoV-2-specific neuropathology: fact or fiction? *Trends Neurosci.* 44 933–935. 10.1016/j.tins.2021.10.006 34716032 PMC8519811

[B264] Proper E. A. Hoogland G. Kappen S. M. Jansen G. H. Rensen M. G. Schrama L. H. (2002). Distribution of glutamate transporters in the hippocampus of patients with pharmaco-resistant temporal lobe epilepsy. *Brain* 125 32–43. 10.1093/brain/awf001 11834591

[B265] Puenpa J. Wanlapakorn N. Vongpunsawad S. Poovorawan Y. (2019). The History of Enterovirus A71 Outbreaks and Molecular Epidemiology in the Asia-Pacific Region. *J. Biomed. Sci.* 26:75. 10.1186/s12929-019-0573-2 31627753 PMC6798416

[B266] Racine R. J. (1972). Modification of seizure activity by electrical stimulation: II. Motor seizure. *Electroenceph. Clin. Neurophysiol.* 32 281–294. 10.1016/0013-4694(72)90177-0 4110397

[B267] Raper J. Kovacs-Balint Z. Mavigner M. Gumber S. Burke M. W. Habib J. (2020). Long-term alterations in brain and behavior after postnatal Zika virus infection in infant macaques. *Nat. Commun.* 11:2534. 10.1038/s41467-020-16320-7 32439858 PMC7242369

[B268] Ravizza T. Terrone G. Salamone A. Frigerio F. Balosso S. Antoine D. J. (2018). High Mobility Group Box 1 is a novel pathogenic factor and a mechanistic biomarker for epilepsy. *Brain Behav. Immun.* 72 14–21. 10.1016/j.bbi.2017.10.008 29031614

[B269] Reid A. Y. Riazi K. Campbell T. G. Pittman Q. J. (2013). Increased excitability and molecular changes in adult rats after a febrile seizure. *Epilepsia* 54 e45–e48. 10.1111/epi.12061 23293960

[B270] Remsik J. Wilcox J. A. Babady N. E. McMillen T. A. Vachha B. A. Halpern N. A. (2021). Inflammatory Leptomeningeal Cytokines Mediate COVID-19 Neurologic Symptoms in Cancer Patients. *Cancer Cell* 39 276–283. 10.1016/j.ccell.2021.01.007 33508216 PMC7833316

[B271] Ren R. B. Costantini F. Gorgacz E. J. Lee J. J. Racaniello V. R. (1990). Transgenic mice expressing a human poliovirus receptor: a new model for poliomyelitis. *Cell* 63 353–362. 10.1016/0092-8674(90)90168-e 2170026

[B272] Reynaud J. M. Horvat B. (2013). Animal models for human herpesvirus 6 infection. *Front. Microbiol.* 4:174. 10.3389/fmicb.2013.00174 23847599 PMC3701164

[B273] Rhea E. M. Logsdon A. F. Hansen K. M. Williams L. M. Reed M. J. Baumann K. K. (2021). The S1 protein of SARS-CoV-2 crosses the blood-brain barrier in mice. *Nat. Neurosci.* 24 368–378. 10.1038/s41593-020-00771-8 33328624 PMC8793077

[B274] Riazi K. Galic M. A. Kentner A. C. Reid A. Y. Sharkey K. A. Pittman Q. J. (2015). Microglia-dependent alteration of glutamatergic synaptic transmission and plasticity in the hippocampus during peripheral inflammation. *J. Neurosci.* 35 4942–4952. 10.1523/JNEUROSCI.4485-14.2015 25810524 PMC6705378

[B275] Riazi K. Galic M. A. Pittman Q. J. (2010). Contributions of peripheral inflammation to seizure susceptibility: cytokines and brain excitability. *Epilepsy Res.* 89 34–42. 10.1016/j.eplepsyres.2009.09.004 19804959

[B276] Ribak C. E. Gall C. M. Mody I. (1992). *The dentate gyrus and its role in seizures.* Amsterdam: Elsevier.

[B277] Rock R. B. Gekker G. Hu S. Sheng W. S. Cheeran M. Lokensgard J. R. (2004). Role of microglia in central nervous system infections. *Clin. Microbiol. Rev.* 17 942–964. 15489356 10.1128/CMR.17.4.942-964.2004 PMC523558

[B278] Rodriguez M. Zoecklein L. J. Howe C. L. Pavelko K. D. Gamez J. D. Nakane S. (2003). Gamma interferon is critical for neuronal viral clearance and protection in a susceptible mouse strain following early intracranial Theiler’s murine encephalomyelitis virus infection. *J. Virol.* 77 12252–12265. 10.1128/jvi.77.22.12252-12265.2003 14581562 PMC254254

[B279] Rotbart H. A. (1995). Enteroviral infections of the central nervous system. *Clin. Infect. Dis.* 20 971–981. 10.1093/clinids/20.4.971 7795102

[B280] Rubboli G. Franceschetti S. Berkovic S. F. Canafoglia L. Gambardella A. Dibbens L. M. (2011). Clinical and neurophysiologic features of progressive myoclonus epilepsy without renal failure caused by SCARB2 mutations. *Epilepsia* 52 2356–2363. 10.1111/j.1528-1167.2011.03307.x 22050460

[B281] Rudolph H. Gress K. Weiss C. Schroten H. Adams O. Tenenbaum T. (2021). General Characteristics of Children with Single- and Co-Infections and Febrile Seizures with a Main Focus on Respiratory Pathogens: Preliminary Results. *Pathogens* 2021:10. 10.3390/pathogens10081061 34451525 PMC8399297

[B282] Ruzek D. Salat J. Singh S. K. Kopecky J. (2011). Breakdown of the blood-brain barrier during tick-borne encephalitis in mice is not dependent on CD^8 +^ T-cells. *PLoS One* 6:e20472. 10.1371/journal.pone.0020472 21629771 PMC3100324

[B283] Salimi H. Cain M. D. Jiang X. Roth R. A. Beatty W. L. Sun C. (2020). Encephalitic Alphaviruses Exploit Caveola-Mediated Transcytosis at the Blood-Brain Barrier for Central Nervous System Entry. *mBio* 2020:11. 10.1128/mBio.02731-19 32047126 PMC7018649

[B284] Sanchez J. M. S. DePaula-Silva A. B. Doty D. J. Truong A. Libbey J. E. Fujinami R. S. (2019). Microglial cell depletion is fatal with low levelÂ picornavirus infection of the central nervous system. *J. Neurovirol.* 25 415–421. 10.1007/s13365-019-00740-3 30859497 PMC6635090

[B285] Sandler C. X. Wyller V. B. B. Moss-Morris R. Buchwald D. Crawley E. Hautvast J. (2021). Long COVID and Post-infective Fatigue Syndrome: a Review. *Open Forum Infect. Dis.* 8:ofab440. 10.1093/ofid/ofab440 34631916 PMC8496765

[B286] Sankowski R. Monaco G. Prinz M. (2021). Evaluating microglial phenotypes using single-cell technologies. *Trends Neurosci.* 45:1. 10.1016/j.tins.2021.11.001 34872773

[B287] Sberna G. Capobianchi M. R. Bordi L. Lalle E. (2020). Letter of concern re: “Analysis of Covid-19 and non-Covid-19 viruses, including influenza viruses, to determine the influence of intensive preventive measures in Japan. *J. Clin. Virol.* 132:104635. 10.1016/j.jcv.2020.104635 32942138 PMC7480735

[B288] Scharfman H. E. (2019). The Dentate Gyrus and Temporal Lobe Epilepsy: an “Exciting”. *Era. Epilepsy Curr.* 19 249–255. 10.1177/1535759719855952 31232111 PMC6891841

[B289] Schetters S. T. T. Gomez-Nicola D. Garcia-Vallejo J. J. Van Kooyk Y. (2017). Neuroinflammation: Microglia and T Cells Get Ready to Tango. *Front. Immunol.* 8:1905. 10.3389/fimmu.2017.01905 29422891 PMC5788906

[B290] Schidlitzki A. Bascunana P. Srivastava P. K. Welzel L. Twele F. Töllner K. (2020). Proof-of-concept that network pharmacology is effective to modify development of acquired temporal lobe epilepsy. *Neurobiol. Dis.* 134:104664. 10.1016/j.nbd.2019.104664 31678583

[B291] Schmidt N. J. Lennette E. H. Ho H. H. (1974). An apparently new enterovirus isolated from patients with disease of the central nervous system. *J. Infect. Dis.* 129 304–309. 10.1093/infdis/129.3.304 4361245

[B292] Sculier C. Barcia A. C. Gaspard N. Gaanza-Lein M. Sanchez F. N. I Amengual-Gual M. (2021). Clinical presentation of new onset refractory status epilepticus in children (the pSERG cohort). *Epilepsia* 62 1629–1642. 10.1111/epi.16950 34091885 PMC8362203

[B293] Sehl J. Hölper J. E. Klupp B. G. Baumbach C. Teifke J. P. Mettenleiter T. C. (2020). An improved animal model for herpesvirus encephalitis in humans. *PLoS Pathog.* 16:e1008445. 10.1371/journal.ppat.1008445 32226043 PMC7145201

[B294] Sellner J. Trinka E. (2012). Seizures and epilepsy in herpes simplex virus encephalitis: current concepts and future directions of pathogenesis and management. *J. Neurol.* 259 2019–2030. 10.1007/s00415-012-6494-6 22527234

[B295] Shehata G. A. Lord K. C. Grudzinski M. C. Elsayed M. Abdelnaby R. Elshabrawy H. A. (2021). Neurological Complications of COVID-19: Underlying Mechanisms and Management. *Int. J. Mol. Sci.* 2021:22. 10.3390/ijms22084081 33920904 PMC8071289

[B296] Shinnar S. (2003). Febrile Seizures and Mesial Temporal Sclerosis. *Epilepsy Curr.* 3 115–118. 10.1046/j.1535-7597.2003.03401.x 15309049 PMC321192

[B297] Shinnar S. Bello J. A. Chan S. Hesdorffer D. C. Lewis D. V. MacFall J. (2012). MRI abnormalities following febrile status epilepticus in children: the FEBSTAT study. *Neurology* 79 871–877. 10.1212/WNL.0b013e318266fcc5 22843278 PMC3425848

[B298] Singh R. K. Dhama K. Chakraborty S. Tiwari R. Natesan S. Khandia R. (2019). Nipah virus: epidemiology, pathology, immunobiology and advances in diagnosis, vaccine designing and control strategies - a comprehensive review. *Vet. Q.* 39 26–55. 10.1080/01652176.2019.1580827 31006350 PMC6830995

[B299] Singhi P. (2011). Infectious causes of seizures and epilepsy in the developing world. *Dev. Med. Child Neurol.* 53 600–609. 10.1111/j.1469-8749.2011.03928.x 21518343

[B300] Smeal R. M. Fujinami R. White H. S. Wilcox K. S. (2015). Decrease in CA3 inhibitory network activity during Theiler’s virus encephalitis. *Neurosci. Lett.* 609 210–215. 10.1016/j.neulet.2015.10.032 26477780 PMC4867493

[B301] Smeal R. M. Stewart K. A. Iacob E. Fujinami R. S. White H. S. Wilcox K. S. (2012). The activity within the CA3 excitatory network during Theiler’s virus encephalitis is distinct from that observed during chronic epilepsy. *J. Neurovirol.* 18 30–44. 10.1007/s13365-012-0082-5 22328242 PMC4397904

[B302] Sofroniew M. V. Vinters H. V. (2010). Astrocytes: biology and pathology. *Acta Neuropathol.* 119 7–35. 10.1007/s00401-009-0619-8 20012068 PMC2799634

[B303] Soma N. Aizawa Y. Matsunaga M. Saitoh A. (2021). Clinically Mild Encephalitis/Encephalopathy with a Reversible Splenial Lesion Associated with Rhinovirus. *Pediatr. Infect. Dis. J* 40 e122–e125. 10.1097/INF.0000000000002995 33464018

[B304] Sommer W. (1880). Erkrankung des Ammonshorns als aetiologisches Moment der Epilepsie. *Arch. Psychiatrie Nervenkrankh.* 10 631–675. 10.1007/bf02224538

[B305] Song E. Zhang C. Israelow B. Lu-Culligan A. Prado A. V. Skriabine S. (2021). Neuroinvasion of SARS-CoV-2 in human and mouse brain. *J. Exp. Med.* 2021:218. 10.1084/jem.20202135 PMC7808299 33433624

[B306] Speck S. H. Ganem D. (2010). Viral latency and its regulation: lessons from the gamma-herpesviruses. *Cell Host. Microbe* 8 100–115. 10.1016/j.chom.2010.06.014 20638646 PMC2914632

[B307] Spiteri A. G. Wishart C. L. Pamphlett R. Locatelli G. King N. J. C. (2022). Microglia and monocytes in inflammatory CNS disease: integrating phenotype and function. *Acta Neuropathol.* 143 179–224. 10.1007/s00401-021-02384-2 34853891 PMC8742818

[B308] Stanelle-Bertram S. Walendy-Gnirss K. Speiseder T. Thiele S. Asante I. A. Dreier C. (2018). Male offspring born to mildly ZIKV-infected mice are at risk of developing neurocognitive disorders in adulthood. *Nat. Microbiol.* 3 1161–1174. 10.1038/s41564-018-0236-1 30202017

[B309] Stewart K. A. Wilcox K. S. Fujinami R. S. White H. S. (2010a). Development of postinfection epilepsy after Theiler’s virus infection of C57BL/6 mice. *J. Neuropathol. Exp. Neurol.* 69 1210–1219. 10.1097/NEN.0b013e3181ffc420 21107134 PMC3077028

[B310] Stewart K. A. Wilcox K. S. Fujinami R. S. White H. S. (2010b). Theiler’s virus infection chronically alters seizure susceptibility. *Epilepsia* 51 1418–1428. 10.1111/j.1528-1167.2009.02405.x 20002148

[B311] Stredny C. M. Case S. Sansevere A. J. Son M. Henderson L. Gorman M. P. (2020). Interleukin-6 Blockade With Tocilizumab in Anakinra-Refractory Febrile Infection-Related Epilepsy Syndrome (FIRES). *Child Neurol. Open* 7:2329048X20979253. 10.1177/2329048X20979253 33403221 PMC7745547

[B312] Suen W. W. Prow N. A. Hall R. A. Bielefeldt-Ohmann H. (2014). Mechanism of West Nile virus neuroinvasion: a critical appraisal. *Viruses* 6 2796–2825. 10.3390/v6072796 25046180 PMC4113794

[B313] Sulzer D. Antonini A. Leta V. Nordvig A. Smeyne R. J. Goldman J. E. (2020). COVID-19 and possible links with Parkinson’s disease and parkinsonism: from bench to bedside. *NPJ. Parkinsons. Dis.* 6:18. 10.1038/s41531-020-00123-0 32885037 PMC7441399

[B314] Surana P. Tang S. McDougall M. Tong C. Y. Menson E. Lim M. (2011). Neurological complications of pandemic influenza A H1N1 2009 infection: european case series and review. *Eur. J. Pediatr.* 170 1007–1015. 10.1007/s00431-010-1392-3 21234600 PMC7086688

[B315] Sutula T. P. Dudek F. E. (2007). Unmasking recurrent excitation generated by mossy fiber sprouting in the epileptic dentate gyrus: an emergent property of a complex system. *Prog. Brain Res.* 163 541–563. 10.1016/S0079-6123(07)63029-5 17765737

[B316] Suzuki Y. Toribe Y. Mogami Y. Yanagihara K. Nishikawa M. (2008). Epilepsy in patients with congenital cytomegalovirus infection. *Brain Dev.* 30 420–424. 10.1016/j.braindev.2007.12.004 18215482

[B317] Swissa E. Serlin Y. Vazana U. Prager O. Friedman A. (2019). Blood-brain barrier dysfunction in status epileptics: Mechanisms and role in epileptogenesis. *Epilepsy Behav.* 101:106285. 10.1016/j.yebeh.2019.04.038 31711869

[B318] Tan T. H. Perucca P. O’Brien T. J. Kwan P. Monif M. (2021). Inflammation, ictogenesis, and epileptogenesis: An exploration through human disease. *Epilepsia* 62 303–324. 10.1111/epi.16788 33316111

[B319] Tavkar P. Potokar M. Kolenc M. Korva M. Avsic-Zupanc T. Zorec R. (2021). Neurotropic Viruses. Astrocytes, and COVID-19. *Front. Cell Neurosci.* 15:662578. 10.3389/fncel.2021.662578 33897376 PMC8062881

[B320] Teleron A. L. Rose B. K. Williams D. M. Kemper S. E. McJunkin J. E. (2016). La Crosse Encephalitis: an Adult Case Series. *Am. J. Med.* 129 881–884. 10.1016/j.amjmed.2016.03.021 27086496

[B321] Temkin N. R. Dikmen S. S. Wilensky A. J. Keihm J. Chabal S. Winn H. R. (1990). A randomized, double-blind study of phenytoin for the prevention of post-traumatic seizures. *N. Engl. J. Med.* 323 497–502. 10.1056/NEJM199008233230801 2115976

[B322] Teoh H. L. Mohammad S. S. Britton P. N. Kandula T. Lorentzos M. S. Booy R. (2016). Clinical Characteristics and Functional Motor Outcomes of Enterovirus 71 Neurological Disease in Children. *JAMA Neurol.* 73 300–307. 10.1001/jamaneurol.2015.4388 26785318

[B323] Terry R. L. Getts D. R. Deffrasnes C. van Vreden C. Campbell I. L. King N. J. (2012). Inflammatory monocytes and the pathogenesis of viral encephalitis. *J. Neuroinflammation* 9:270. 10.1186/1742-2094-9-270 23244217 PMC3560265

[B324] Theiler M. (1934). Spontaneous encephalomyelitis of mice–a new virus disease. *Science* 80:122. 10.1126/science.80.2066.122-b 17750712

[B325] Theiler M. (1937). Spontaneous encephalomyelitis of mice, a new virus disease. *J. Exp. Med.* 65 705–719. 10.1084/jem.65.5.705 19870629 PMC2133518

[B326] Theiler M. Gard S. (1940a). Encephalomyelitis of mice : i. characteristics and pathogenesis of the virus. *J. Exp. Med.* 72 49–67. 10.1084/jem.72.1.49 19871007 PMC2135012

[B327] Theiler M. Gard S. (1940b). Encephalomyelitis of mice : iii. epidemiology. *J. Exp. Med.* 72 79–90. 10.1084/jem.72.1.79 19871009 PMC2135011

[B328] Theodore W. H. (2014). Epilepsy and viral infections. *Epilepsy Curr.* 14 35–42. 10.5698/1535-7511-14.s2.35 24955074 PMC3966643

[B329] Thom M. (2014). Review: Hippocampal sclerosis in epilepsy: a neuropathology review. *Neuropathol. Appl. Neurobiol.* 40 520–543. 10.1111/nan.12150 24762203 PMC4265206

[B330] Thong W. Y. Han A. Wang S. J. Lin J. Isa M. S. Koay E. S. (2017). Enterovirus infections in Singaporean children: an assessment of neurological manifestations and clinical outcomes. *Singapore Med. J* 58 189–195. 10.11622/smedj.2016099 27245861 PMC5392603

[B331] Tomasoni R. Morini R. Lopez-Atalaya J. P. Corradini I. Canzi A. Rasile M. (2017). Lack of IL-1R8 in neurons causes hyperactivation of IL-1 receptor pathway and induces MECP2-dependent synaptic defects. *Elife* 2017:6. 10.7554/eLife.21735 28347403 PMC5370184

[B332] Too I. H. Yeo H. Sessions O. M. Yan B. Libau E. A. Howe J. L. (2016). Enterovirus 71 infection of motor neuron-like NSC-34 cells undergoes a non-lytic exit pathway. *Sci. Rep.* 6:36983. 10.1038/srep36983 27849036 PMC5111112

[B333] Uhde A. K. Ciurkiewicz M. Herder V. Khan M. A. Hensel N. Claus P. (2018). Intact interleukin-10 receptor signaling protects from hippocampal damage elicited by experimental neurotropic virus infection of SJL mice. *Sci. Rep.* 8:6106. 10.1038/s41598-018-24378-z 29666403 PMC5904160

[B334] Umpierre A. D. Remigio G. J. Dahle E. J. Bradford K. Alex A. B. Smith M. D. (2014). Impaired cognitive ability and anxiety-like behavior following acute seizures in the Theiler’s virus model of temporal lobe epilepsy. *Neurobiol. Dis.* 64 98–106. 10.1016/j.nbd.2013.12.015 24412221 PMC4353639

[B335] van Marle G. Antony J. Ostermann H. Dunham C. Hunt T. Halliday W. (2007). West Nile virus-induced neuroinflammation: glial infection and capsid protein-mediated neurovirulence. *J. Virol.* 81 10933–10949. 10.1128/JVI.02422-06 17670819 PMC2045515

[B336] Vargas G. Medeiros Geraldo L. H. Gedeao S. N. Viana P. M. Regina Souza L. F. Carvalho Alcantara G. F. (2020). Severe acute respiratory syndrome coronavirus 2 (SARS-CoV-2) and glial cells: Insights and perspectives. *Brain Behav. Immun. Health* 7:100127. 10.1016/j.bbih.2020.100127 32838339 PMC7423575

[B337] Varvel N. H. Neher J. J. Bosch A. Wang W. Ransohoff R. M. Miller R. J. (2016). Infiltrating monocytes promote brain inflammation and exacerbate neuronal damage after status epilepticus. *Proc. Natl. Acad. Sci. U. S. A* 113 E5665–E5674. 10.1073/pnas.1604263113 27601660 PMC5035862

[B338] Venkatesan A. Benavides D. R. (2015). Autoimmune encephalitis and its relation to infection. *Curr. Neurol. Neurosci. Rep.* 15:3. 10.1007/s11910-015-0529-1 25637289

[B339] Verboon-Maciolek M. A. Krediet T. G. Gerards L. J. De Vries L. S. Groenendaal F. van Loon A. M. (2008). Severe neonatal parechovirus infection and similarity with enterovirus infection. *Pediatr. Infect. Dis. J* 27 241–245. 10.1097/INF.0b013e31815c1b07 18277927

[B340] Verhoog Q. P. Holtman L. Aronica E. van Vliet E. A. (2020). Astrocytes as Guardians of Neuronal Excitability: mechanisms Underlying Epileptogenesis. *Front. Neurol.* 11:591690. 10.3389/fneur.2020.591690 33324329 PMC7726323

[B341] Verma S. Lo Y. Chapagain M. Lum S. Kumar M. Gurjav U. (2009). West Nile virus infection modulates human brain microvascular endothelial cells tight junction proteins and cell adhesion molecules: Transmigration across the in vitro blood-brain barrier. *Virology* 385 425–433. 10.1016/j.virol.2008.11.047 19135695 PMC2684466

[B342] Vezzani A. (2014). Epilepsy and inflammation in the brain: overview and pathophysiology. *Epilepsy Curr.* 14 3–7. 10.5698/1535-7511-14.s2.3 24955068 PMC3966641

[B343] Vezzani A. (2015). Anti-inflammatory drugs in epilepsy: does it impact epileptogenesis? *Expert. Opin. Drug Saf* 14 583–592. 10.1517/14740338.2015.1010508 25645535

[B344] Vezzani A. Balosso S. Ravizza T. (2019). Neuroinflammatory pathways as treatment targets and biomarkers in epilepsy. *Nat. Rev. Neurol.* 15 459–472. 10.1038/s41582-019-0217-x 31263255

[B345] Vezzani A. Baram T. Z. (2007). New roles for interleukin-1 Beta in the mechanisms of epilepsy. *Epilepsy Curr.* 7 45–50. 10.1111/j.1535-7511.2007.00165.x 17505552 PMC1867084

[B346] Vezzani A. French J. Bartfai T. Baram T. Z. (2011). The role of inflammation in epilepsy. *Nat. Rev. Neurol.* 7, 31–40. 10.1038/nrneurol.2010.178 21135885 PMC3378051

[B347] Vezzani A. Fujinami R. S. White H. S. Preux P. M. Blümcke I. Sander J. W. (2016). Infections, inflammation and epilepsy. *Acta Neuropathol.* 131 211–234. 10.1007/s00401-015-1481-5 26423537 PMC4867498

[B348] Vezzani A. Viviani B. (2015). Neuromodulatory properties of inflammatory cytokines and their impact on neuronal excitability. *Neuropharmacology* 96 70–82. 10.1016/j.neuropharm.2014.10.027 25445483

[B349] Vilibic-Cavlek T. Savic V. Ferenc T. Mrzljak A. Barbic L. Bogdanic M. (2021). Lymphocytic Choriomeningitis-Emerging Trends of a Neglected Virus: a Narrative Review. *Trop. Med. Infect. Dis.* 2021:6. 10.3390/tropicalmed6020088 34070581 PMC8163193

[B350] Vincenti I. Merkler D. (2021). New advances in immune components mediating viral control in the CNS. *Curr. Opin. Virol.* 47 68–78. 10.1016/j.coviro.2021.02.001 33636592

[B351] Wakamoto H. Ohta M. Nakano N. Kunisue K. (2000). SPECT in focal enterovirus encephalitis: evidence for local cerebral vasculitis. *Pediatr. Neurol.* 23 429–431. 10.1016/s0887-8994(00)00206-x 11118800

[B352] Walker L. E. Sills G. J. Jorgensen A. Alapirtti T. Peltola J. Brodie M. J. (2022). High-mobility group box 1 as a predictive biomarker for drug-resistant epilepsy: a proof-of-concept study. *Epilepsia* 63 e1–e6. 10.1111/epi.17116 34747496

[B353] Walter R. D. (1969). Clinical aspects of temporal lobe epilepsy–a review. *Calif. Med.* 110 325–329. 4894817 PMC1503477

[B354] Waltl I. Kalinke U. (2022). Beneficial and detrimental functions of microglia during viral encephalitis. *Trends Neurosci.* 45 158–170. 10.1016/j.tins.2021.11.004 34906391

[B355] Waltl I. Käufer C. Bröer S. Chhatbar C. Ghita L. Gerhauser I. (2018a). Macrophage depletion by liposome-encapsulated clodronate suppresses seizures but not hippocampal damage after acute viral encephalitis. *Neurobiol. Dis.* 110 192–205. 10.1016/j.nbd.2017.12.001 29208406

[B356] Waltl I. Käufer C. Bröer S. Gerhauser I. Chatbar C. Kalinke U. (2018c). “Pharmacological modulation of monocyte infiltration into the CNS and development of acute seizures and epilepsy following Theiler’s virus infection in C57BL/6 mice,” in *Fourth N-RENNT Synmposium on Neuroinfectiology, February 12-13 2018* (Hannover).

[B357] Waltl I. Käufer C. Gerhauser I. Chhatbar C. Ghita L. Kalinke U. (2018b). Microglia have a protective role in viral encephalitis-induced seizure development and hippocampal damage. *Brain Behav. Immun.* 74 186–204. 10.1016/j.bbi.2018.09.006 30217535 PMC7111316

[B358] Wang C. Zhang M. Garcia G. Jr. Tian E. Cui Q. Chen X. (2021a). ApoE-Isoform-Dependent SARS-CoV-2 Neurotropism and Cellular Response. *Cell Stem Cell* 28 331–342. 10.1016/j.stem.2020.12.018 33450186 PMC7832490

[B359] Wang Z. Y. Wang P. G. An J. (2021b). The Multifaceted Roles of TAM Receptors during Viral Infection. *Virol. Sin.* 36 1–12. 10.1007/s12250-020-00264-9 32720213 PMC7973326

[B360] Wang H. Ward M. F. Fan X. G. Sama A. E. Li W. (2006). Potential role of high mobility group box 1 in viral infectious diseases. *Viral Immunol.* 19 3–9. 10.1089/vim.2006.19.3 16553546 PMC1782047

[B361] Wang J. Li J. (2021). Temporal lobe epilepsy associated with human herpes virus 6. *Acta Epileptol.* 3:10.

[B362] Wang Y. Ma L. Stipkovits L. Szathmary S. Li X. Liu Y. (2018). The Strategy of Picornavirus Evading Host Antiviral Responses: non-structural Proteins Suppress the Production of IFNs. *Front. Microbiol.* 9:2943. 10.3389/fmicb.2018.02943 30619109 PMC6297142

[B363] Weisgraber K. H. Mahley R. W. (1996). Human apolipoprotein E: the Alzheimer’s disease connection. *FASEB J.* 10 1485–1494. 10.1096/fasebj.10.13.8940294 8940294

[B364] Welzel L. Bergin D. H. Schidlitzki A. Twele F. Johne M. Klein P. (2021). Systematic evaluation of rationally chosen multitargeted drug combinations: a combination of low doses of levetiracetam, atorvastatin and ceftriaxone exerts antiepileptogenic effects in a mouse model of acquired epilepsy. *Neurobiol. Dis.* 149:105227. 10.1016/j.nbd.2020.105227 33347976

[B365] Wenzel J. Lampe J. Müller-Fielitz H. Schuster R. Zille M. Müller K. (2021). The SARS-CoV-2 main protease M(pro) causes microvascular brain pathology by cleaving NEMO in brain endothelial cells. *Nat. Neurosci.* 24 1522–1533. 10.1038/s41593-021-00926-1 34675436 PMC8553622

[B366] Whitton J. L. Cornell C. T. Feuer R. (2005). Host and virus determinants of picornavirus pathogenesis and tropism. *Nat. Rev. Microbiol.* 3 765–776. 10.1038/nrmicro1284 16205710

[B367] Wilcox K. S. West P. J. Metcalf C. S. (2020). The Current Approach of the Epilepsy Therapy Screening Program Contract Site for Identifying Improved Therapies for the Treatment of Pharmacoresistant Seizures in Epilepsy. *Neuropharmacology* 166:107811. 10.1016/j.neuropharm.2019.107811 31790717 PMC7054975

[B368] Wiley C. A. (2020). Emergent Viral Infections of the CNS. *J. Neuropathol. Exp. Neurol.* 79 823–842. 10.1093/jnen/nlaa054 32647884

[B369] Wilfert C. M. Buckley R. H. Mohanakumar T. Griffith J. F. Katz S. L. Whisnant J. K. (1977). Persistent and fatal central-nervous-system ECHOvirus infections in patients with agammaglobulinemia. *N. Engl. J. Med.* 296 1485–1489. 10.1056/NEJM197706302962601 301244

[B370] Williams A. E. Blakemore W. F. (1990). Monocyte-mediated entry of pathogens into the central nervous system. *Neuropathol. Appl. Neurobiol.* 16 377–392. 10.1111/j.1365-2990.1990.tb01274.x 2263314

[B371] Wilson J. C. Toovey S. Jick S. S. Meier C. R. (2015). Previously diagnosed influenza infections and the risk of developing epilepsy. *Epidemiol. Infect.* 143 2408–2415. 10.1017/S0950268814003380 25519212 PMC9150936

[B372] Wipfler P. Dunn N. Beiki O. Trinka E. Fogdell-Hahn A. (2018). The viral hypothesis of mesial temporal lobe epilepsy – is human herpes virus-6 the missing link? A systematic review and meta-analysis. *Seizure* 54, 33–40. 10.1016/j.seizure.2017.11.015 29195226

[B373] Wright R. Johnson D. Neumann M. Ksiazek T. G. Rollin P. Keech R. V. (1997). Congenital lymphocytic choriomeningitis virus syndrome: a disease that mimics congenital toxoplasmosis or Cytomegalovirus infection. *Pediatrics* 100:E9. 10.1542/peds.100.1.e9 9200383

[B374] Wu H. M. Huang C. C. Chen S. H. Liang Y. C. Tsai J. J. Hsieh C. L. (2003). Herpes simplex virus type 1 inoculation enhances hippocampal excitability and seizure susceptibility in mice. *Eur. J. Neurosci.* 18 3294–3304. 10.1111/j.1460-9568.2003.03075.x 14686902

[B375] Xing J. Wang K. Wei H. Wei D. (2018). Pathologic and molecular studies of enterovirus 71 infection in a fatal case from a recent epidemic in China: a case report. *Medicine* 97:e13447. 10.1097/MD.0000000000013447 30508963 PMC6283094

[B376] Xu Z. Xue T. Zhang Z. Wang X. Xu P. Zhang J. (2011). Role of signal transducer and activator of transcription-3 in up-regulation of GFAP after epilepsy. *Neurochem. Res.* 36 2208–2215. 10.1007/s11064-011-0576-1 21833841

[B377] Yachou Y. El Idrissi A. Belapasov V. Ait B. S. (2020). Neuroinvasion, neurotropic, and neuroinflammatory events of SARS-CoV-2: understanding the neurological manifestations in COVID-19 patients. *Neurol. Sci.* 41 2657–2669. 10.1007/s10072-020-04575-3 32725449 PMC7385206

[B378] Yamanaka G. Ishida Y. Kanou K. Suzuki S. Watanabe Y. Takamatsu T. (2021). Towards a Treatment for Neuroinflammation in Epilepsy: interleukin-1 Receptor Antagonist, Anakinra, as a Potential Treatment in Intractable Epilepsy. *Int. J. Mol. Sci.* 2021:22. 10.3390/ijms22126282 34208064 PMC8230637

[B379] Yamasaki R. Lu H. Butovsky O. Ohno N. Rietsch A. M. Cialic R. (2014). Differential roles of microglia and monocytes in the inflamed central nervous system. *J. Exp. Med.* 211 1533–1549. 10.1084/jem.20132477 25002752 PMC4113947

[B380] Yang F. Du J. Hu Y. Wang X. Xue Y. Dong J. (2011). Enterovirus coinfection during an outbreak of hand, foot, and mouth disease in Shandong, China. *Clin. Infect. Dis.* 53 400–401. 10.1093/cid/cir346 21785005

[B381] Yu P. Bao L. Xu L. Li F. Lv Q. Deng W. (2017). Neurotropism In Vitro and Mouse Models of Severe and Mild Infection with Clinical Strains of Enterovirus 71. *Viruses* 2017:9. 10.3390/v9110351 29156632 PMC5707558

[B382] Zelano J. Westman G. (2020). Epilepsy after brain infection in adults: A register-based population-wide study. *Neurology* 95 e3213–e3220. 10.1212/WNL.0000000000010954 32989110

[B383] Zhang P. Yang Y. Zou J. Yang X. Liu Q. Chen Y. (2020). Seizures and epilepsy secondary to viral infection in the central nervous system. *Acta Epileptol.* 2:12.

[B384] Zhang X. M. Mao X. J. Zhang H. L. Zheng X. Y. Pham T. Adem A. (2012). Overexpression of apolipoprotein E4 increases kainic-acid-induced hippocampal neurodegeneration. *Exp. Neurol.* 233 323–332. 22079154 10.1016/j.expneurol.2011.10.024

[B385] Zhang Z. Y. Sun B. L. Liu J. K. Yang M. F. Li D. W. Fang J. (2015). Activation of mGluR5 Attenuates Microglial Activation and Neuronal Apoptosis in Early Brain Injury After Experimental Subarachnoid Hemorrhage in Rats. *Neurochem. Res.* 40 1121–1132. 10.1007/s11064-015-1572-7 25846008

[B386] Zhao L. Wu Y. P. Qi J. L. Liu Y. Q. Zhang K. Li W. L. (2018). Efficacy of levetiracetam compared with phenytoin in prevention of seizures in brain injured patients: a meta-analysis. *Medicine* 97:e13247. 10.1097/MD.0000000000013247 30508910 PMC6283080

[B387] Zheng M. Li S. Hogan R. E. Yang M. (2020). Arbovirus and seizures. *Acta Epileptol.* 2:17.

[B388] Zucchini S. Pittaluga A. Brocca-Cofano E. Summa M. Fabris M. De Michele R. (2013). Increased excitability in tat-transgenic mice: role of tat in HIV-related neurological disorders. *Neurobiol. Dis.* 55 110–119. 10.1016/j.nbd.2013.02.004 23454193

